# Supplementary figures and images for: S-palmitoylation modulates ATG2-dependent non-vesicular lipid transport during starvation-induced autophagy (part 1 of 2)
Source: EMBO J. 2025 Mar 24;44(9):2596–619. doi: 10.1038/s44318-025-00410-7 (PMC12048663; doi:10.1038/s44318-025-00410-7)

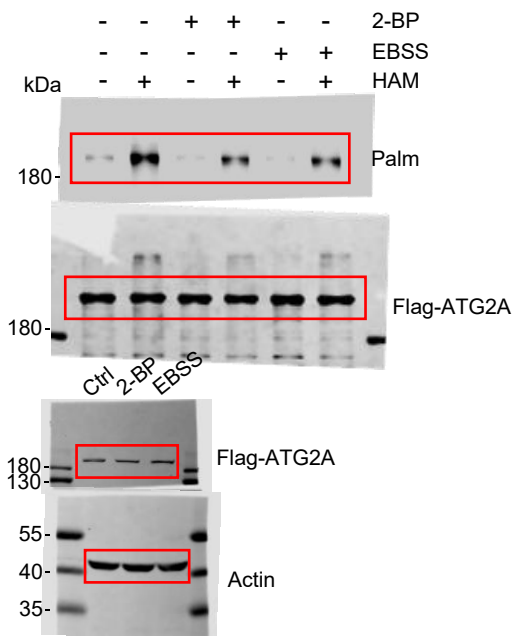

Fig 1A

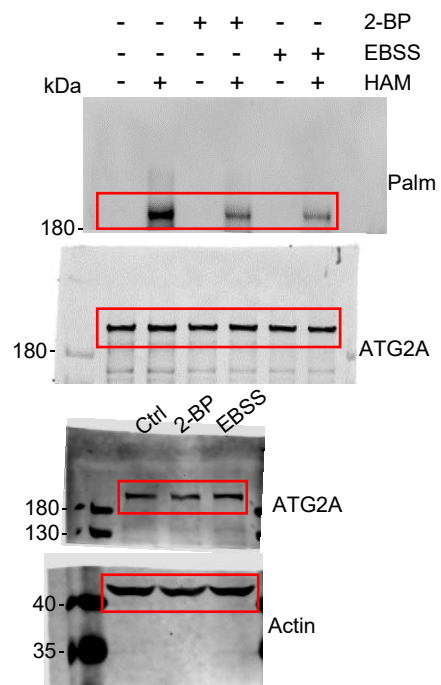

Fig 1B

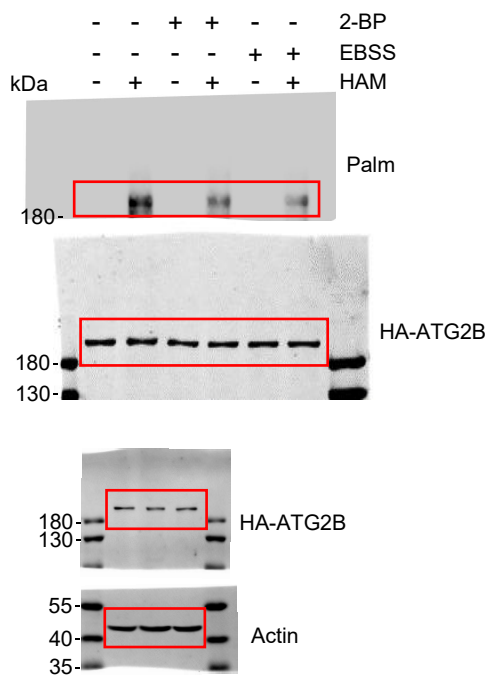

Fig 1C

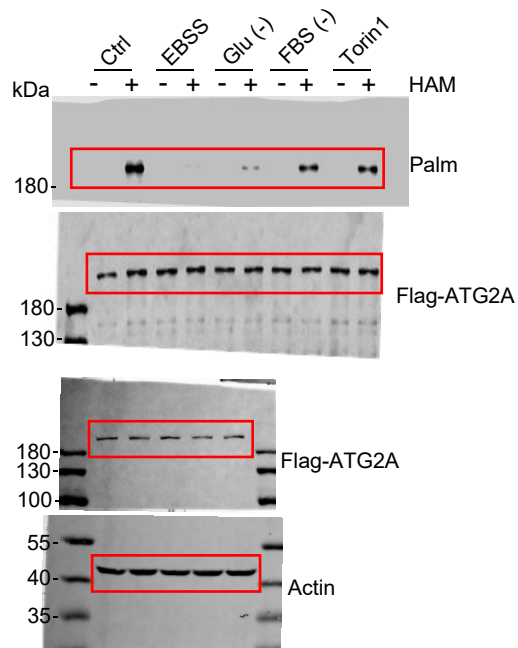

Fig 1E

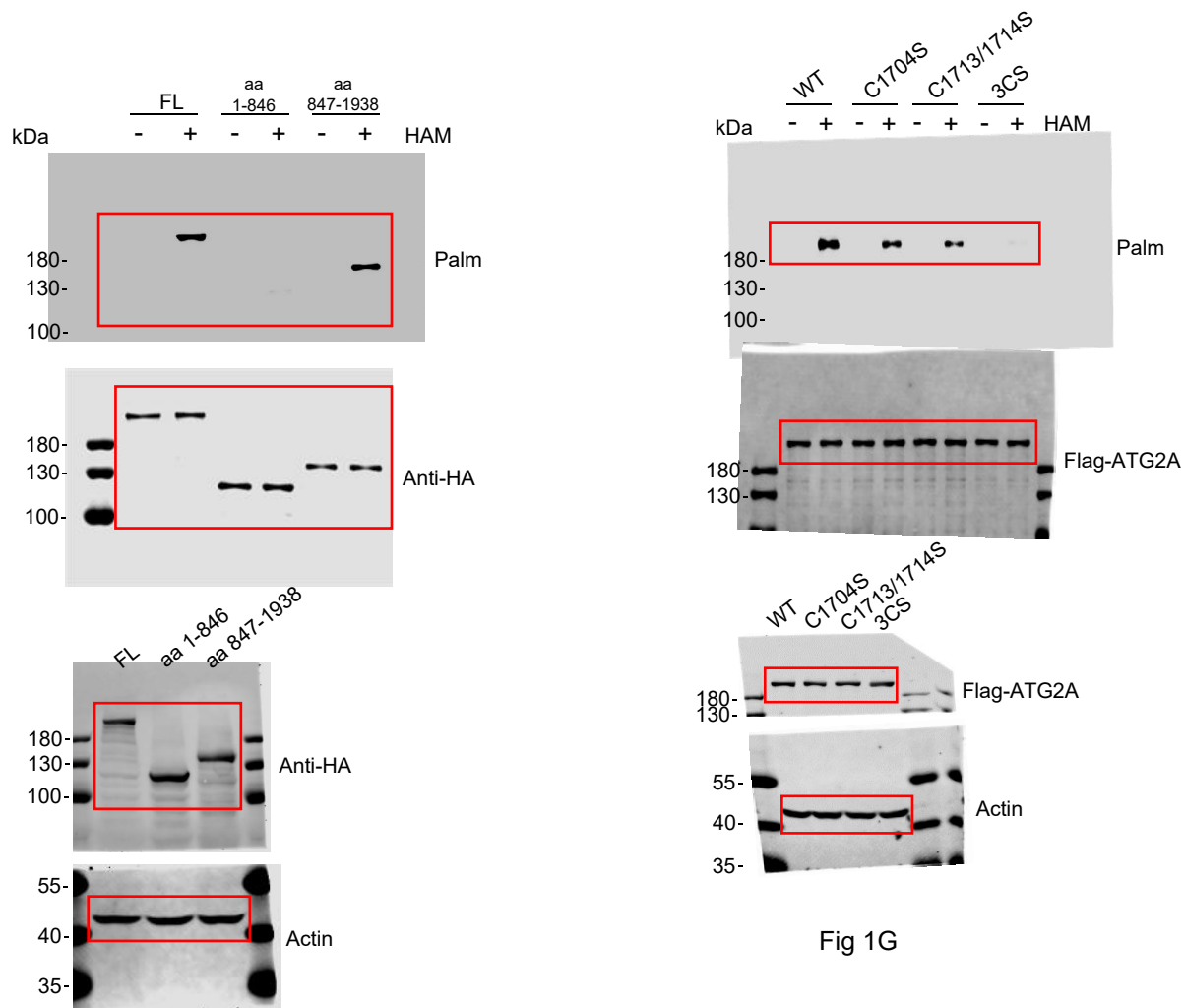

Fig 1F

Fig 1G

Supplement: Supplementary file 6 — Source data Fig. 1 [file 44318_2025_410_MOESM6_ESM.zip › Source Data For Figure 1/1A-C, E-G _western blot/Figure 1.pdf]

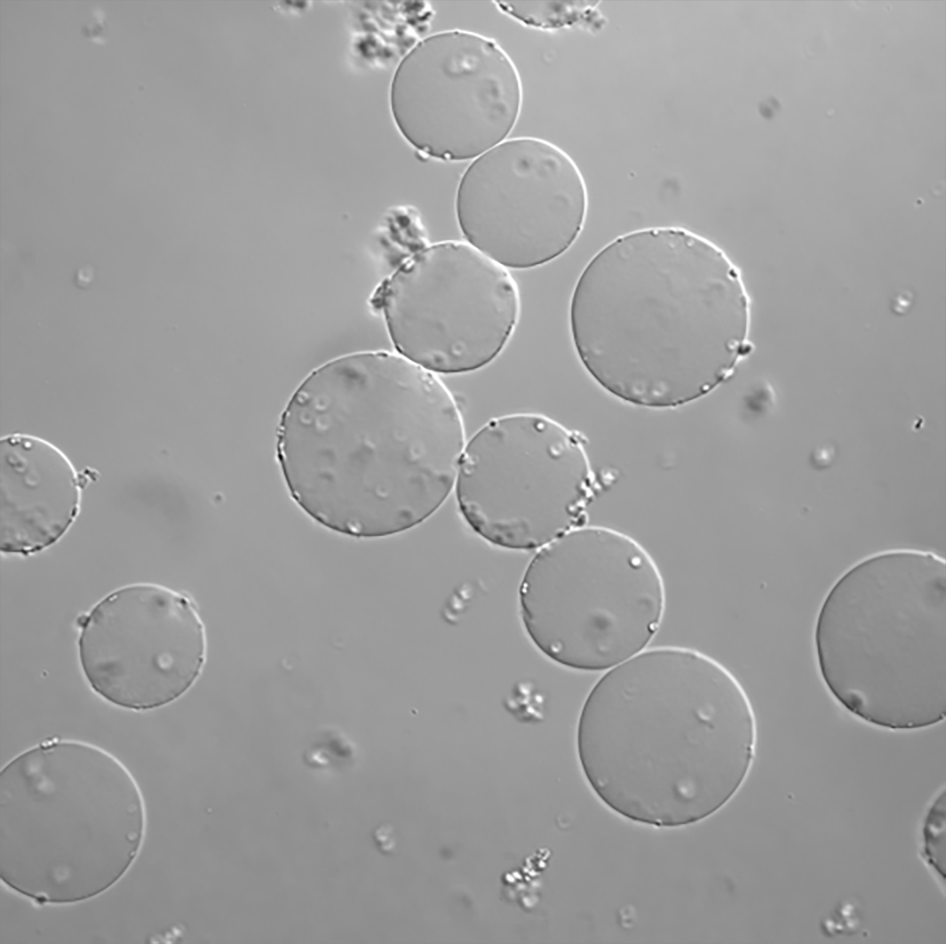

Supplement: Supplementary file 6 — Source data Fig. 1 [file 44318_2025_410_MOESM6_ESM.zip › Source Data For Figure 1/1D _microscopy/1D/1. Flag-vec + 17-ODYA_DIC.tif]

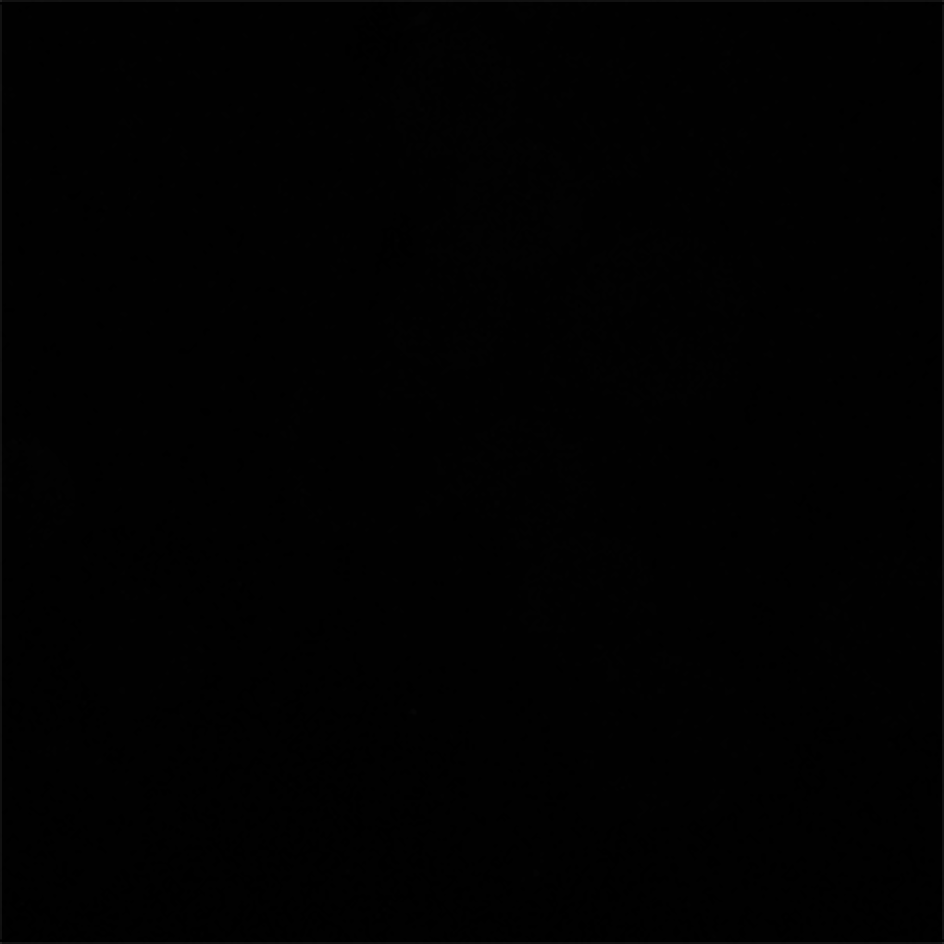

Supplement: Supplementary file 6 — Source data Fig. 1 [file 44318_2025_410_MOESM6_ESM.zip › Source Data For Figure 1/1D _microscopy/1D/1. Flag-vec + 17-ODYA_Rhodamine.tif]

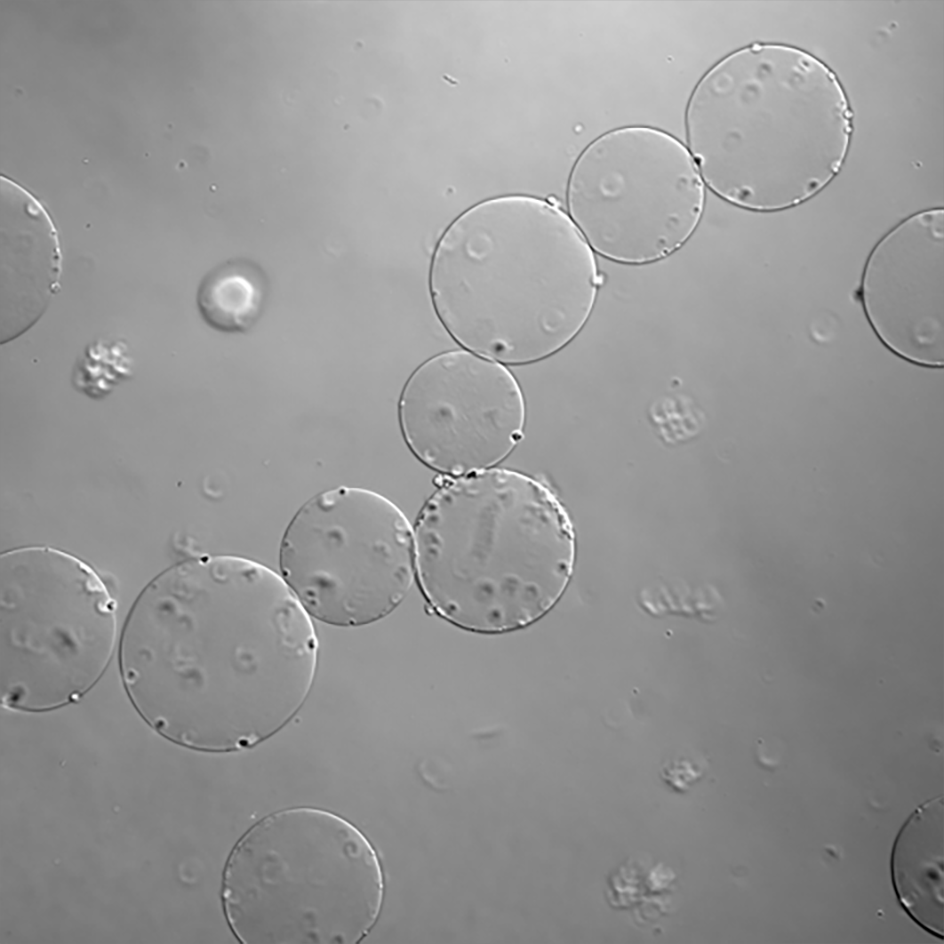

Supplement: Supplementary file 6 — Source data Fig. 1 [file 44318_2025_410_MOESM6_ESM.zip › Source Data For Figure 1/1D _microscopy/1D/2. Flag-ATG2A + 17-ODYA_DIC.tif]

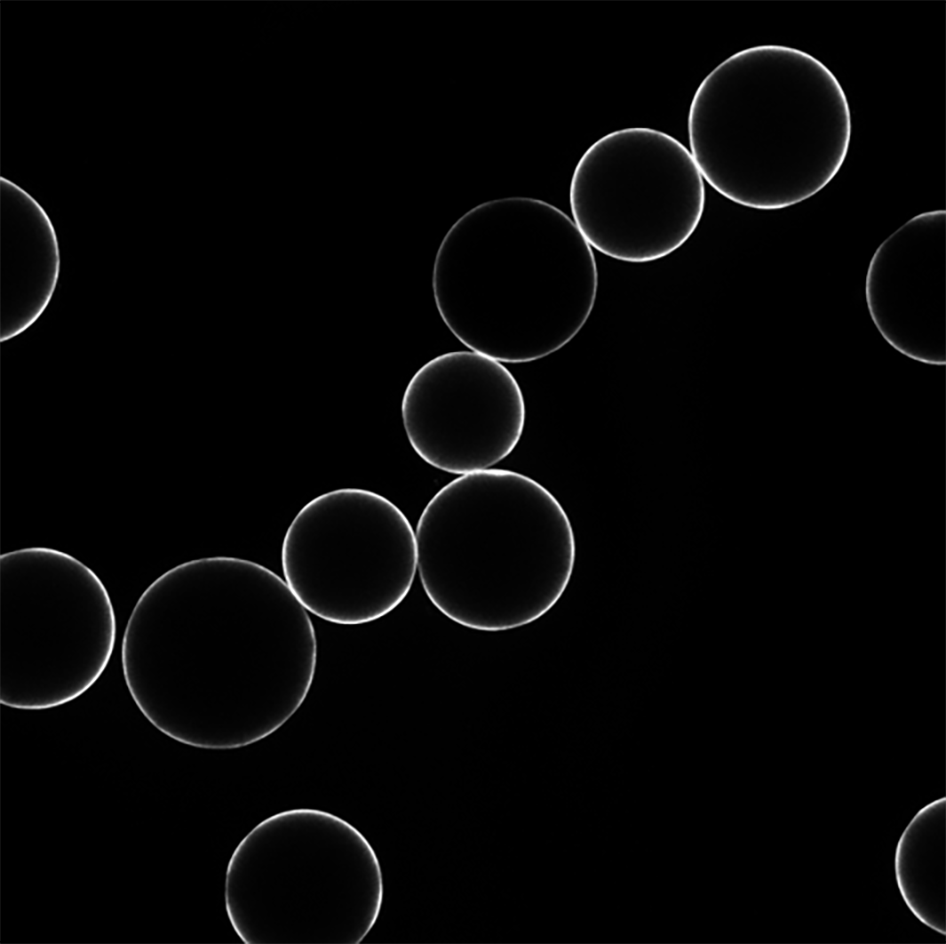

Supplement: Supplementary file 6 — Source data Fig. 1 [file 44318_2025_410_MOESM6_ESM.zip › Source Data For Figure 1/1D _microscopy/1D/2. Flag-ATG2A + 17-ODYA_Rhodamine.tif]

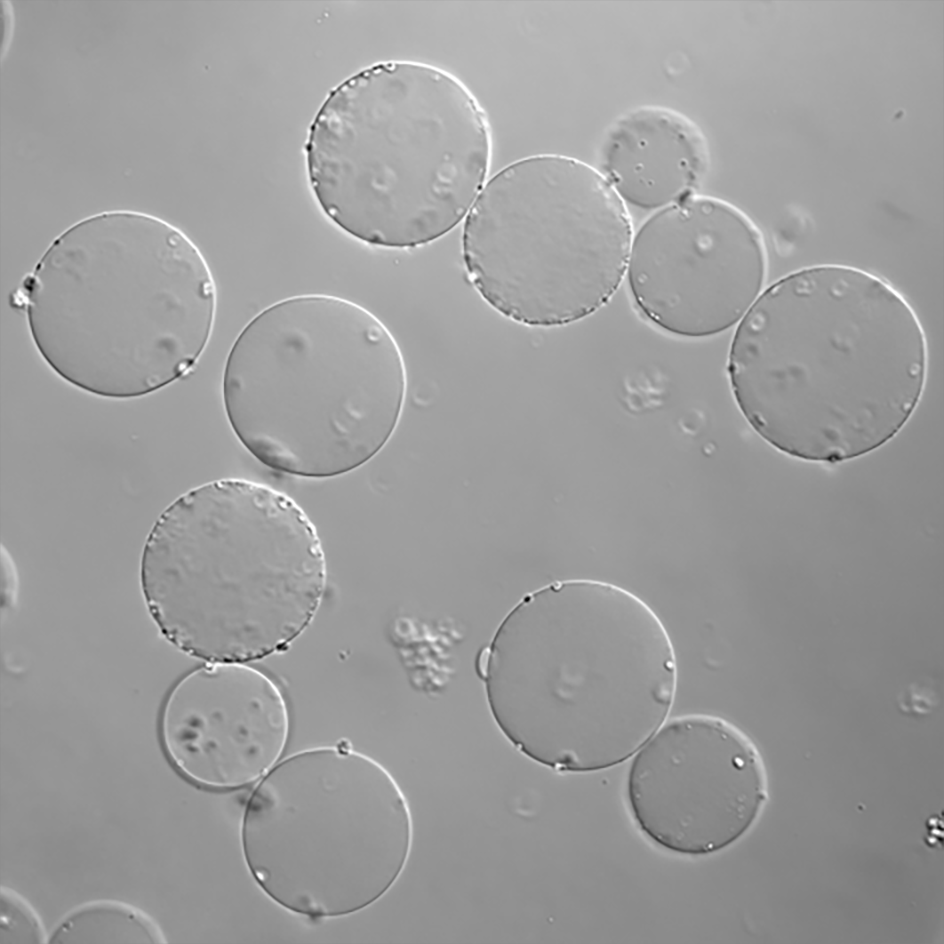

Supplement: Supplementary file 6 — Source data Fig. 1 [file 44318_2025_410_MOESM6_ESM.zip › Source Data For Figure 1/1D _microscopy/1D/3. Flag-ATG2A + PA_DIC.tif]

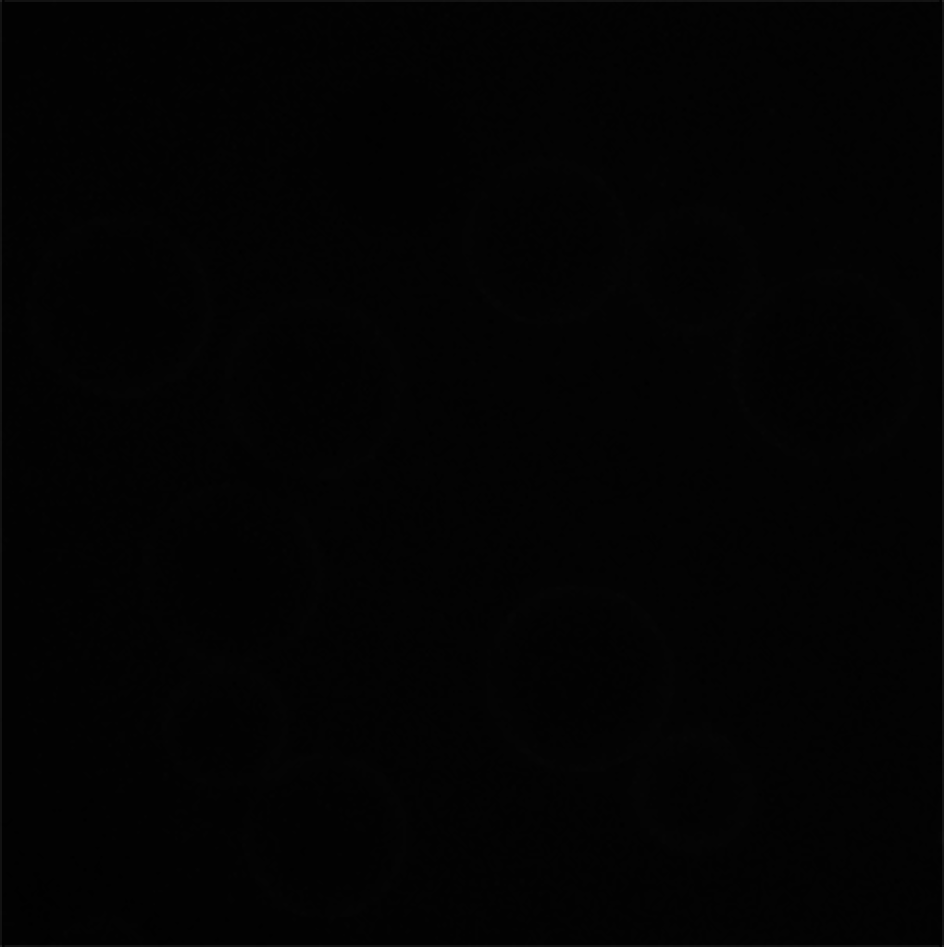

Supplement: Supplementary file 6 — Source data Fig. 1 [file 44318_2025_410_MOESM6_ESM.zip › Source Data For Figure 1/1D _microscopy/1D/3. Flag-ATG2A + PA_Rhodamine.tif]

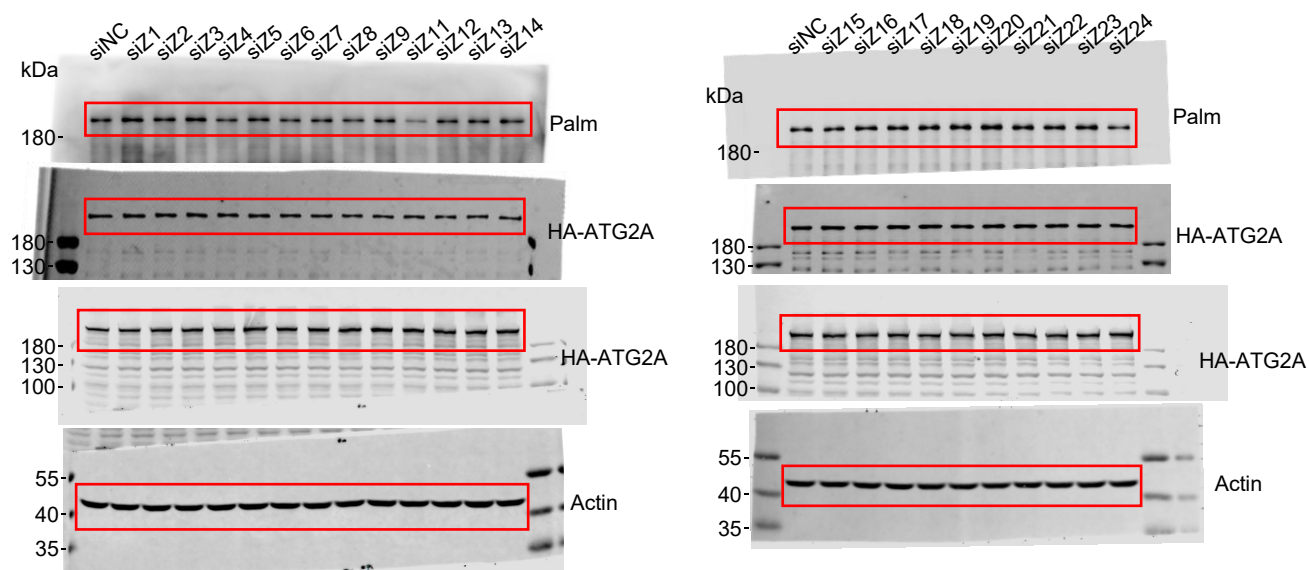

Fig 2A

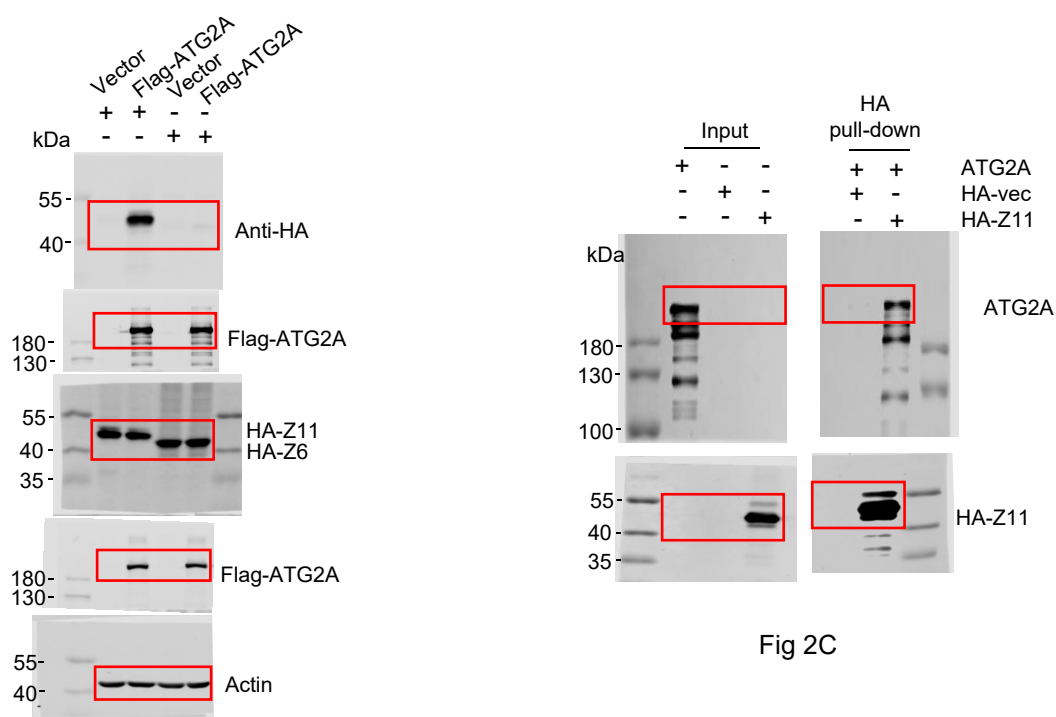

Fig 2C

Fig 2B

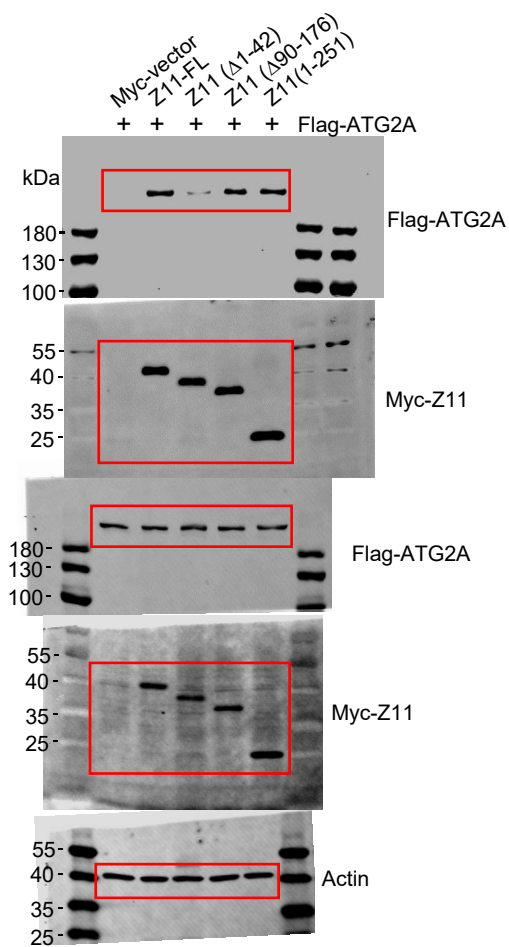

Fig 2E

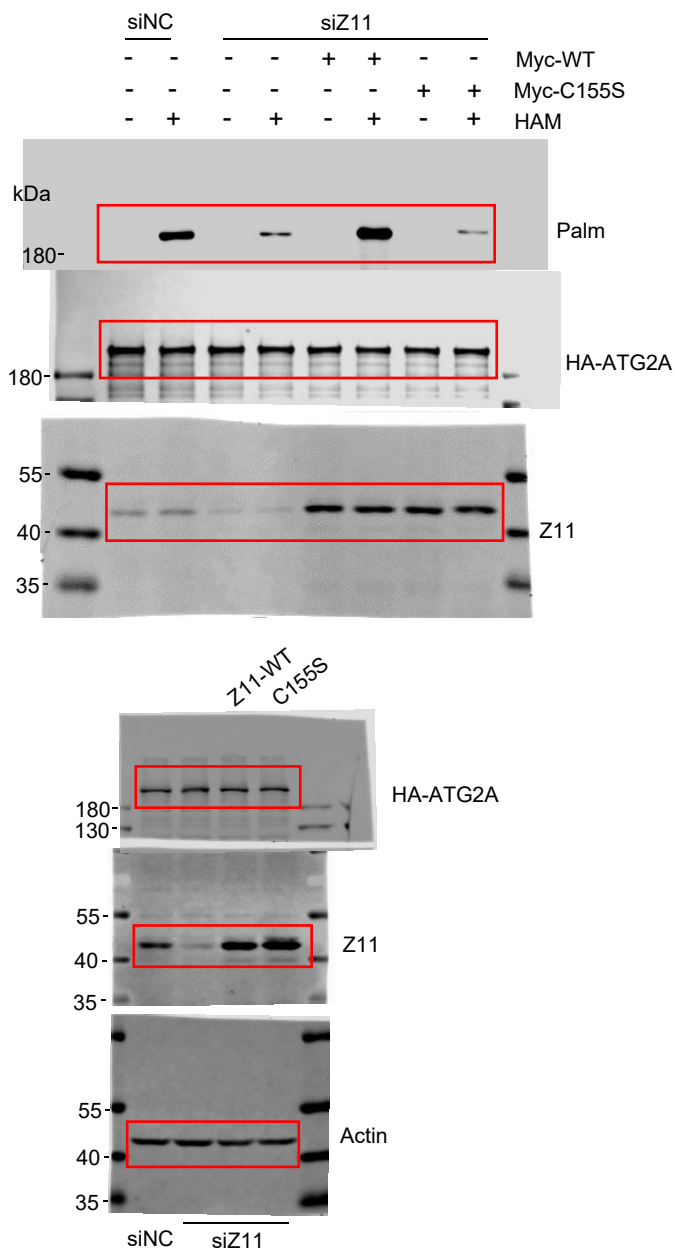

Fig 2F

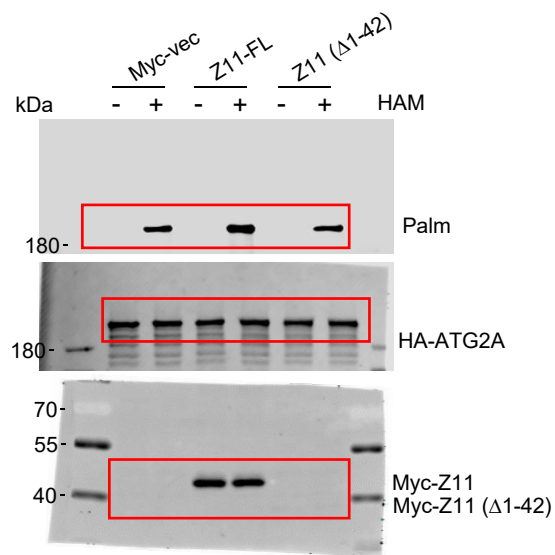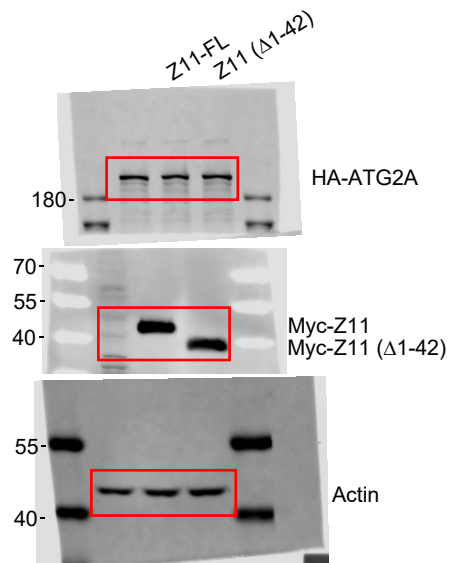

Fig 2G

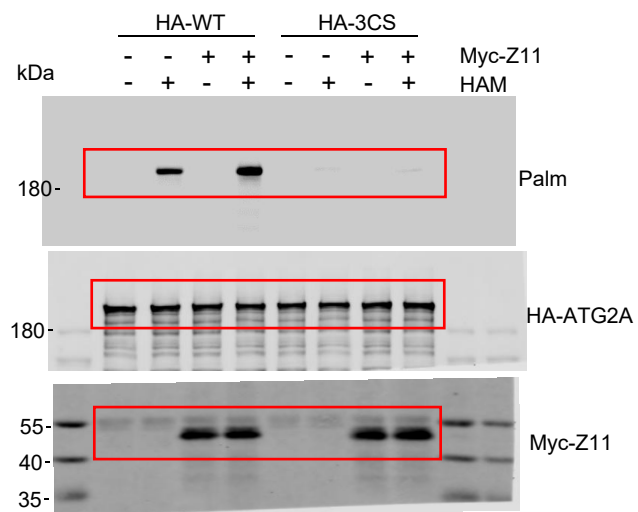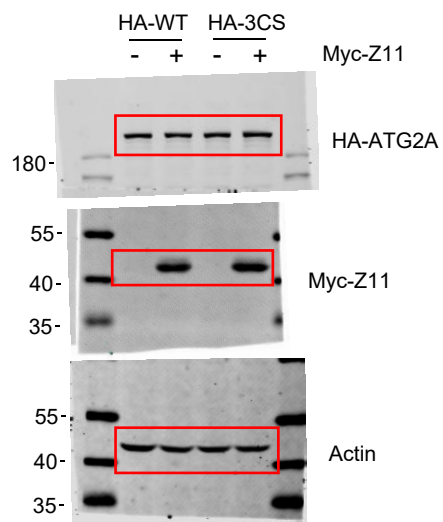

Fig 2H

Supplement: Supplementary file 7 — Source data Fig. 2 [file 44318_2025_410_MOESM7_ESM.zip › Source Data For Figure 2/2A, B, C, E-H _western blot/Figure 2.pdf]

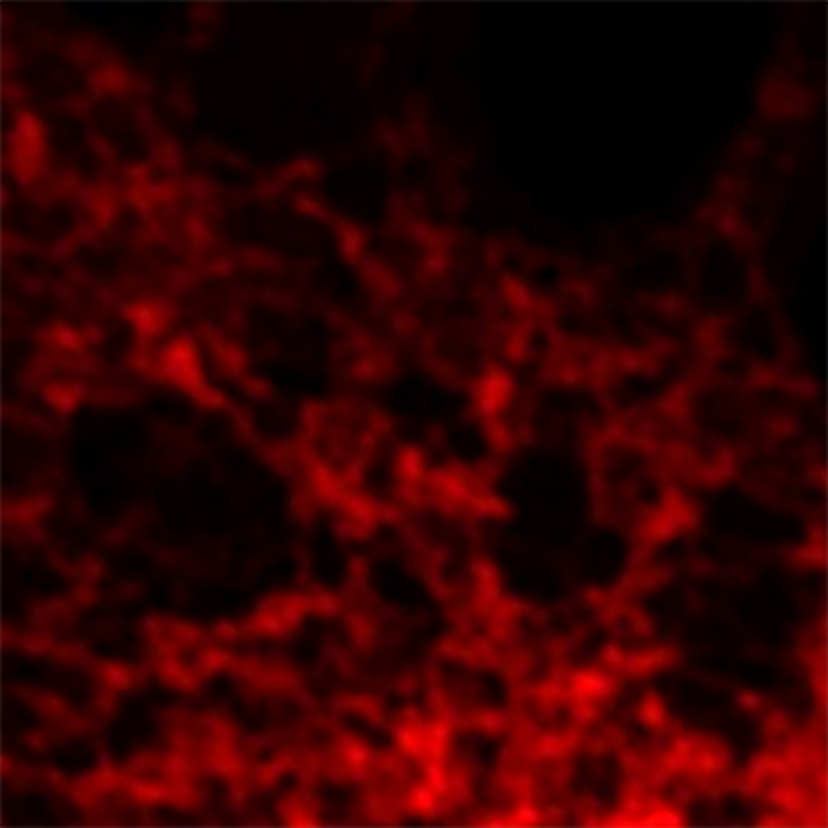

Supplement: Supplementary file 7 — Source data Fig. 2 [file 44318_2025_410_MOESM7_ESM.zip › Source Data For Figure 2/2D _microscopy/2D/Cherry-Z11.tif]

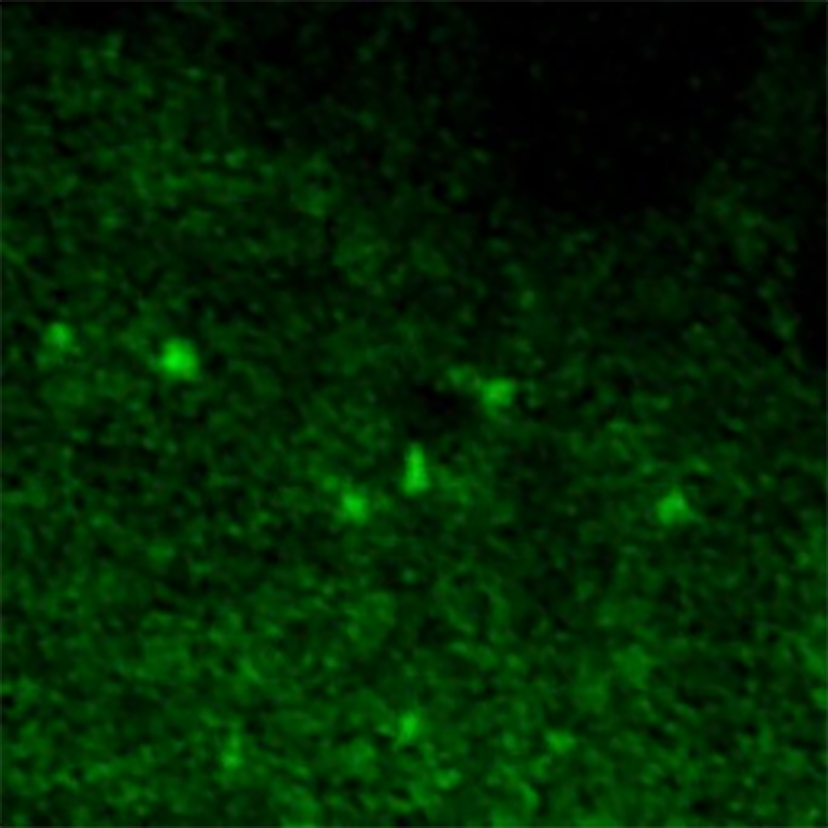

Supplement: Supplementary file 7 — Source data Fig. 2 [file 44318_2025_410_MOESM7_ESM.zip › Source Data For Figure 2/2D _microscopy/2D/GFP-ATG2A.tif]

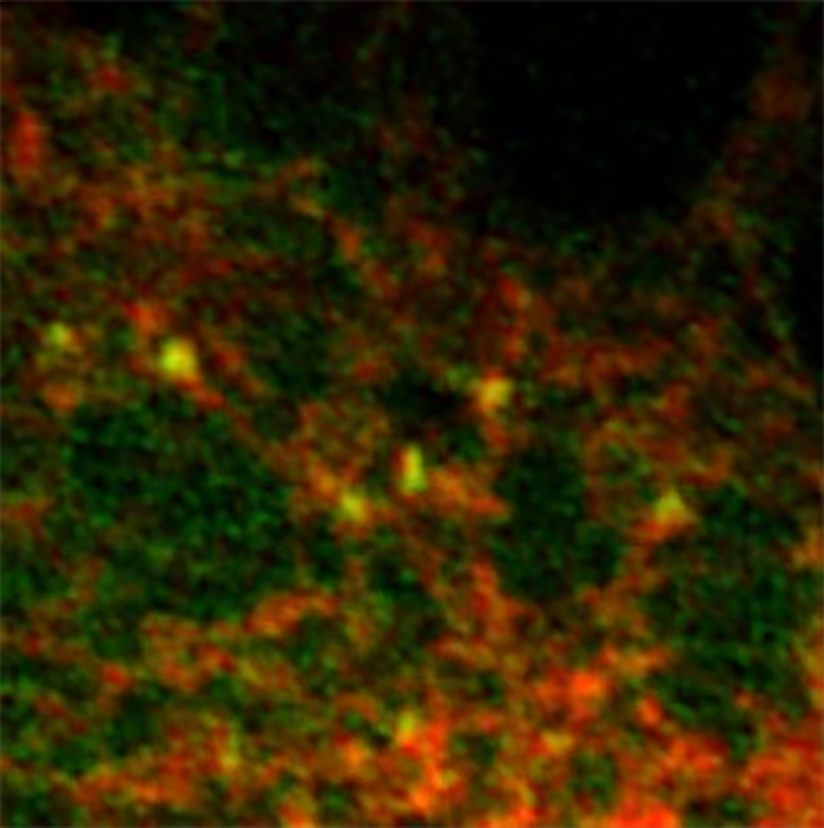

Supplement: Supplementary file 7 — Source data Fig. 2 [file 44318_2025_410_MOESM7_ESM.zip › Source Data For Figure 2/2D _microscopy/2D/Merged.tif]

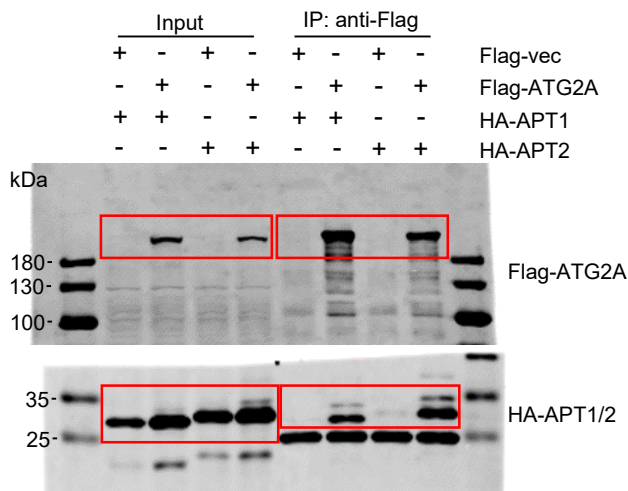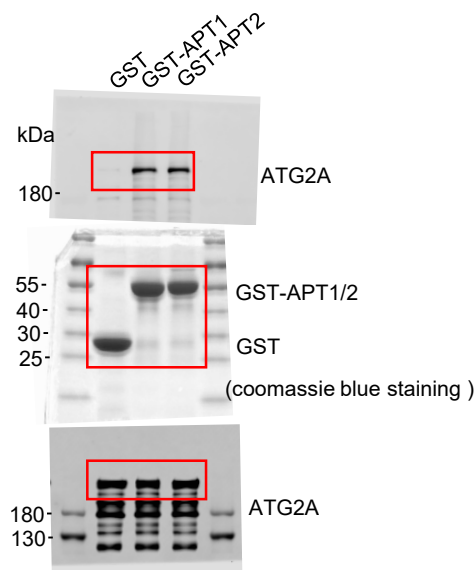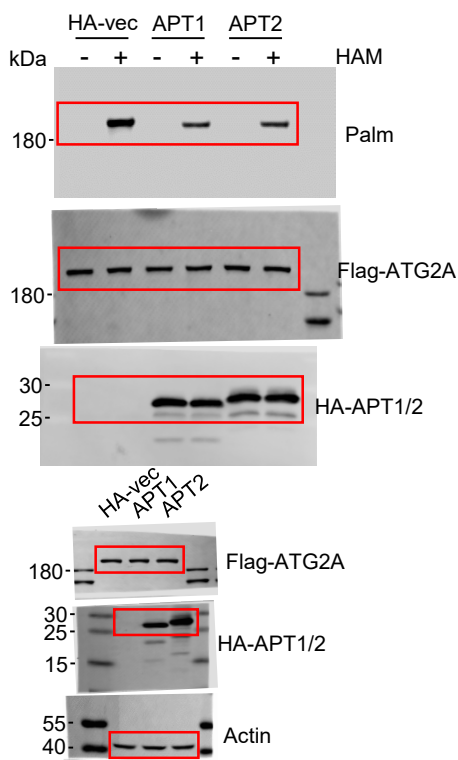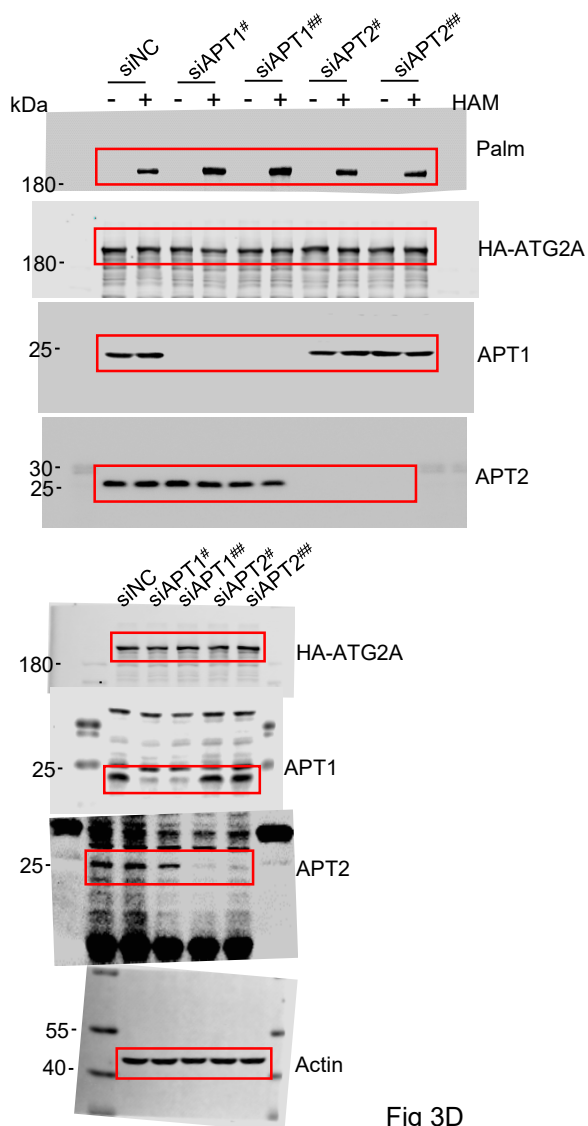

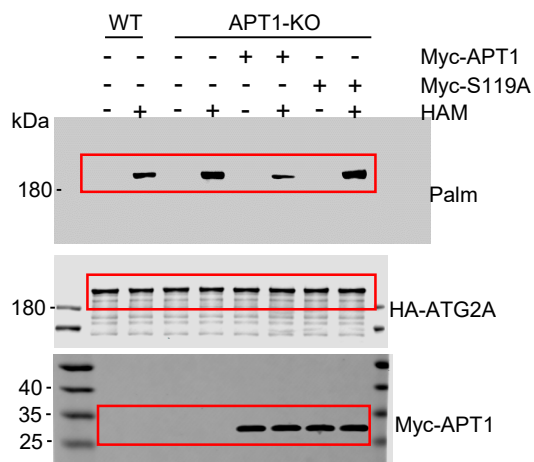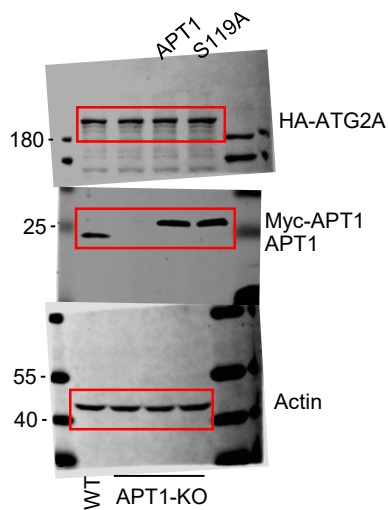

Fig 3E

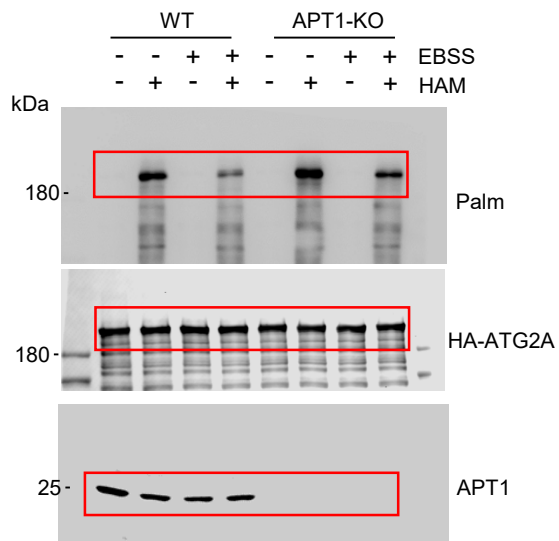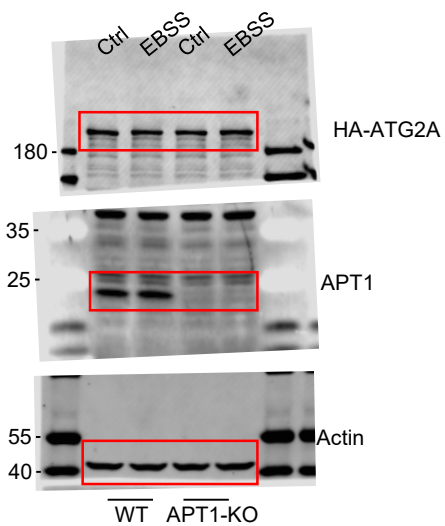

Fig 3F

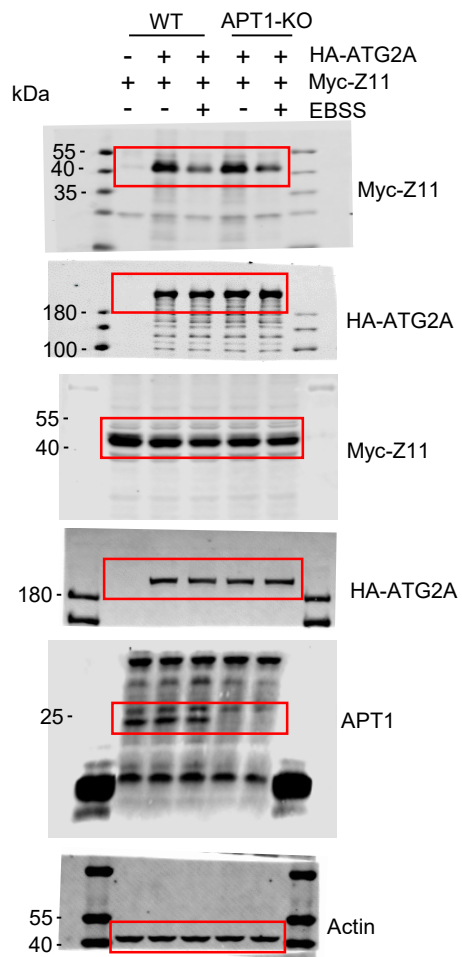

Fig 3G

Supplement: Supplementary file 8 — Source data Fig. 3 [file 44318_2025_410_MOESM8_ESM.zip › Source Data For Figure 3/3A-G _western blot/Figure 3.pdf]

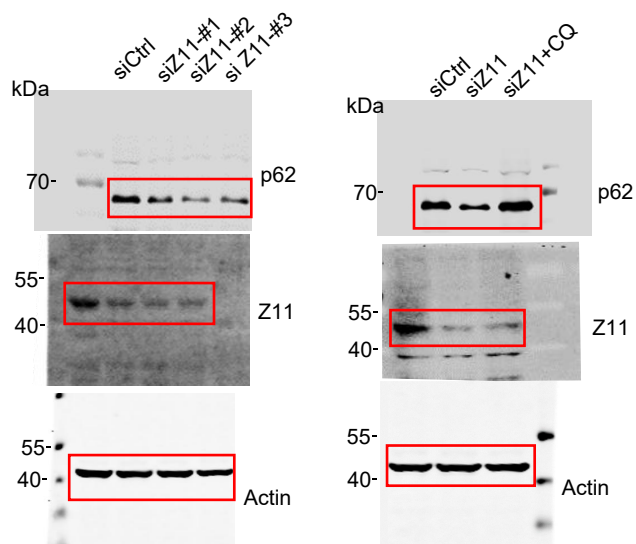

Fig 4A

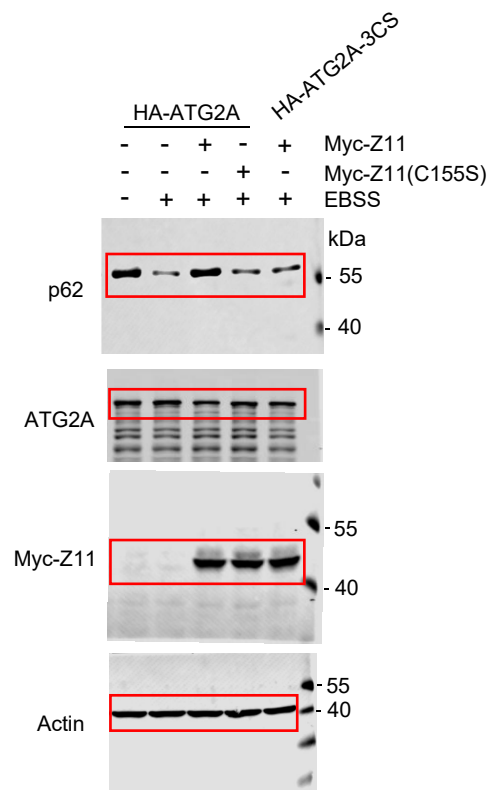

Fig 4C

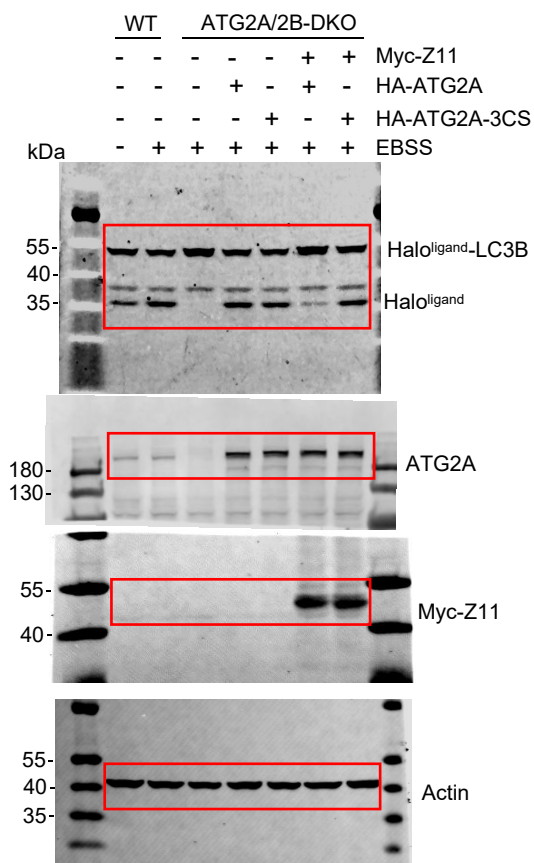

Fig 4H

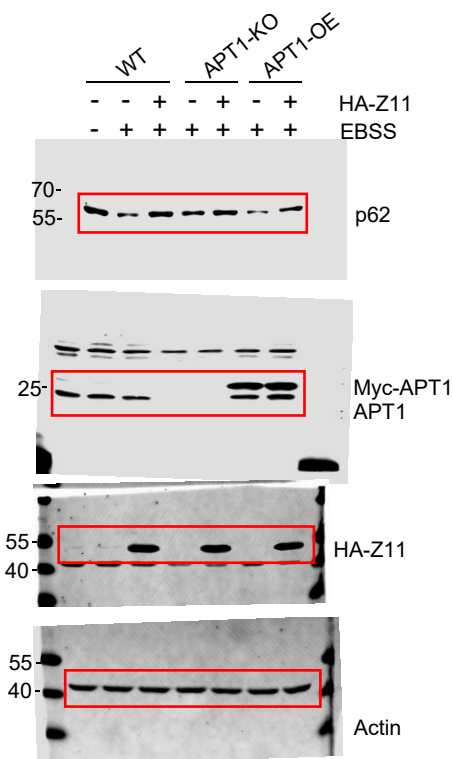

Fig 4I

Supplement: Supplementary file 9 — Source data Fig. 4 [file 44318_2025_410_MOESM9_ESM.zip › Source Data For Figure 4/4A, C, H, I _western blot/Figure 4.pdf]

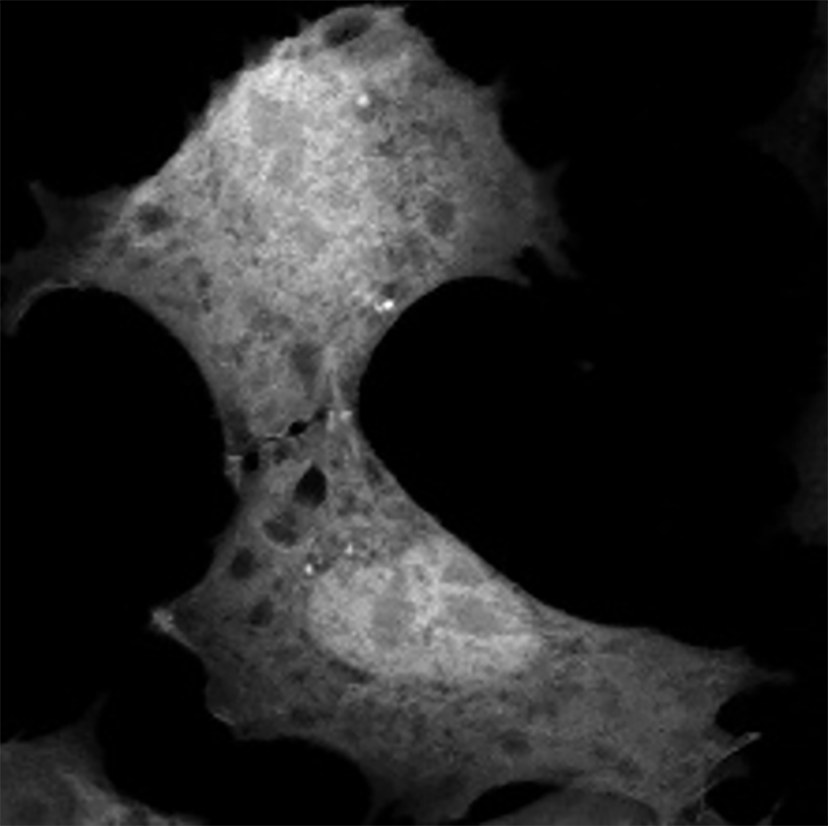

Supplement: Supplementary file 9 — Source data Fig. 4 [file 44318_2025_410_MOESM9_ESM.zip › Source Data For Figure 4/4D, F, J _microscopy/4D/1. WT_ctrl_GFP-LC3B.tif]

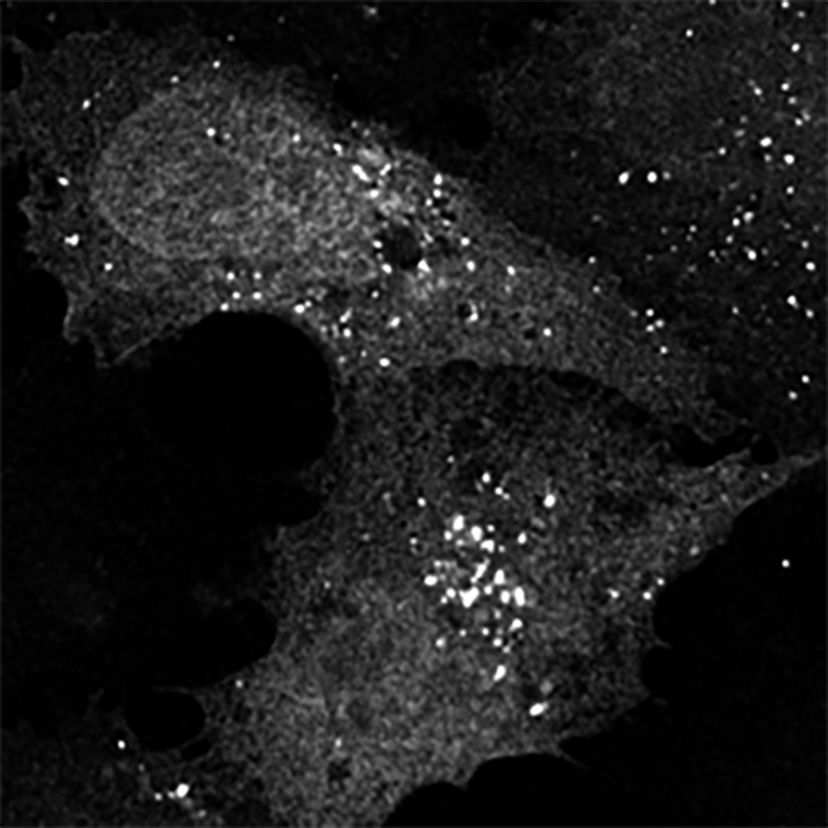

Supplement: Supplementary file 9 — Source data Fig. 4 [file 44318_2025_410_MOESM9_ESM.zip › Source Data For Figure 4/4D, F, J _microscopy/4D/2. WT_EBSS_GFP-LC3B.tif]

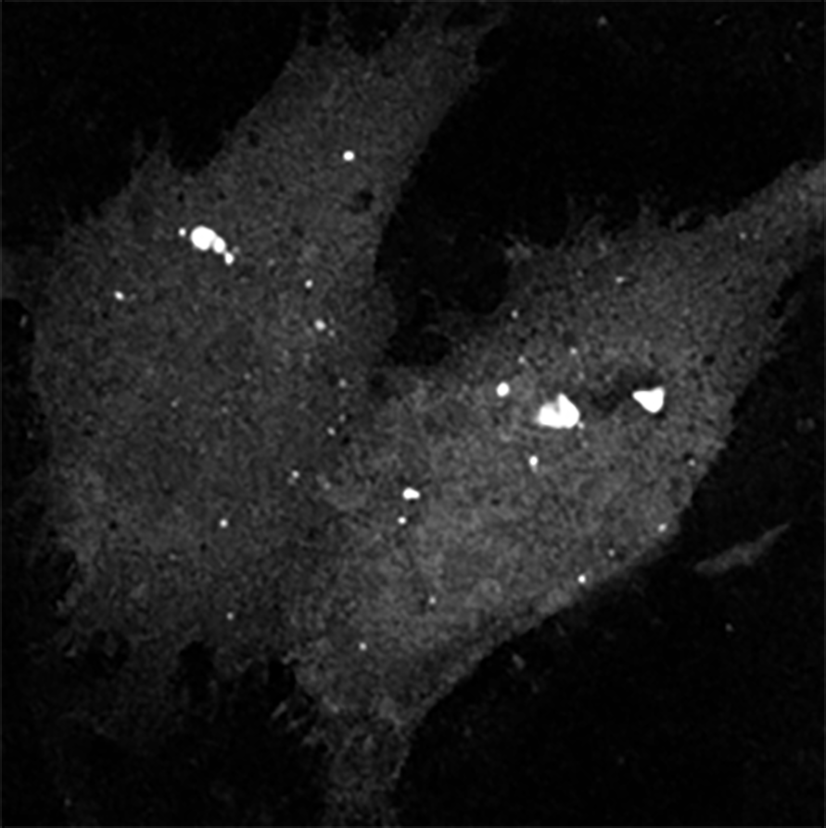

Supplement: Supplementary file 9 — Source data Fig. 4 [file 44318_2025_410_MOESM9_ESM.zip › Source Data For Figure 4/4D, F, J _microscopy/4D/3. 2A2B-DKO + HA-vec_EBSS_GFP-LC3B.tif]

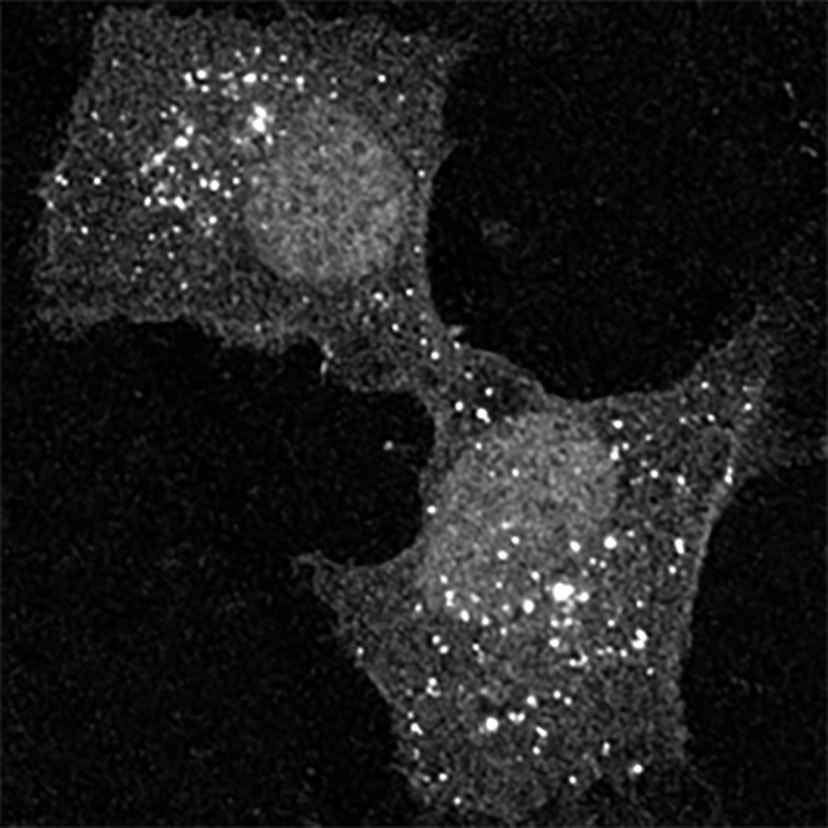

Supplement: Supplementary file 9 — Source data Fig. 4 [file 44318_2025_410_MOESM9_ESM.zip › Source Data For Figure 4/4D, F, J _microscopy/4D/4. 2A2B-DKO + HA-WT_EBSS_GFP-LC3B.tif]

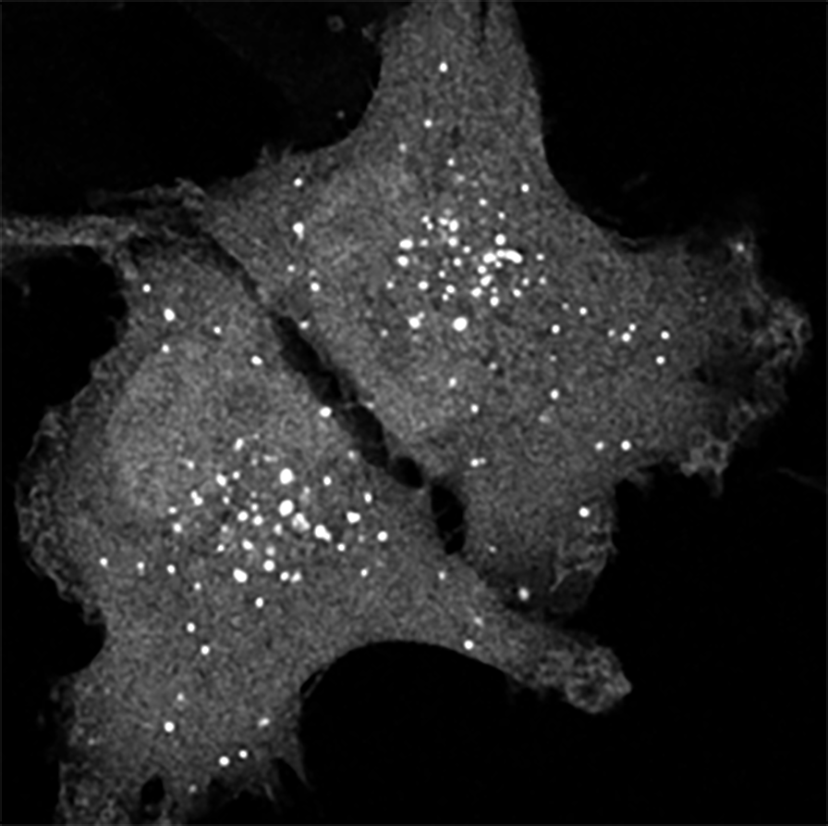

Supplement: Supplementary file 9 — Source data Fig. 4 [file 44318_2025_410_MOESM9_ESM.zip › Source Data For Figure 4/4D, F, J _microscopy/4D/5. 2A2B-DKO + HA-3CS_EBSS_GFP-LC3B.tif]

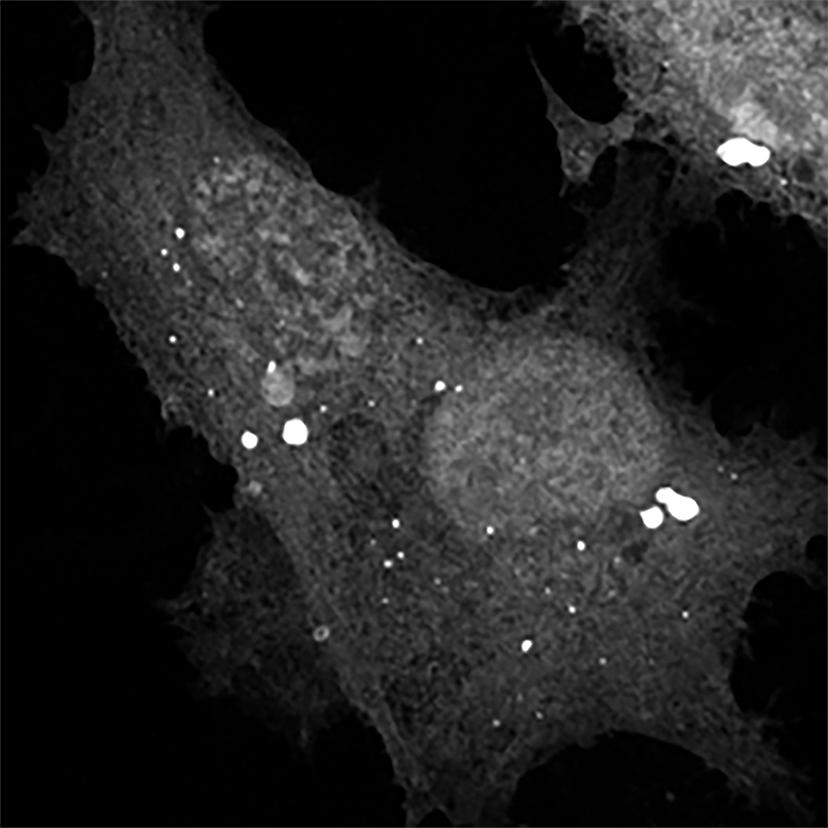

Supplement: Supplementary file 9 — Source data Fig. 4 [file 44318_2025_410_MOESM9_ESM.zip › Source Data For Figure 4/4D, F, J _microscopy/4D/6. 2A2B-DKO + HA-WT + Myc-Z11_EBSS_GFP-LC3B.tif]

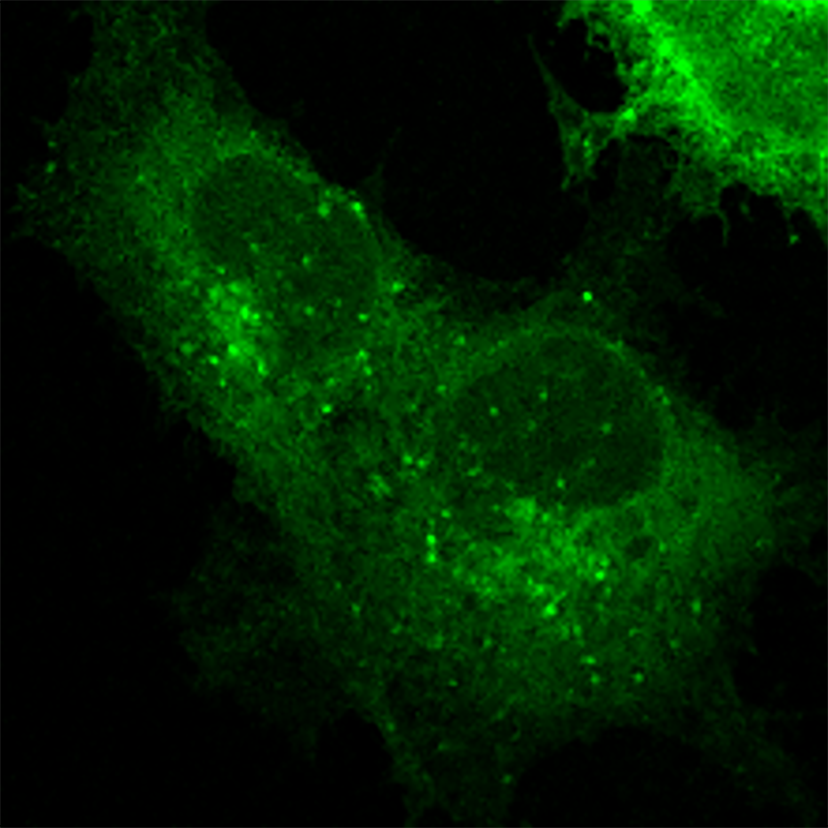

Supplement: Supplementary file 9 — Source data Fig. 4 [file 44318_2025_410_MOESM9_ESM.zip › Source Data For Figure 4/4D, F, J _microscopy/4D/6. 2A2B-DKO + HA-WT + Myc-Z11_EBSS_Myc-Z11.tif]

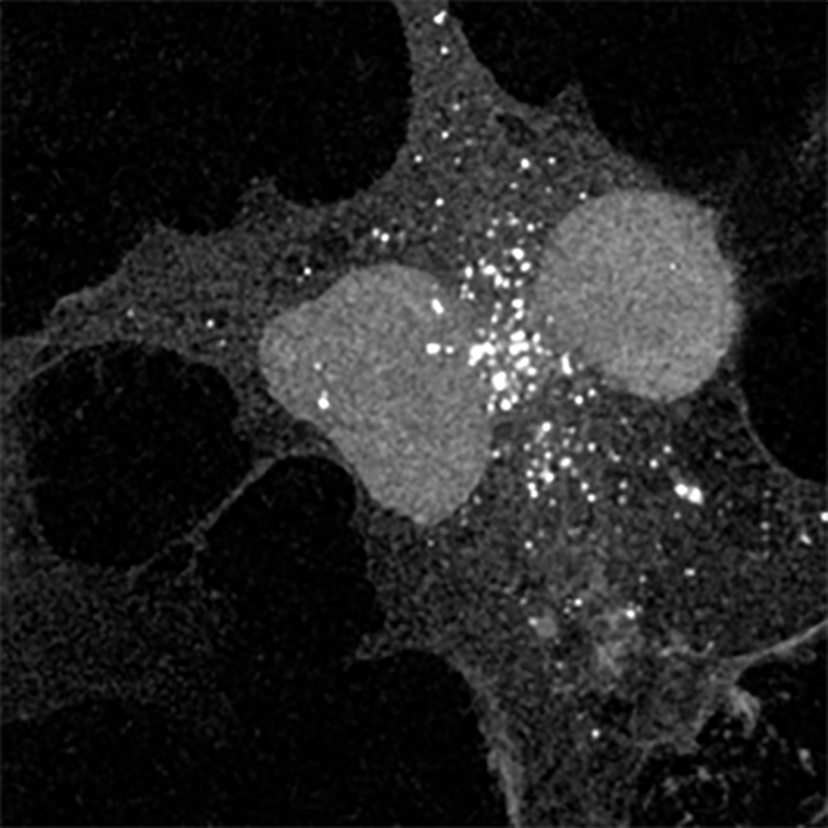

Supplement: Supplementary file 9 — Source data Fig. 4 [file 44318_2025_410_MOESM9_ESM.zip › Source Data For Figure 4/4D, F, J _microscopy/4D/7. 2A2B-DKO + HA-3CS + Myc-Z11_EBSS_GFP-LC3B.tif]

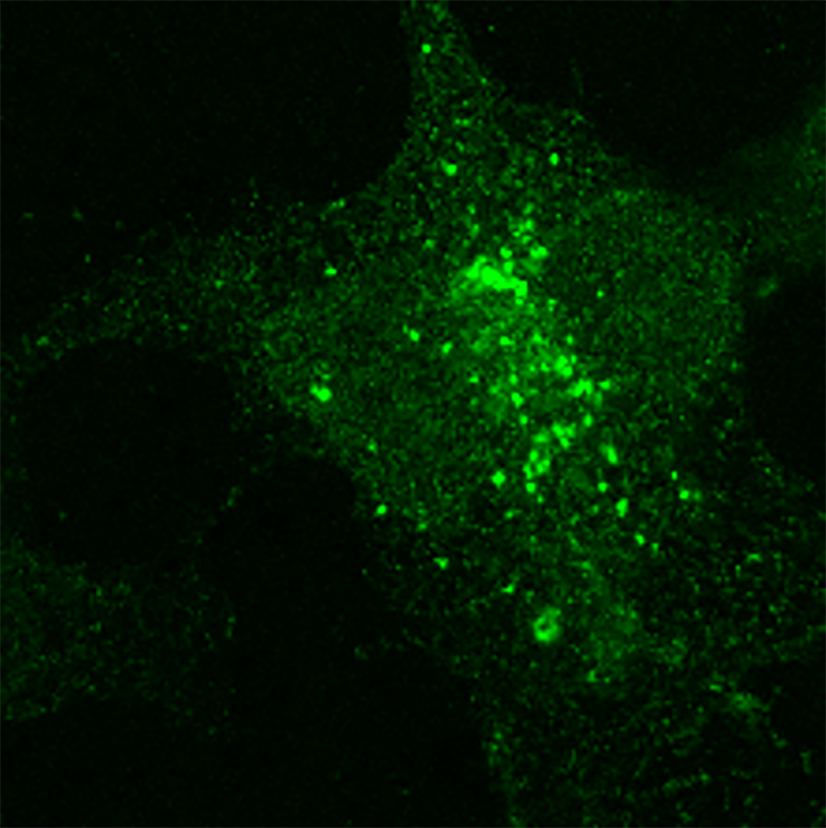

Supplement: Supplementary file 9 — Source data Fig. 4 [file 44318_2025_410_MOESM9_ESM.zip › Source Data For Figure 4/4D, F, J _microscopy/4D/7. 2A2B-DKO + HA-3CS + Myc-Z11_EBSS_Myc-Z11.tif]

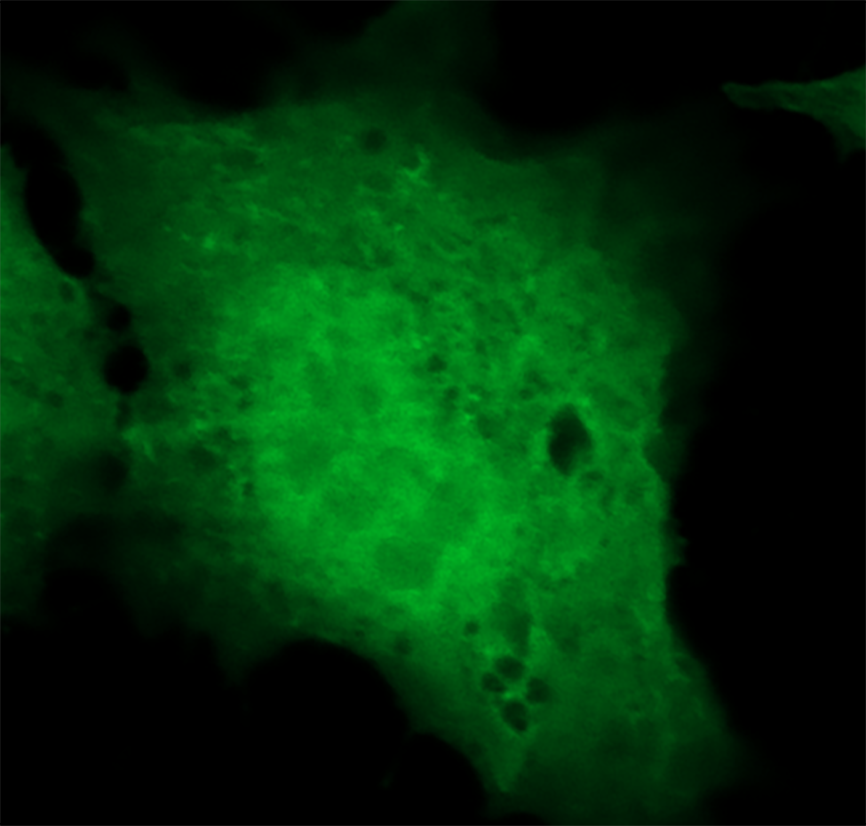

Supplement: Supplementary file 9 — Source data Fig. 4 [file 44318_2025_410_MOESM9_ESM.zip › Source Data For Figure 4/4D, F, J _microscopy/4F/1. WT_ctrl/GFP.tif]

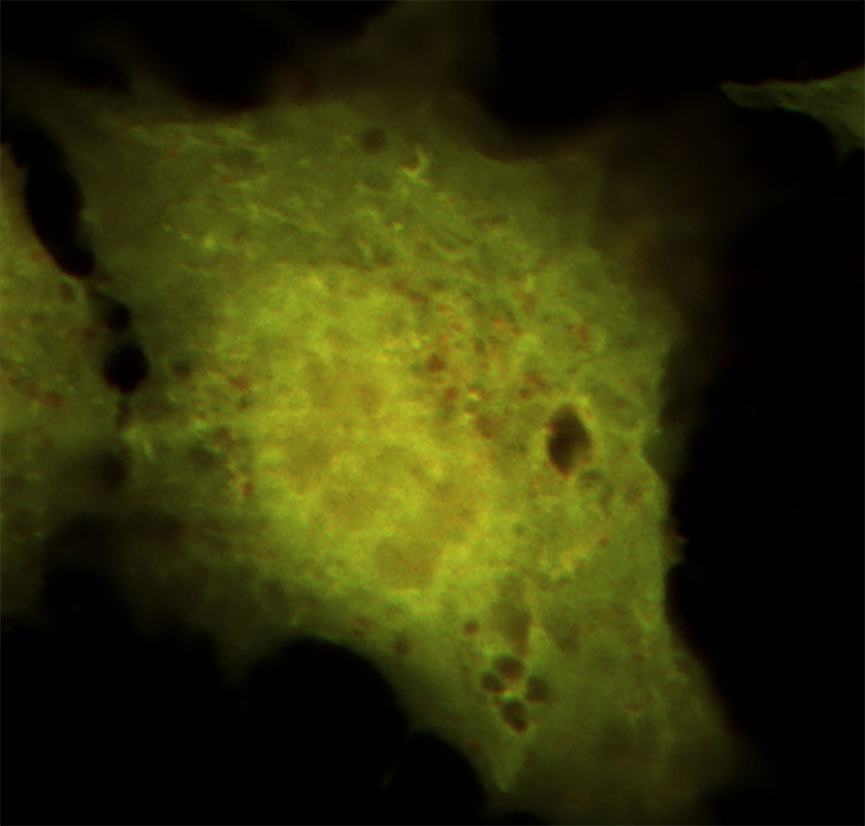

Supplement: Supplementary file 9 — Source data Fig. 4 [file 44318_2025_410_MOESM9_ESM.zip › Source Data For Figure 4/4D, F, J _microscopy/4F/1. WT_ctrl/Merged.tif]

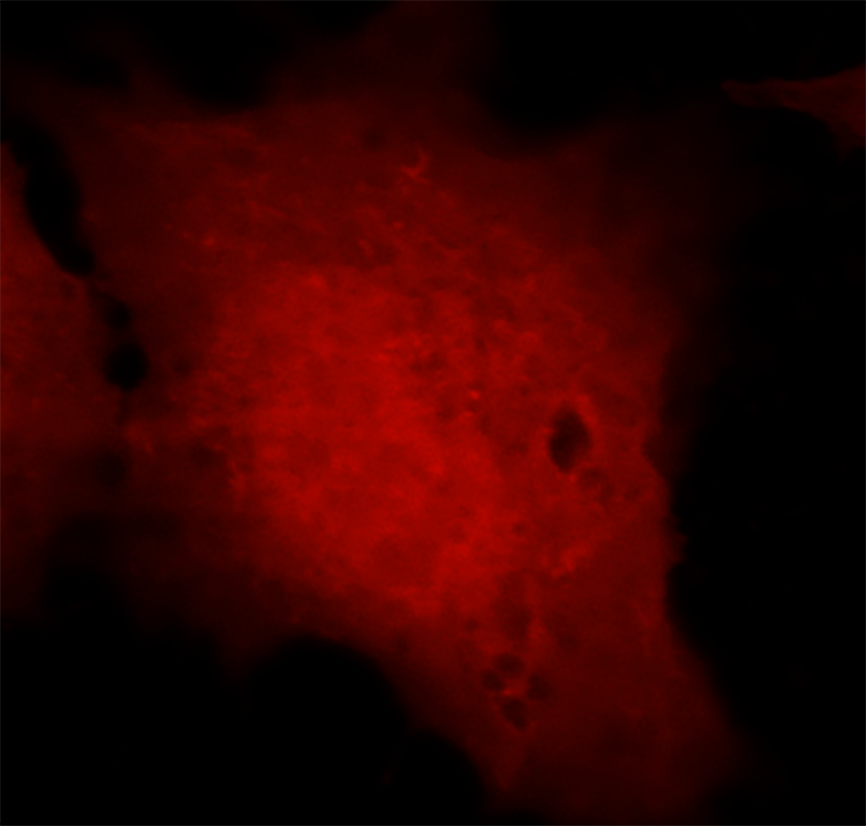

Supplement: Supplementary file 9 — Source data Fig. 4 [file 44318_2025_410_MOESM9_ESM.zip › Source Data For Figure 4/4D, F, J _microscopy/4F/1. WT_ctrl/mCherry.tif]

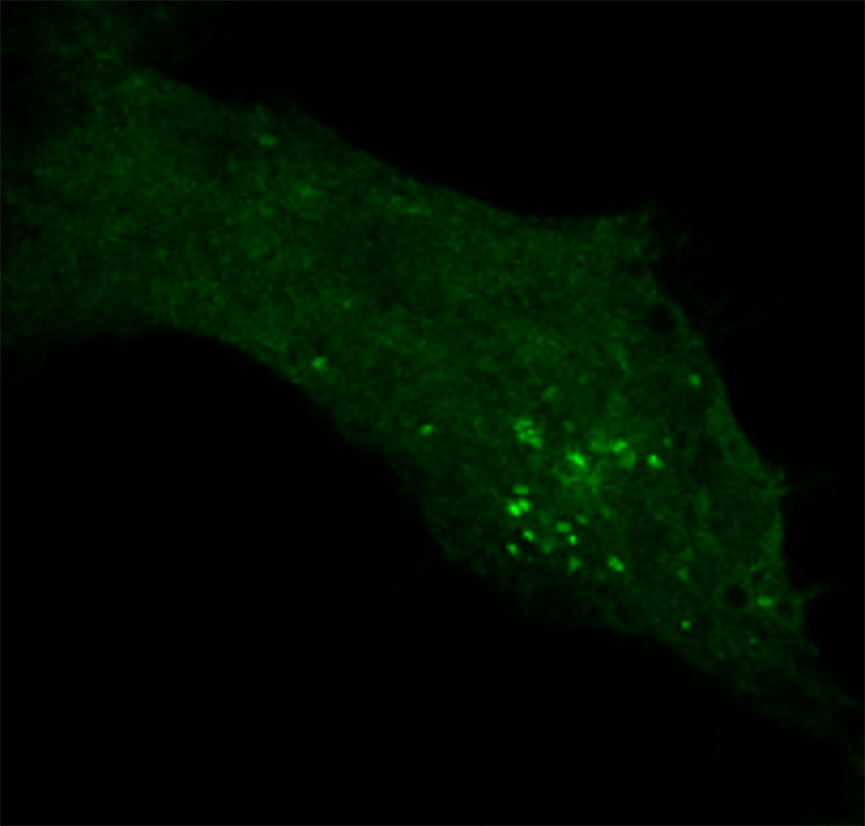

Supplement: Supplementary file 9 — Source data Fig. 4 [file 44318_2025_410_MOESM9_ESM.zip › Source Data For Figure 4/4D, F, J _microscopy/4F/2. WT_EBSS/GFP.tif]

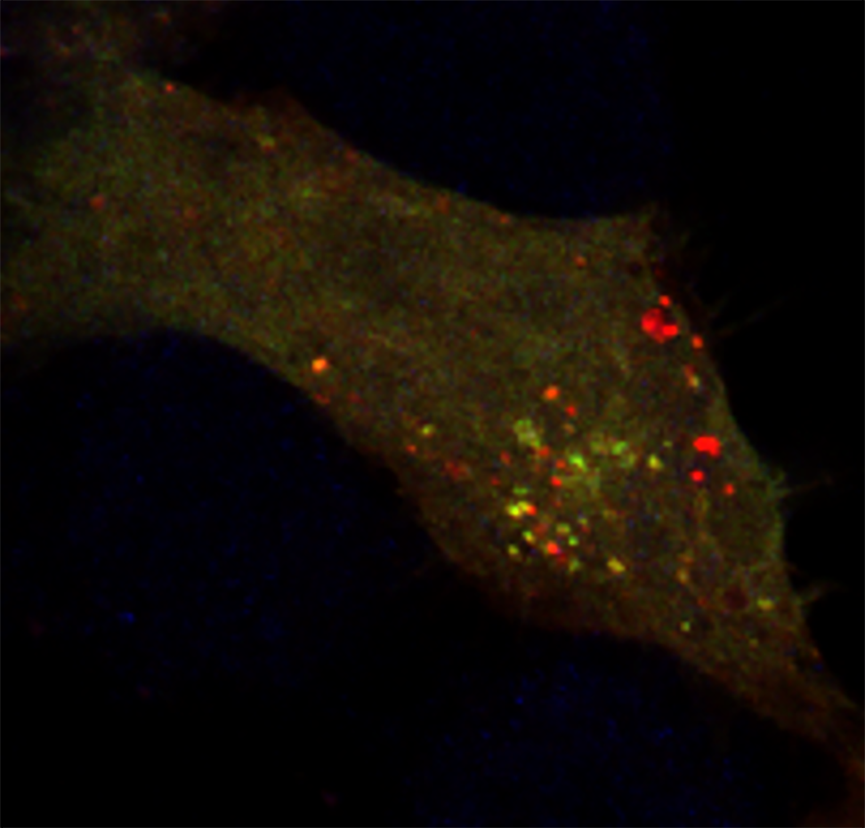

Supplement: Supplementary file 9 — Source data Fig. 4 [file 44318_2025_410_MOESM9_ESM.zip › Source Data For Figure 4/4D, F, J _microscopy/4F/2. WT_EBSS/Merged.tif]

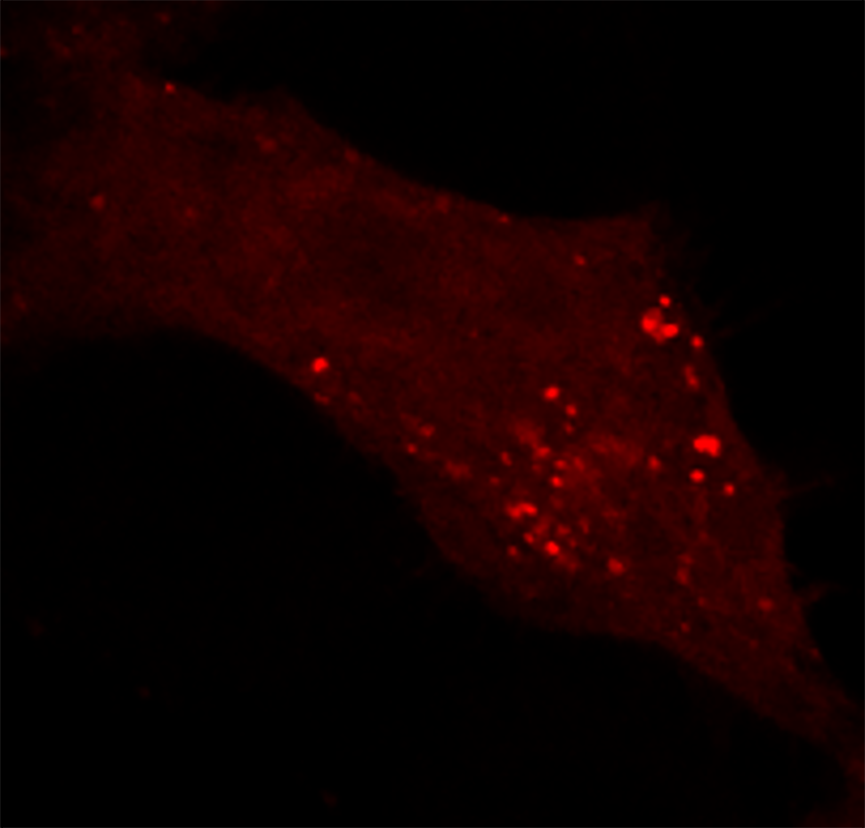

Supplement: Supplementary file 9 — Source data Fig. 4 [file 44318_2025_410_MOESM9_ESM.zip › Source Data For Figure 4/4D, F, J _microscopy/4F/2. WT_EBSS/mCherry.tif]

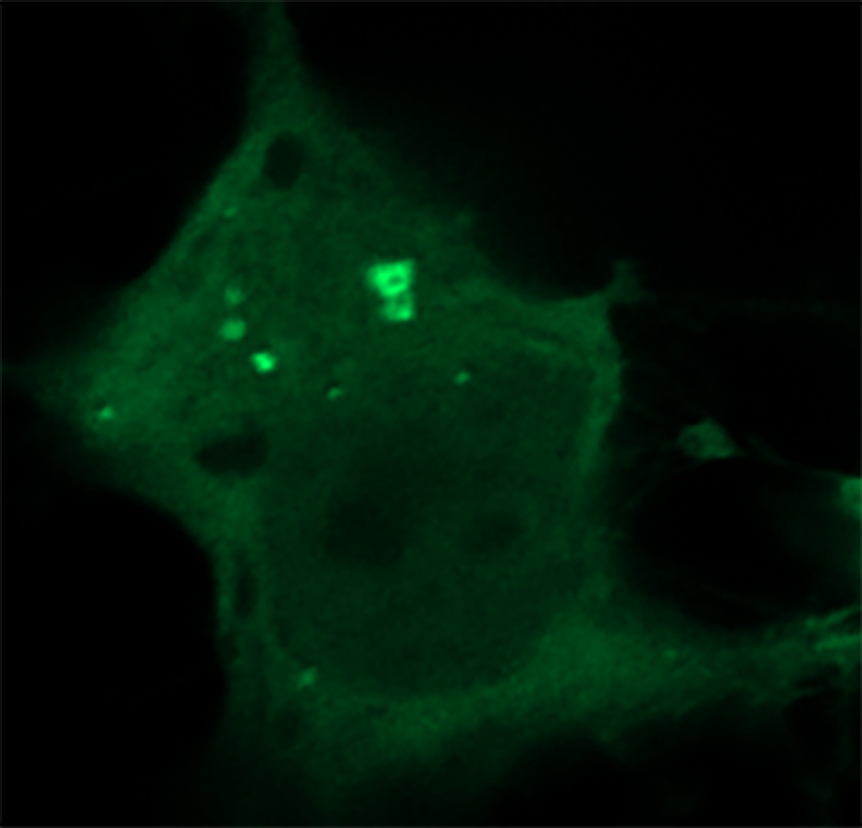

Supplement: Supplementary file 9 — Source data Fig. 4 [file 44318_2025_410_MOESM9_ESM.zip › Source Data For Figure 4/4D, F, J _microscopy/4F/3. 2A2B-DKO + HA-vec_EBSS/GFP.tif]

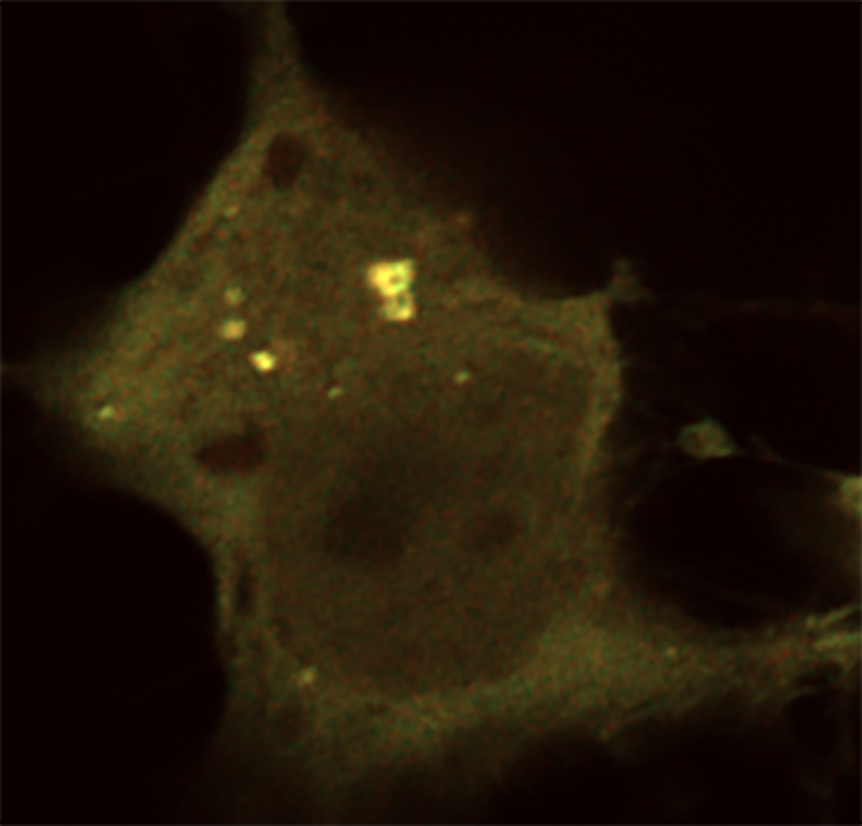

Supplement: Supplementary file 9 — Source data Fig. 4 [file 44318_2025_410_MOESM9_ESM.zip › Source Data For Figure 4/4D, F, J _microscopy/4F/3. 2A2B-DKO + HA-vec_EBSS/Merged.tif]

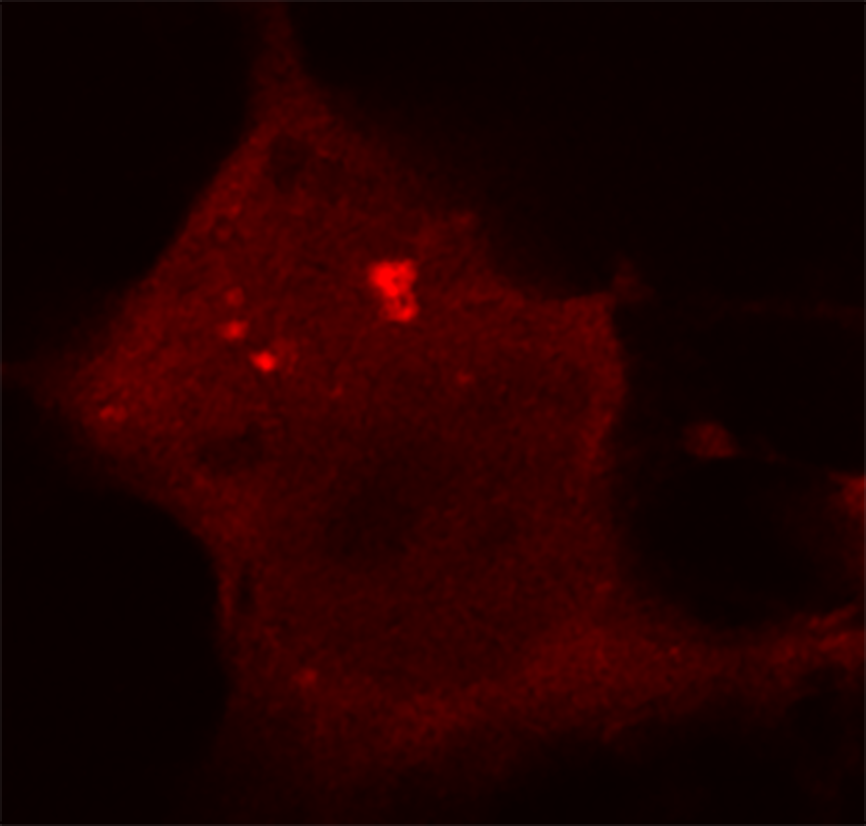

Supplement: Supplementary file 9 — Source data Fig. 4 [file 44318_2025_410_MOESM9_ESM.zip › Source Data For Figure 4/4D, F, J _microscopy/4F/3. 2A2B-DKO + HA-vec_EBSS/mCherry.tif]

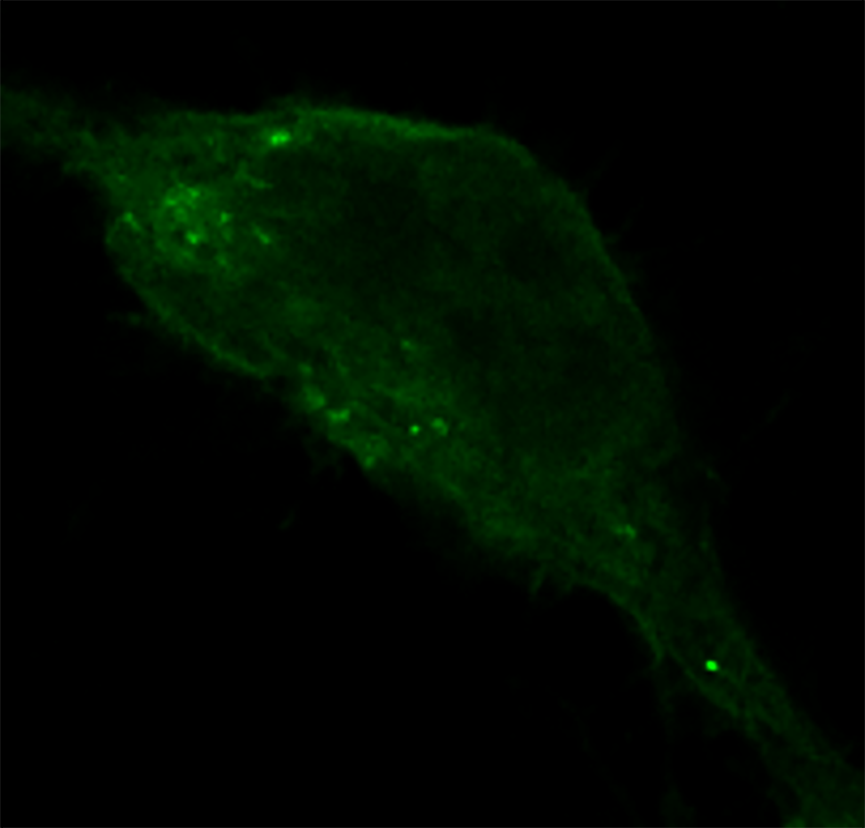

Supplement: Supplementary file 9 — Source data Fig. 4 [file 44318_2025_410_MOESM9_ESM.zip › Source Data For Figure 4/4D, F, J _microscopy/4F/4. 2A2B-DKO + HA-WT_EBSS/GFP.tif]

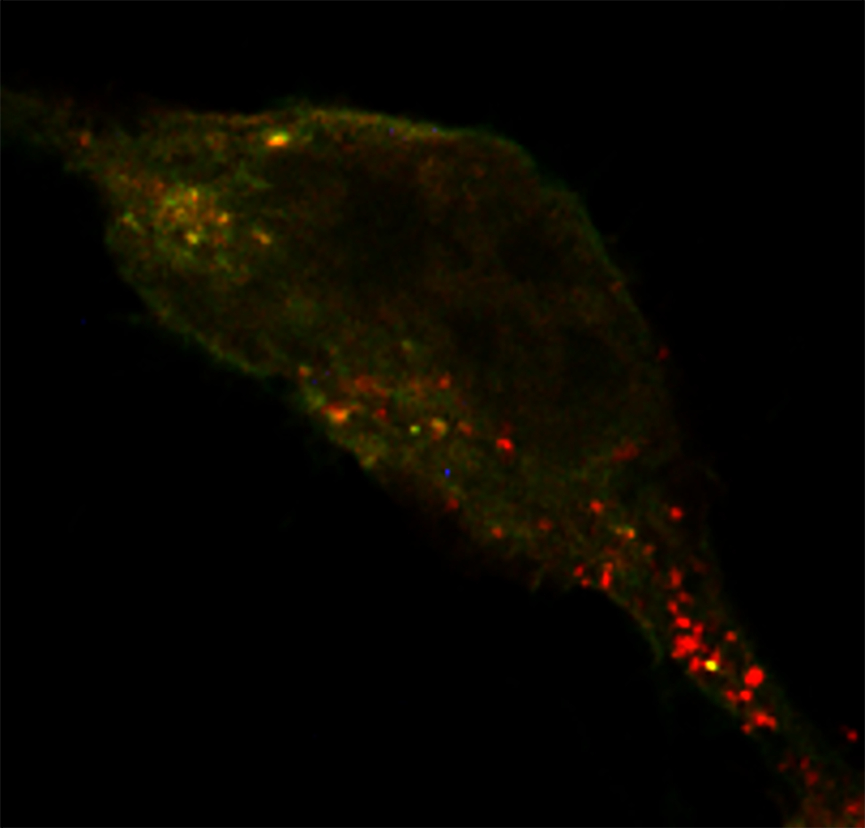

Supplement: Supplementary file 9 — Source data Fig. 4 [file 44318_2025_410_MOESM9_ESM.zip › Source Data For Figure 4/4D, F, J _microscopy/4F/4. 2A2B-DKO + HA-WT_EBSS/Merged.tif]

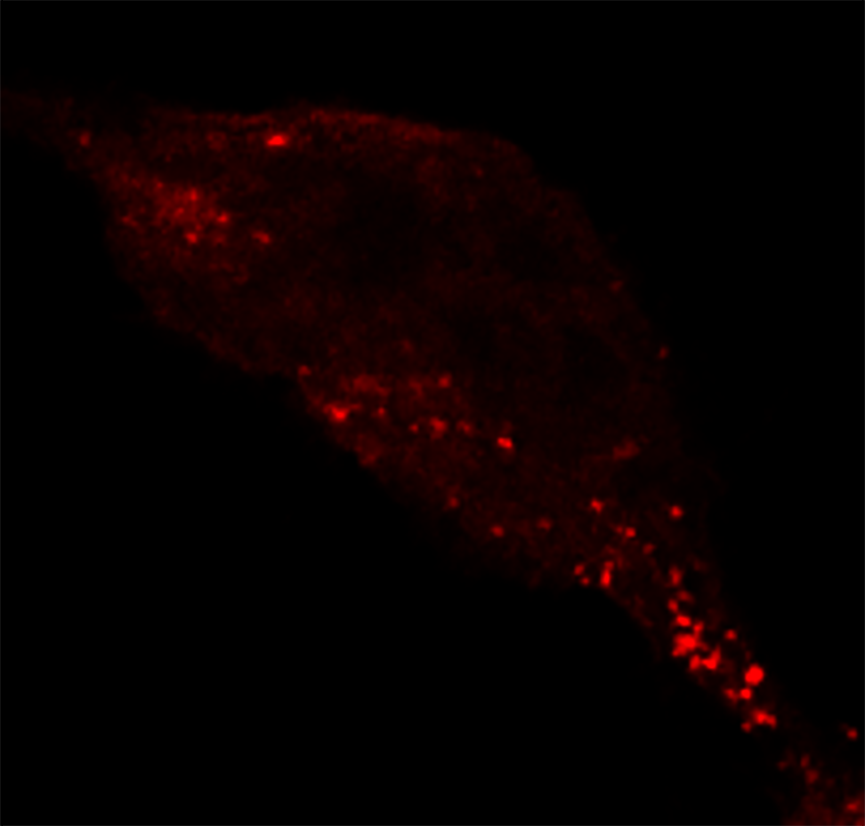

Supplement: Supplementary file 9 — Source data Fig. 4 [file 44318_2025_410_MOESM9_ESM.zip › Source Data For Figure 4/4D, F, J _microscopy/4F/4. 2A2B-DKO + HA-WT_EBSS/mCherry.tif]

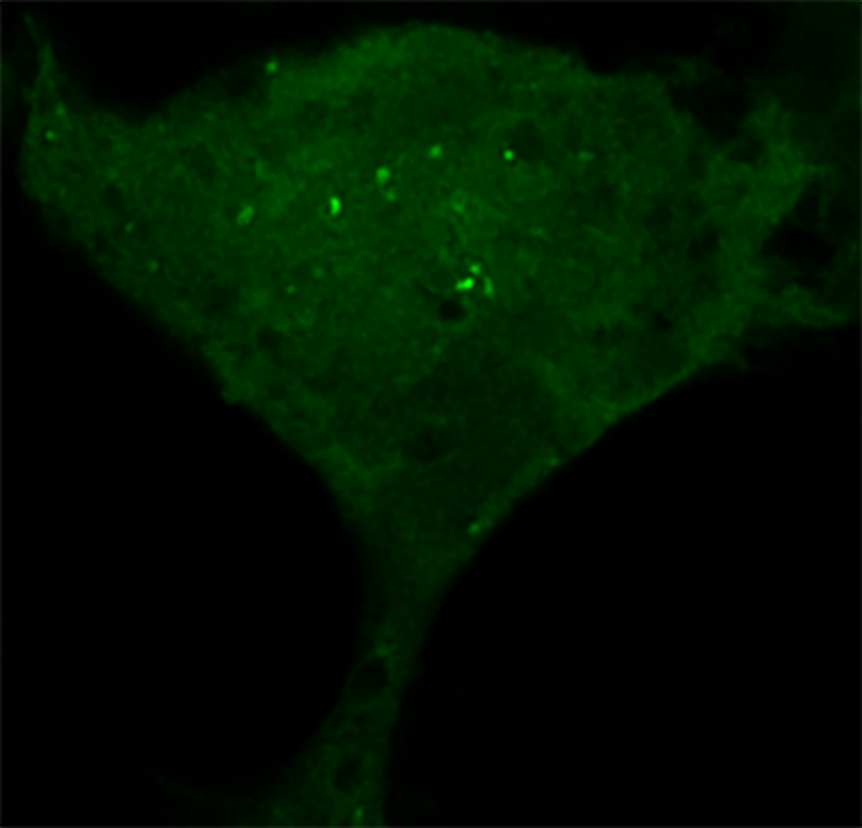

Supplement: Supplementary file 9 — Source data Fig. 4 [file 44318_2025_410_MOESM9_ESM.zip › Source Data For Figure 4/4D, F, J _microscopy/4F/5. 2A2B-DKO + HA-3CS_EBSS/GFP.tif]

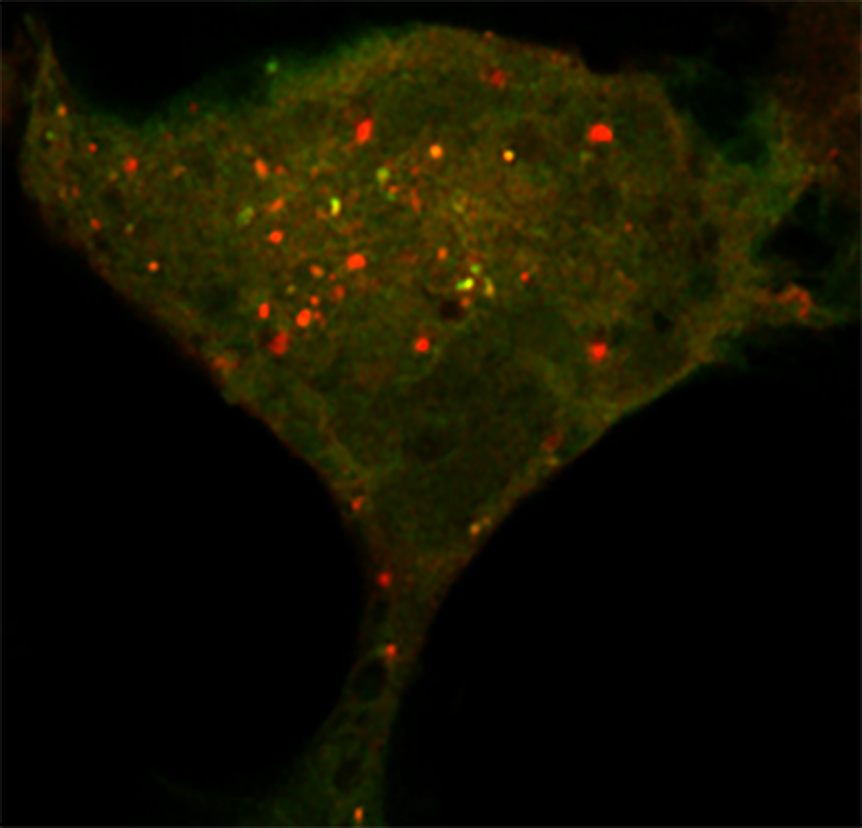

Supplement: Supplementary file 9 — Source data Fig. 4 [file 44318_2025_410_MOESM9_ESM.zip › Source Data For Figure 4/4D, F, J _microscopy/4F/5. 2A2B-DKO + HA-3CS_EBSS/Merged.tif]

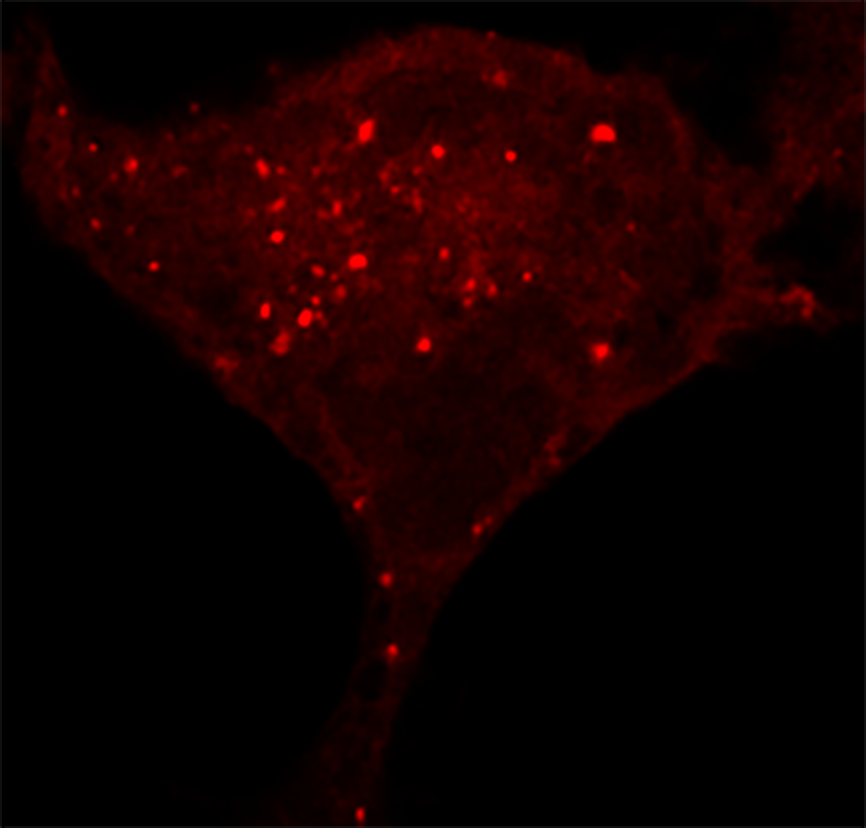

Supplement: Supplementary file 9 — Source data Fig. 4 [file 44318_2025_410_MOESM9_ESM.zip › Source Data For Figure 4/4D, F, J _microscopy/4F/5. 2A2B-DKO + HA-3CS_EBSS/mCherry.tif]

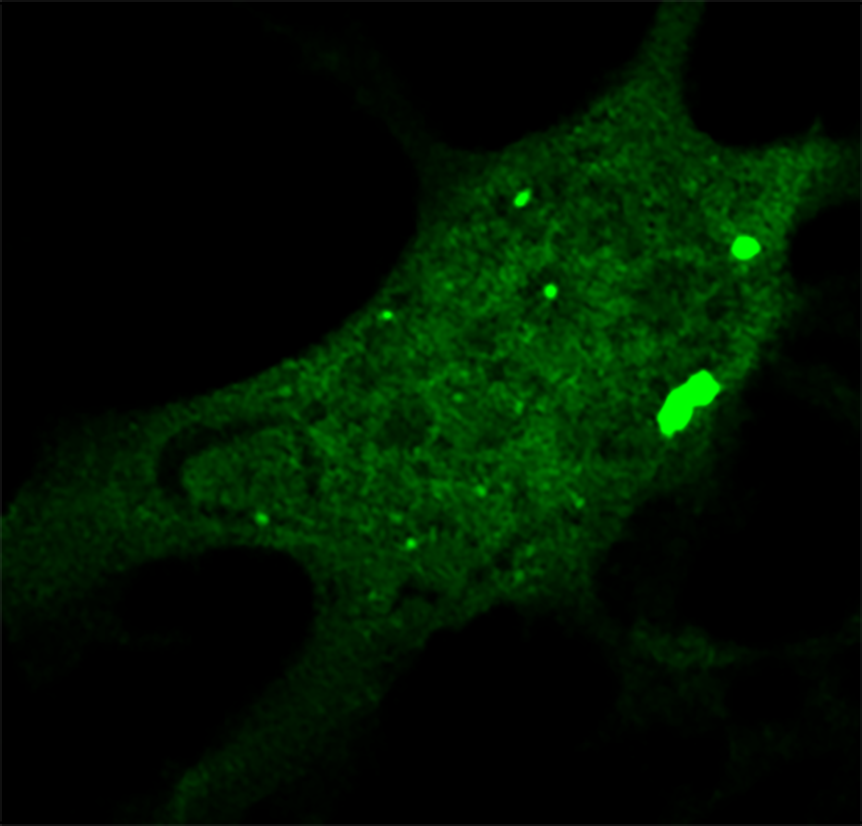

Supplement: Supplementary file 9 — Source data Fig. 4 [file 44318_2025_410_MOESM9_ESM.zip › Source Data For Figure 4/4D, F, J _microscopy/4F/6. 2A2B-DKO + HA-WT + Myc-Z11_EBSS/GFP.tif]

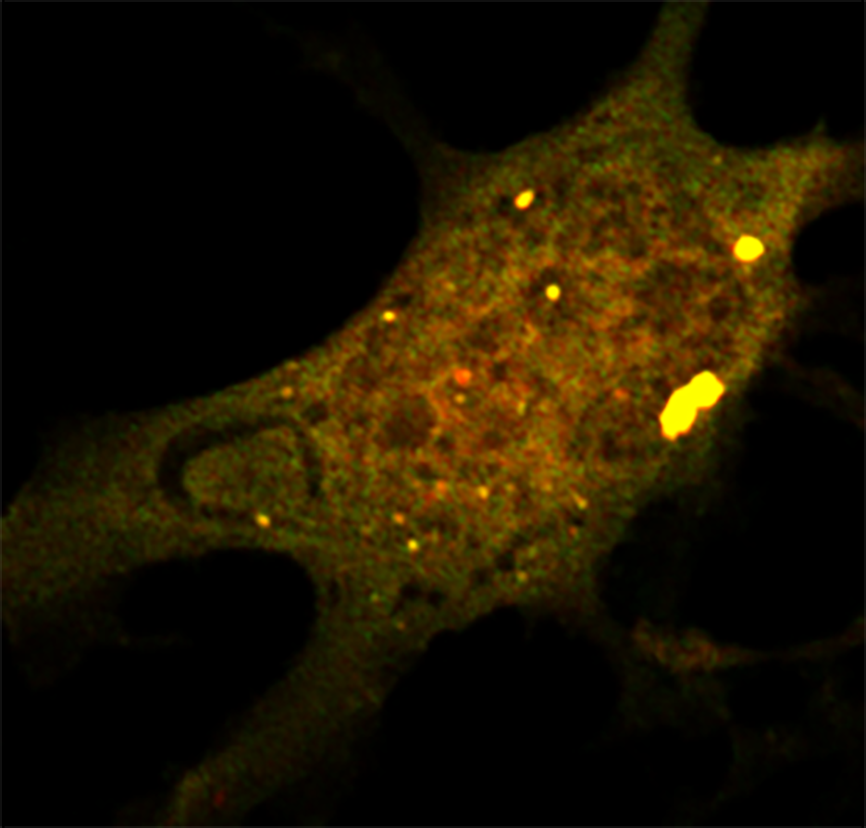

Supplement: Supplementary file 9 — Source data Fig. 4 [file 44318_2025_410_MOESM9_ESM.zip › Source Data For Figure 4/4D, F, J _microscopy/4F/6. 2A2B-DKO + HA-WT + Myc-Z11_EBSS/Merged.tif]

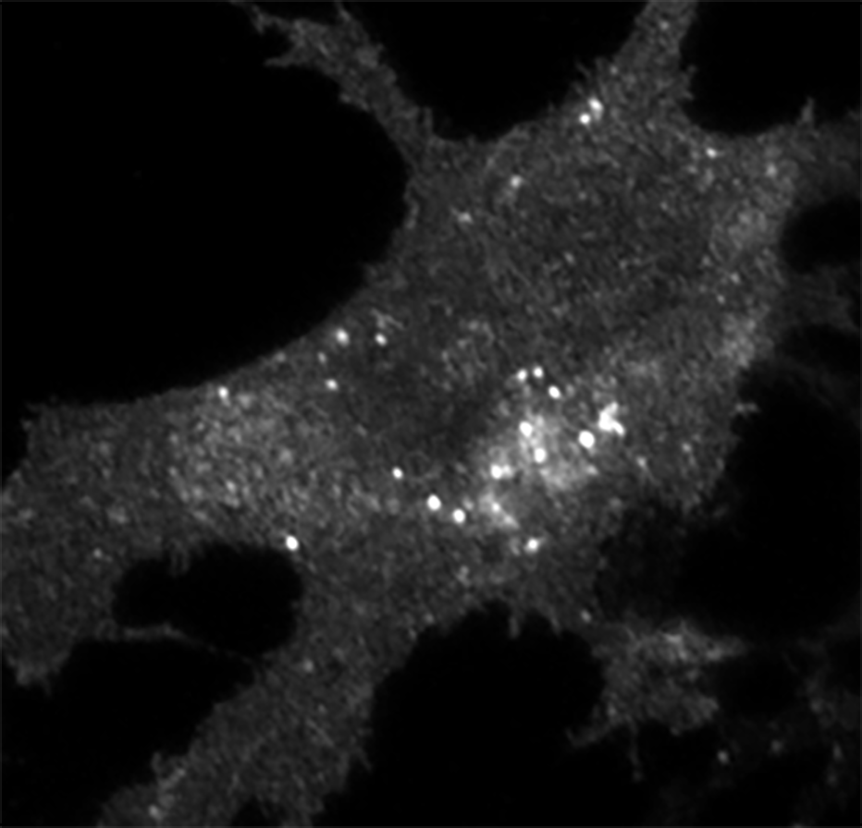

Supplement: Supplementary file 9 — Source data Fig. 4 [file 44318_2025_410_MOESM9_ESM.zip › Source Data For Figure 4/4D, F, J _microscopy/4F/6. 2A2B-DKO + HA-WT + Myc-Z11_EBSS/Myc-Z11.tif]

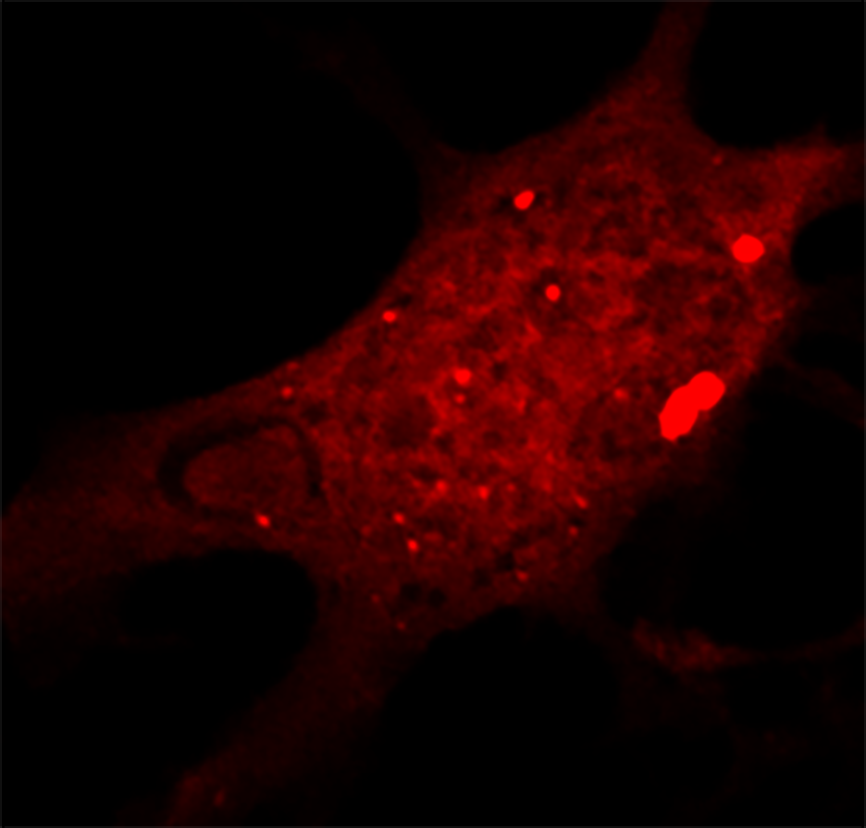

Supplement: Supplementary file 9 — Source data Fig. 4 [file 44318_2025_410_MOESM9_ESM.zip › Source Data For Figure 4/4D, F, J _microscopy/4F/6. 2A2B-DKO + HA-WT + Myc-Z11_EBSS/mCherry.tif]

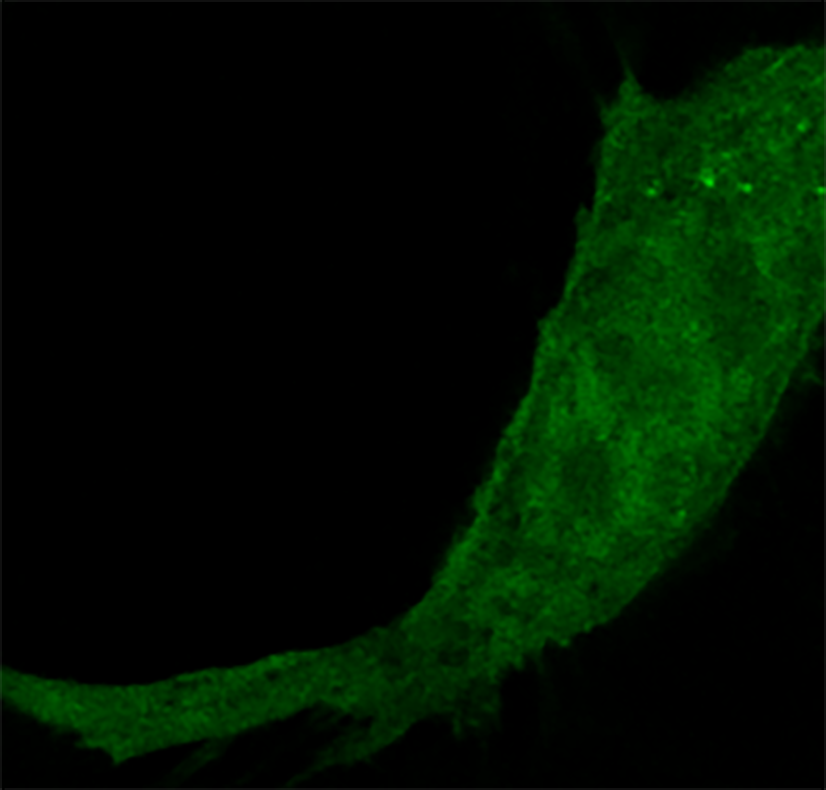

Supplement: Supplementary file 9 — Source data Fig. 4 [file 44318_2025_410_MOESM9_ESM.zip › Source Data For Figure 4/4D, F, J _microscopy/4F/7. 2A2B-DKO + HA-3CS + Myc-Z11_EBSS/GFP.tif]

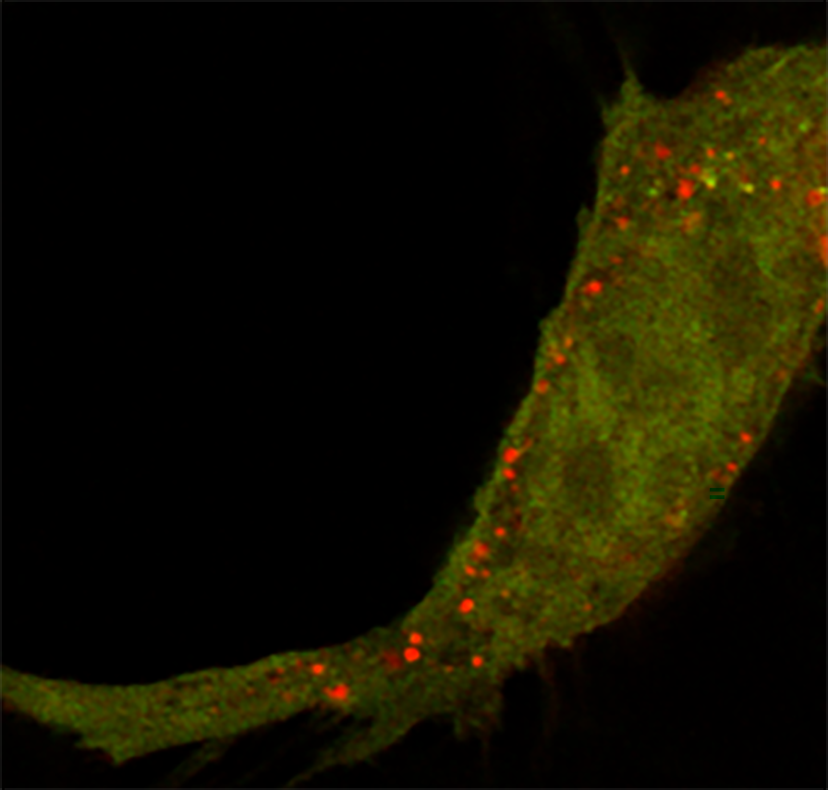

Supplement: Supplementary file 9 — Source data Fig. 4 [file 44318_2025_410_MOESM9_ESM.zip › Source Data For Figure 4/4D, F, J _microscopy/4F/7. 2A2B-DKO + HA-3CS + Myc-Z11_EBSS/Merged.tif]

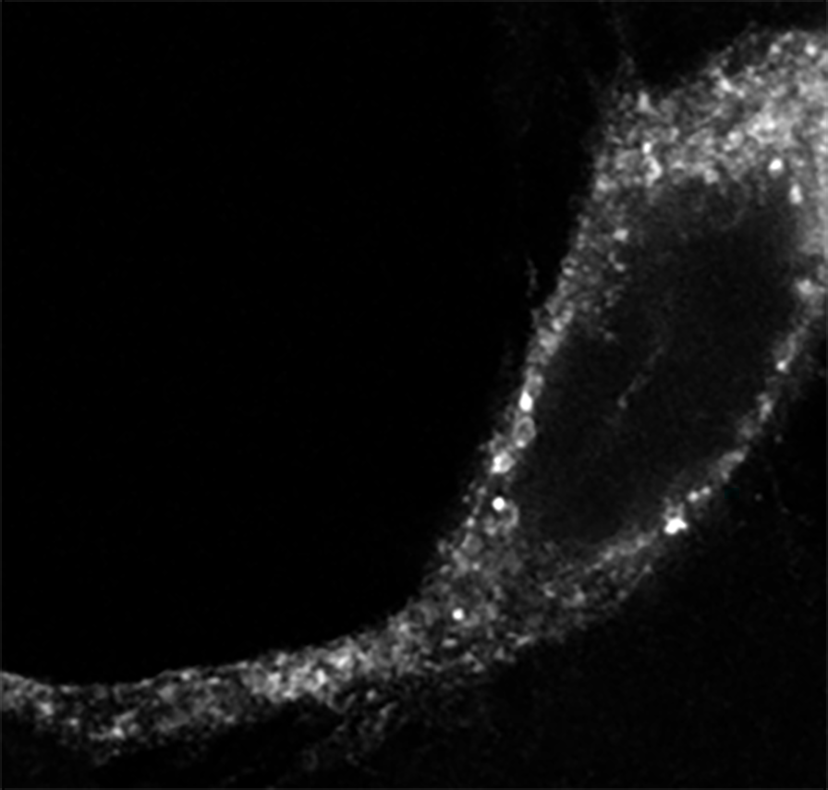

Supplement: Supplementary file 9 — Source data Fig. 4 [file 44318_2025_410_MOESM9_ESM.zip › Source Data For Figure 4/4D, F, J _microscopy/4F/7. 2A2B-DKO + HA-3CS + Myc-Z11_EBSS/Myc-Z11.tif]

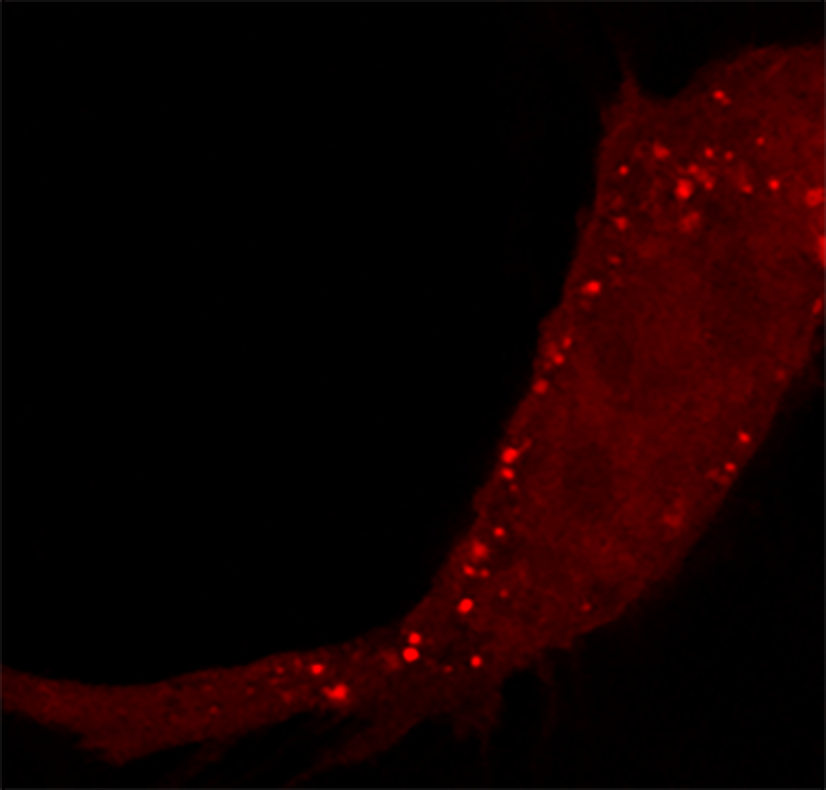

Supplement: Supplementary file 9 — Source data Fig. 4 [file 44318_2025_410_MOESM9_ESM.zip › Source Data For Figure 4/4D, F, J _microscopy/4F/7. 2A2B-DKO + HA-3CS + Myc-Z11_EBSS/mCherry.tif]

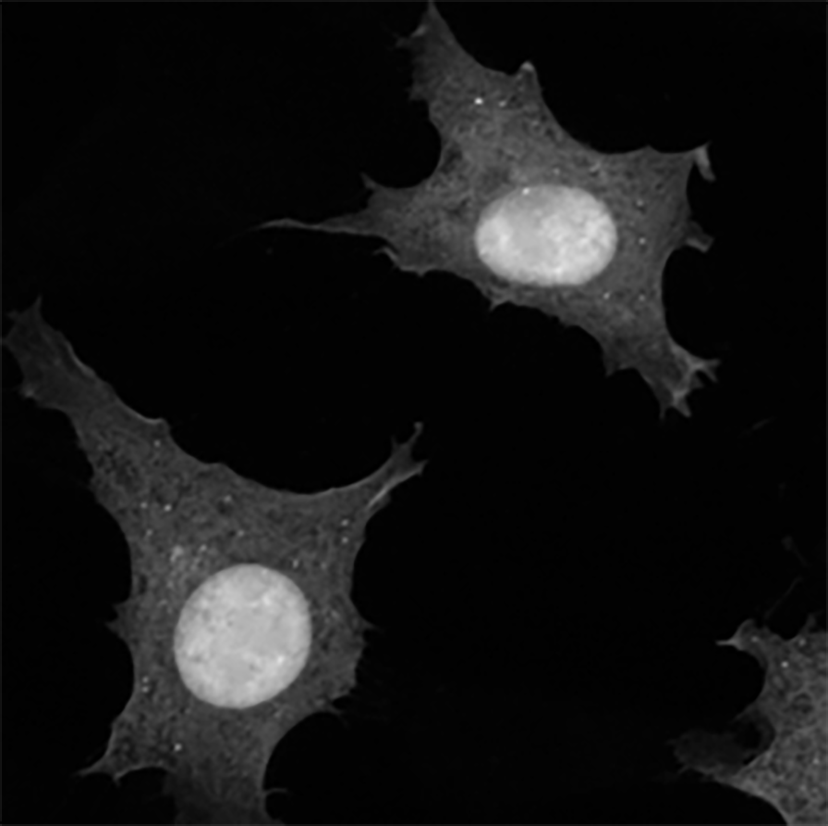

Supplement: Supplementary file 9 — Source data Fig. 4 [file 44318_2025_410_MOESM9_ESM.zip › Source Data For Figure 4/4D, F, J _microscopy/4J/1. WT_ctrl.tif]

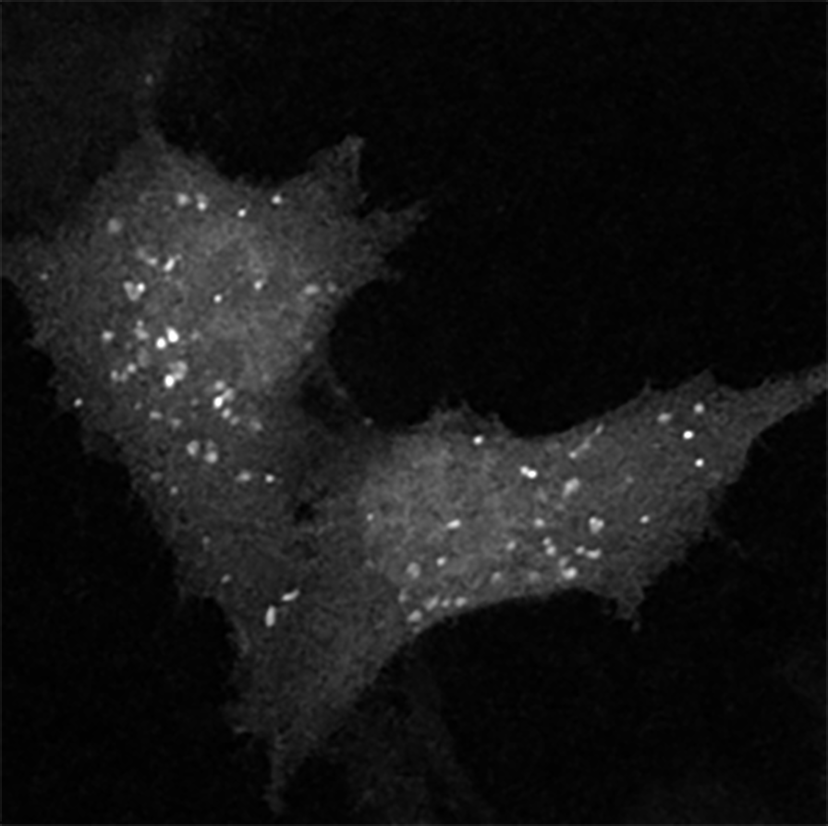

Supplement: Supplementary file 9 — Source data Fig. 4 [file 44318_2025_410_MOESM9_ESM.zip › Source Data For Figure 4/4D, F, J _microscopy/4J/2. WT_EBSS.tif]

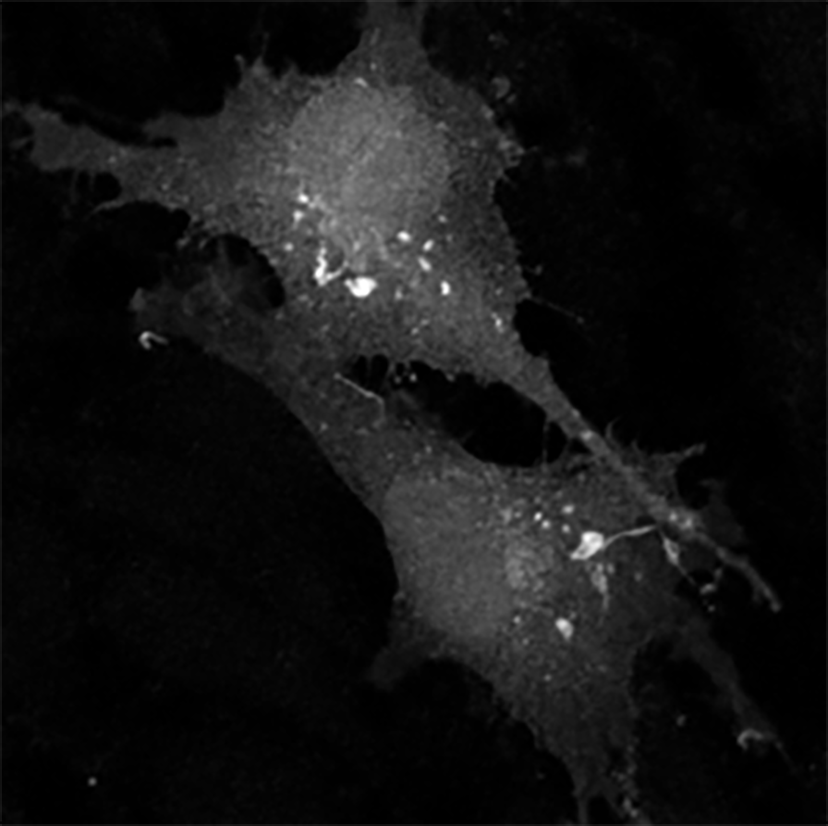

Supplement: Supplementary file 9 — Source data Fig. 4 [file 44318_2025_410_MOESM9_ESM.zip › Source Data For Figure 4/4D, F, J _microscopy/4J/3. WT + HA-Z11_EBSS.tif]

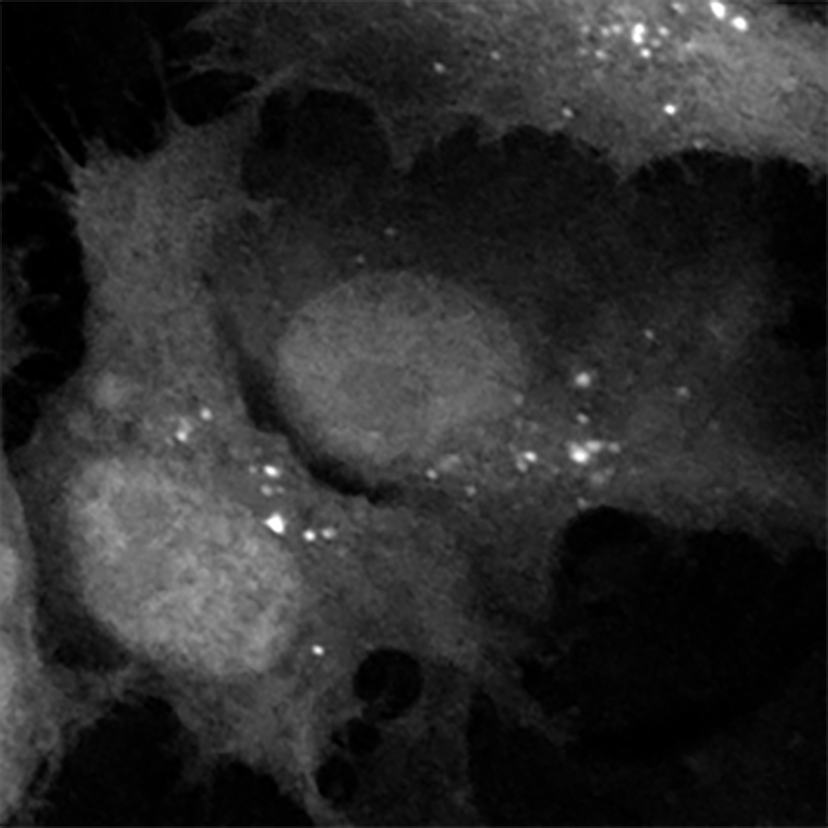

Supplement: Supplementary file 9 — Source data Fig. 4 [file 44318_2025_410_MOESM9_ESM.zip › Source Data For Figure 4/4D, F, J _microscopy/4J/4. APT1-KD + HA-vec_EBSS.tif]

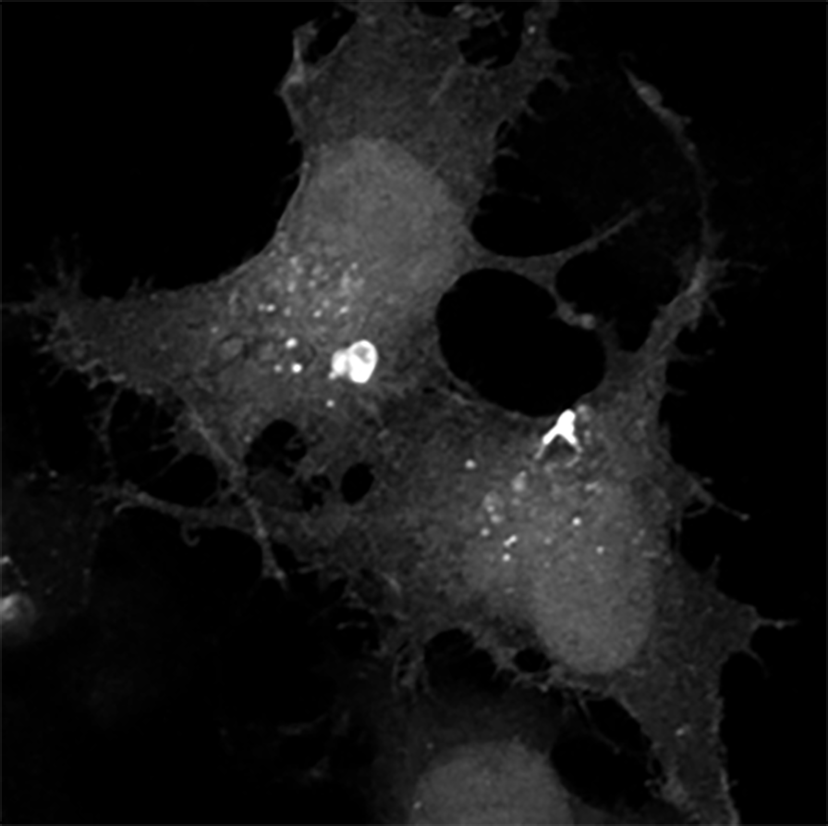

Supplement: Supplementary file 9 — Source data Fig. 4 [file 44318_2025_410_MOESM9_ESM.zip › Source Data For Figure 4/4D, F, J _microscopy/4J/5. APT1-KD + HA-Z11_EBSS.tif]

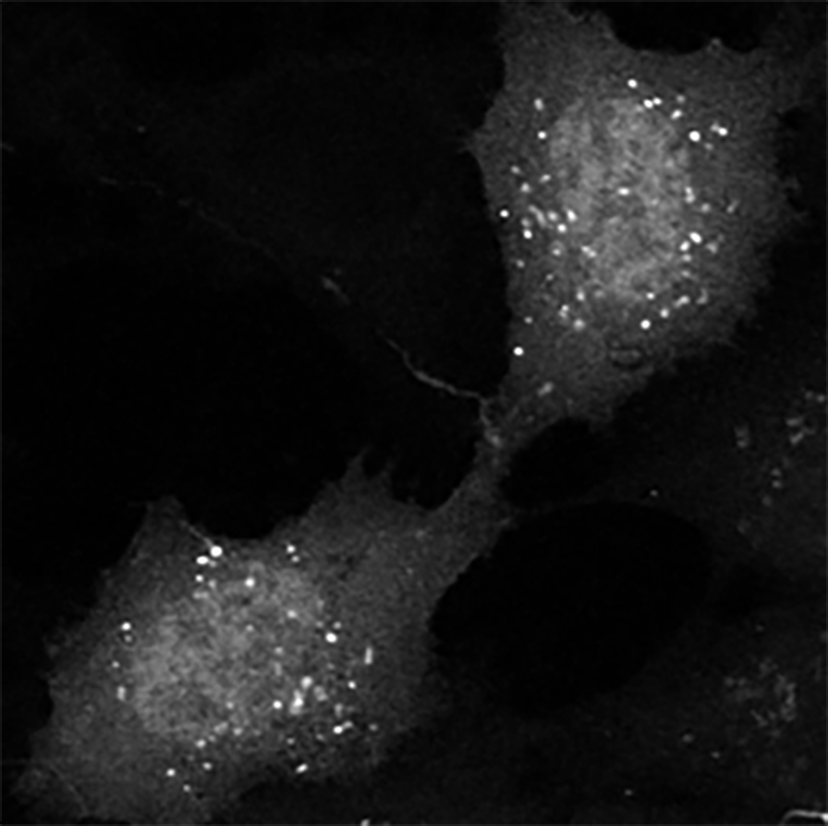

Supplement: Supplementary file 9 — Source data Fig. 4 [file 44318_2025_410_MOESM9_ESM.zip › Source Data For Figure 4/4D, F, J _microscopy/4J/6. APT1-OE + HA-vec_EBSS.tif]

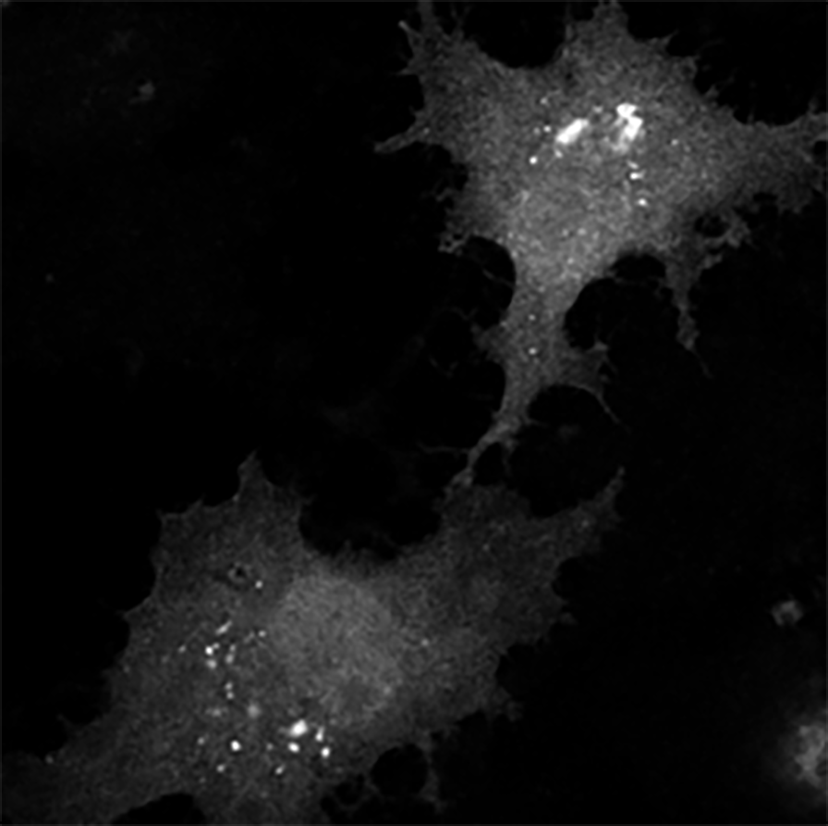

Supplement: Supplementary file 9 — Source data Fig. 4 [file 44318_2025_410_MOESM9_ESM.zip › Source Data For Figure 4/4D, F, J _microscopy/4J/7. APT1-OE + HA-Z11_EBSS.tif]

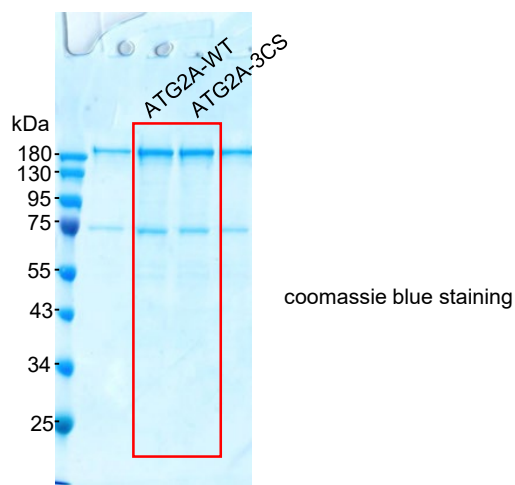

Fig 5A

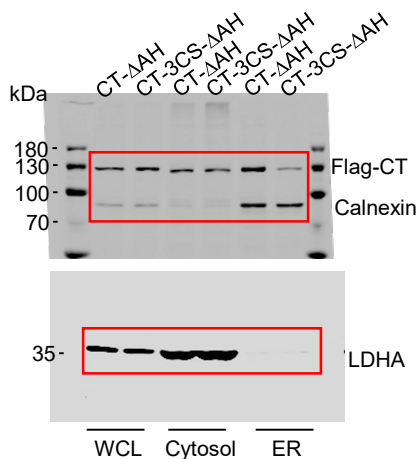

Fig 5C

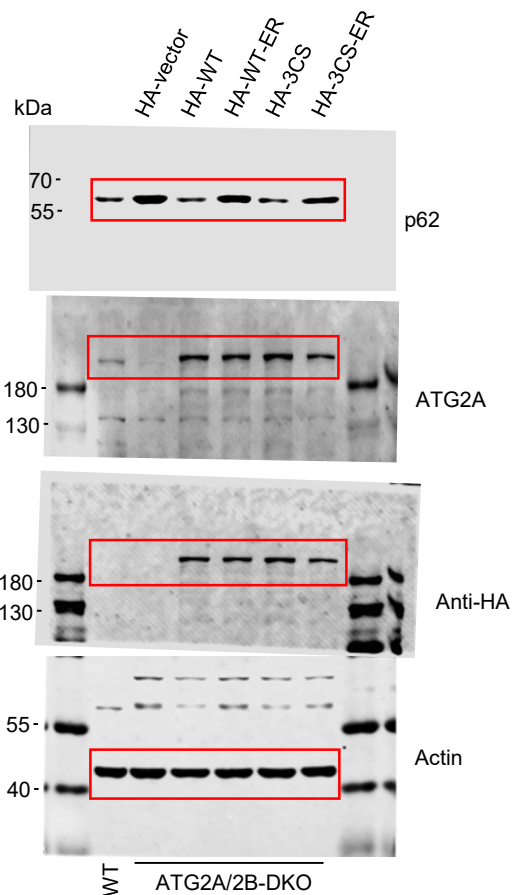

Fig 5H

Supplement: Supplementary file 10 — Source data Fig. 5 [file 44318_2025_410_MOESM10_ESM.zip › Source Data For Figure 5/5A, C, H _western blot/Figure 5.pdf]

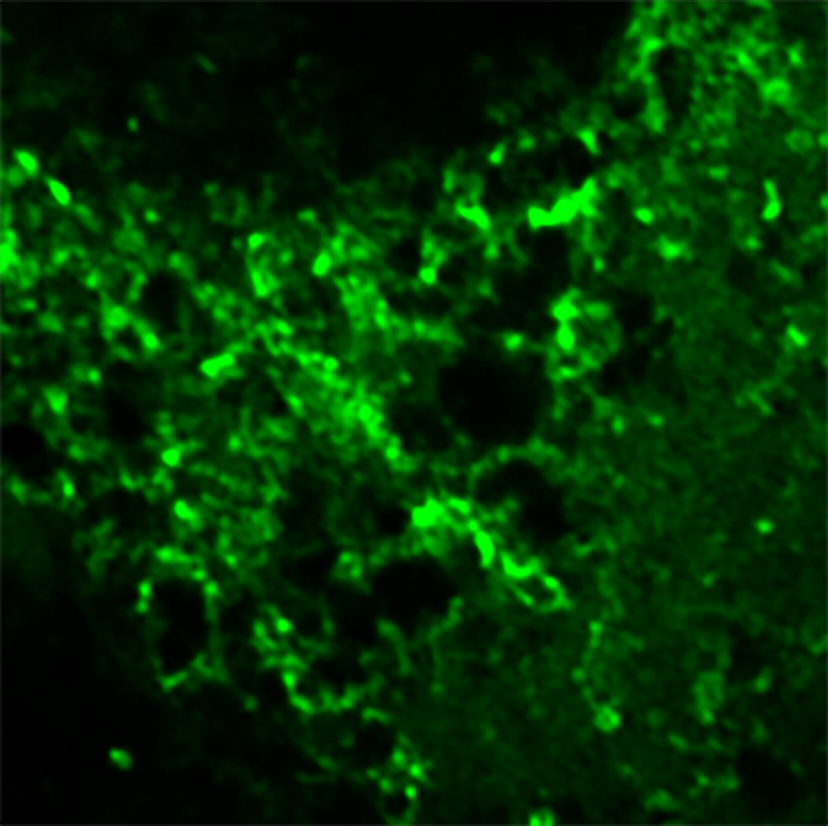

Supplement: Supplementary file 10 — Source data Fig. 5 [file 44318_2025_410_MOESM10_ESM.zip › Source Data For Figure 5/5E, F, J _microscopy/5E/1. GFP-Sec61β.tif]

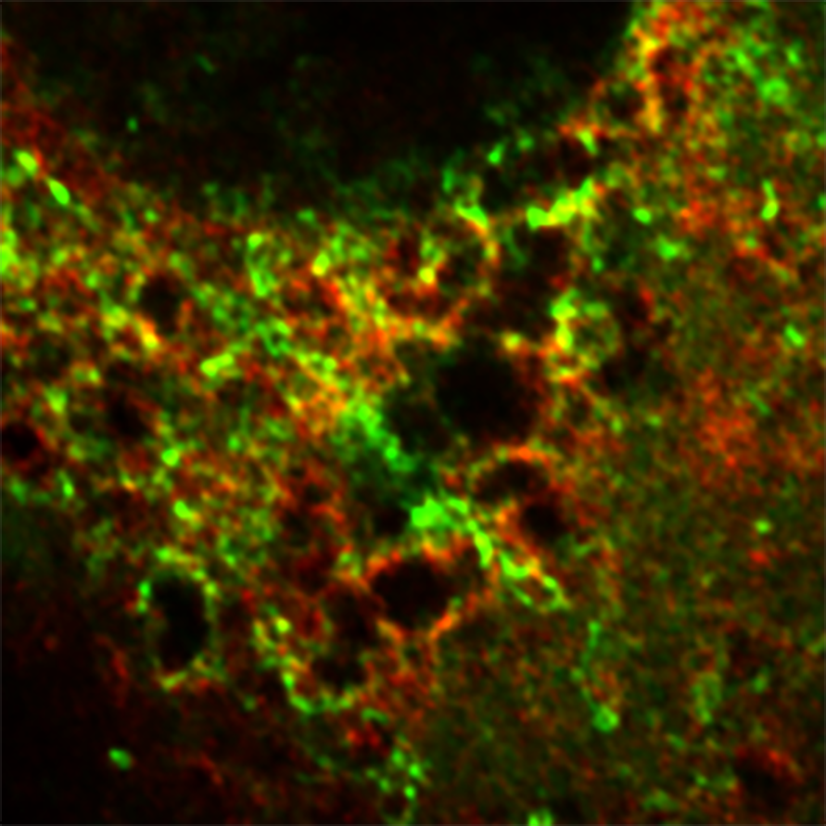

Supplement: Supplementary file 10 — Source data Fig. 5 [file 44318_2025_410_MOESM10_ESM.zip › Source Data For Figure 5/5E, F, J _microscopy/5E/1. Merged.tif]

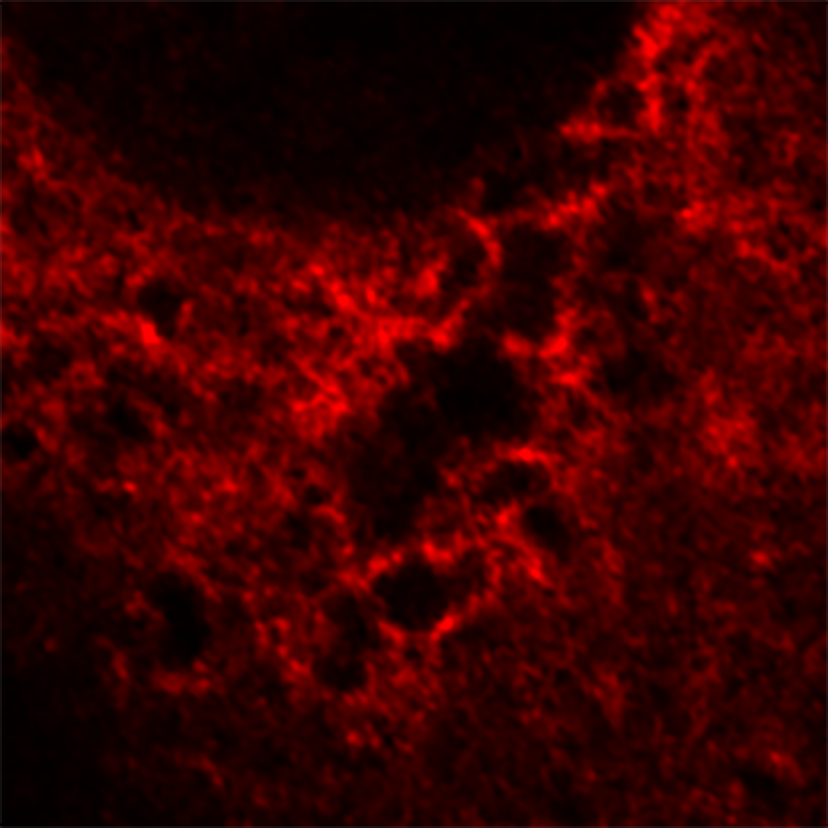

Supplement: Supplementary file 10 — Source data Fig. 5 [file 44318_2025_410_MOESM10_ESM.zip › Source Data For Figure 5/5E, F, J _microscopy/5E/1. mCherry-CT.tif]

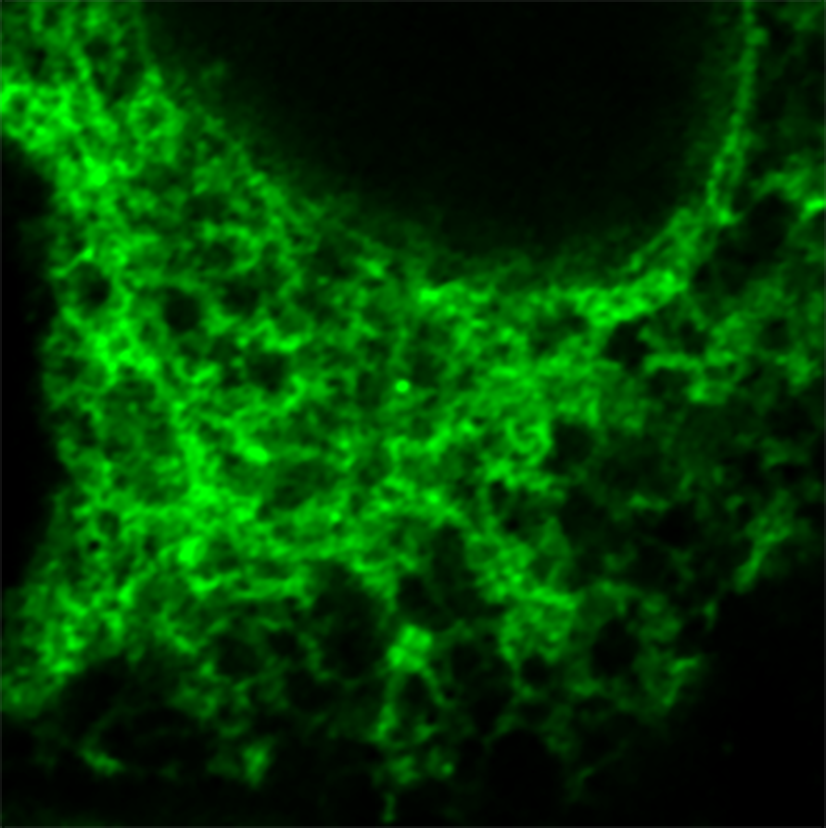

Supplement: Supplementary file 10 — Source data Fig. 5 [file 44318_2025_410_MOESM10_ESM.zip › Source Data For Figure 5/5E, F, J _microscopy/5E/2. GFP-CT-3CS.tif]

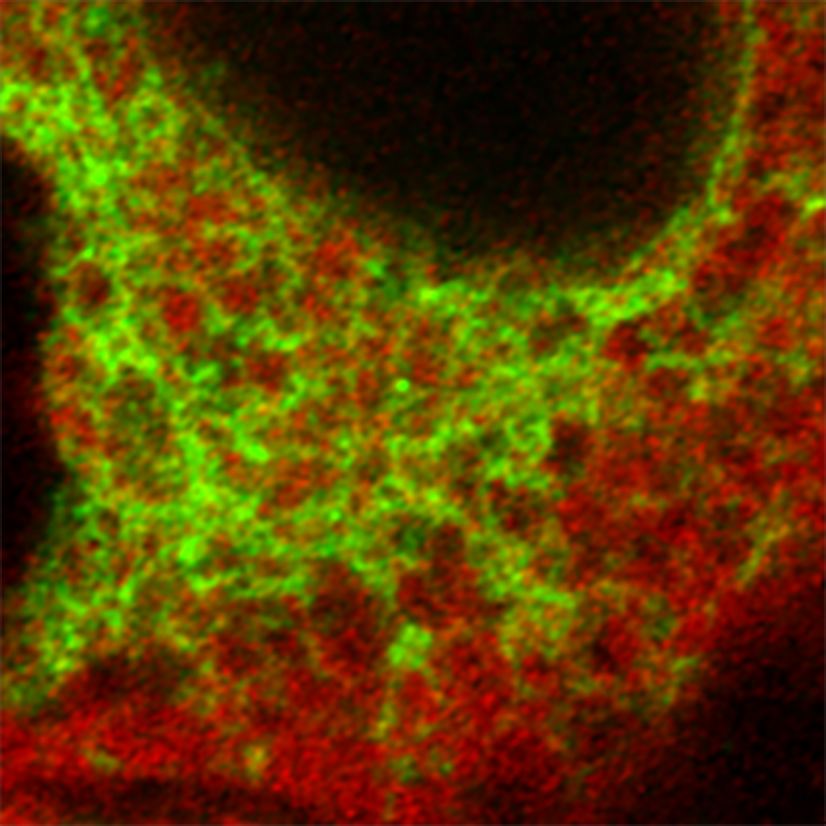

Supplement: Supplementary file 10 — Source data Fig. 5 [file 44318_2025_410_MOESM10_ESM.zip › Source Data For Figure 5/5E, F, J _microscopy/5E/2. Merged.tif]

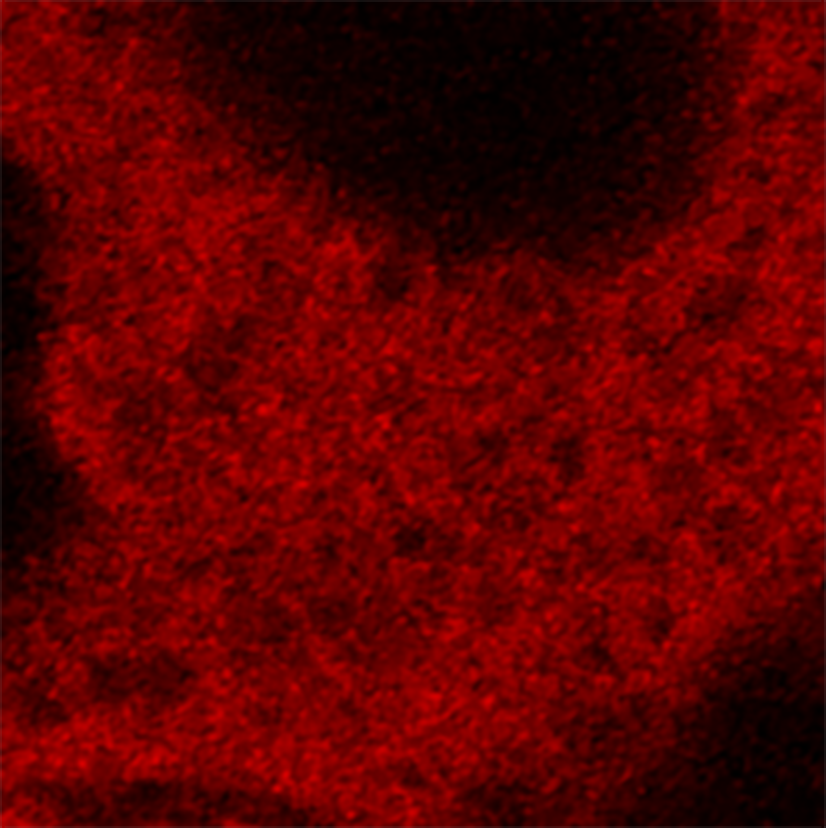

Supplement: Supplementary file 10 — Source data Fig. 5 [file 44318_2025_410_MOESM10_ESM.zip › Source Data For Figure 5/5E, F, J _microscopy/5E/2. mCherry-CT-3CS.tif]

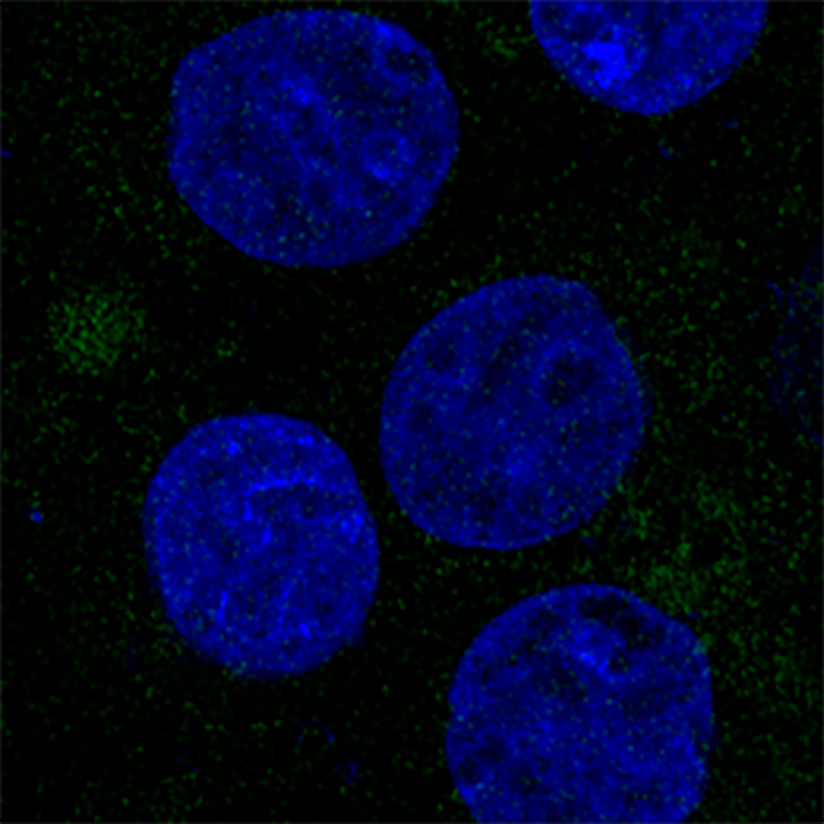

Supplement: Supplementary file 10 — Source data Fig. 5 [file 44318_2025_410_MOESM10_ESM.zip › Source Data For Figure 5/5E, F, J _microscopy/5F/1. GFP1-10 -ER.tif]

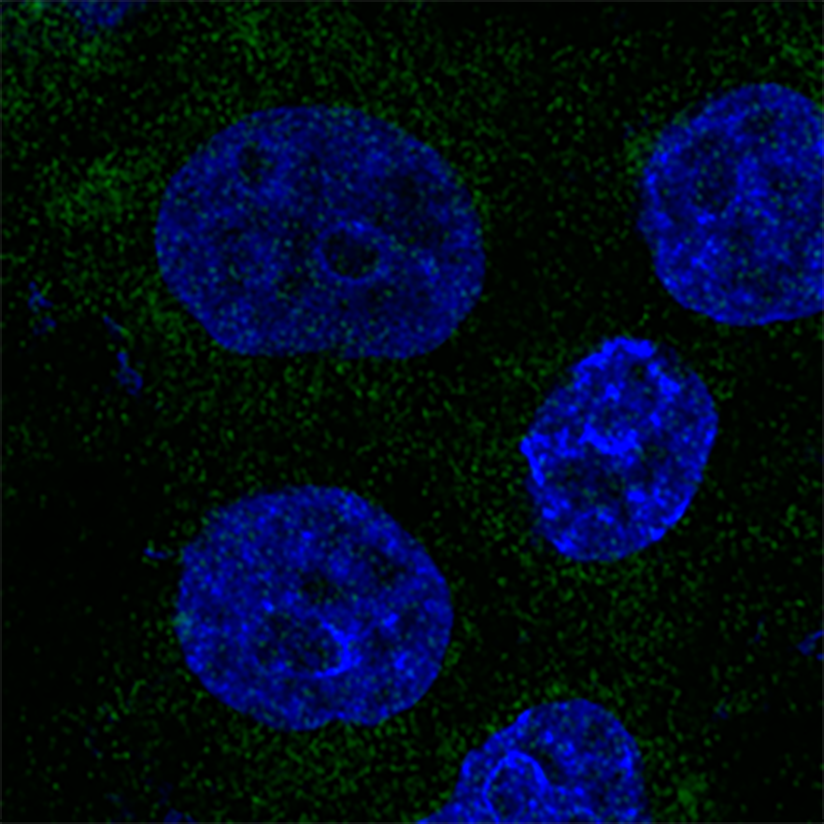

Supplement: Supplementary file 10 — Source data Fig. 5 [file 44318_2025_410_MOESM10_ESM.zip › Source Data For Figure 5/5E, F, J _microscopy/5F/2. GFP11 -CT.tif]

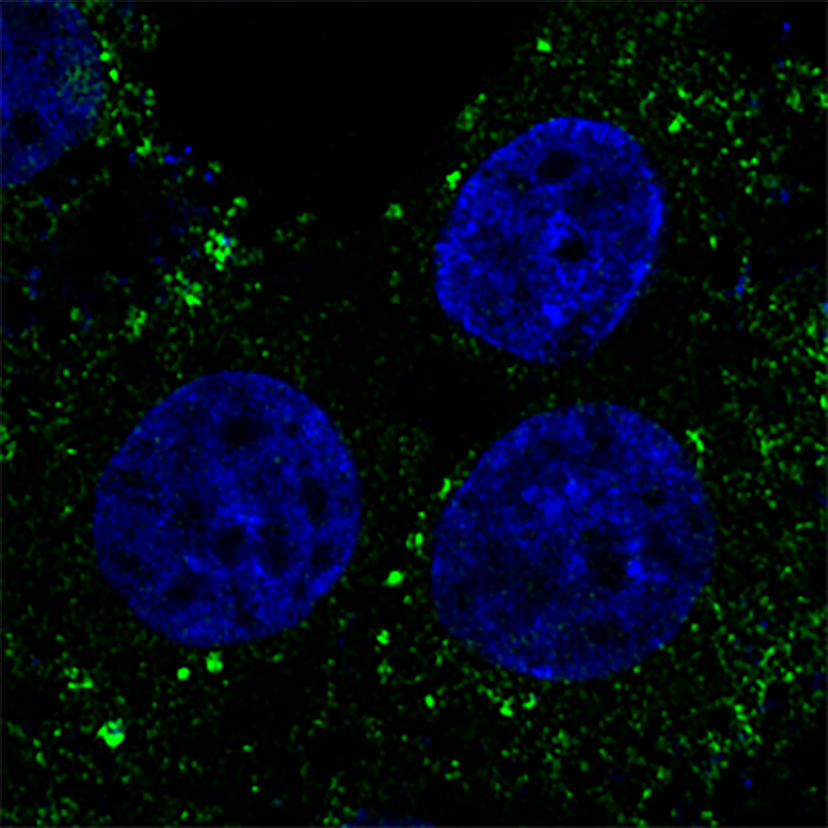

Supplement: Supplementary file 10 — Source data Fig. 5 [file 44318_2025_410_MOESM10_ESM.zip › Source Data For Figure 5/5E, F, J _microscopy/5F/3. GFP11 -CT + GFP1-10 -ER.tif]

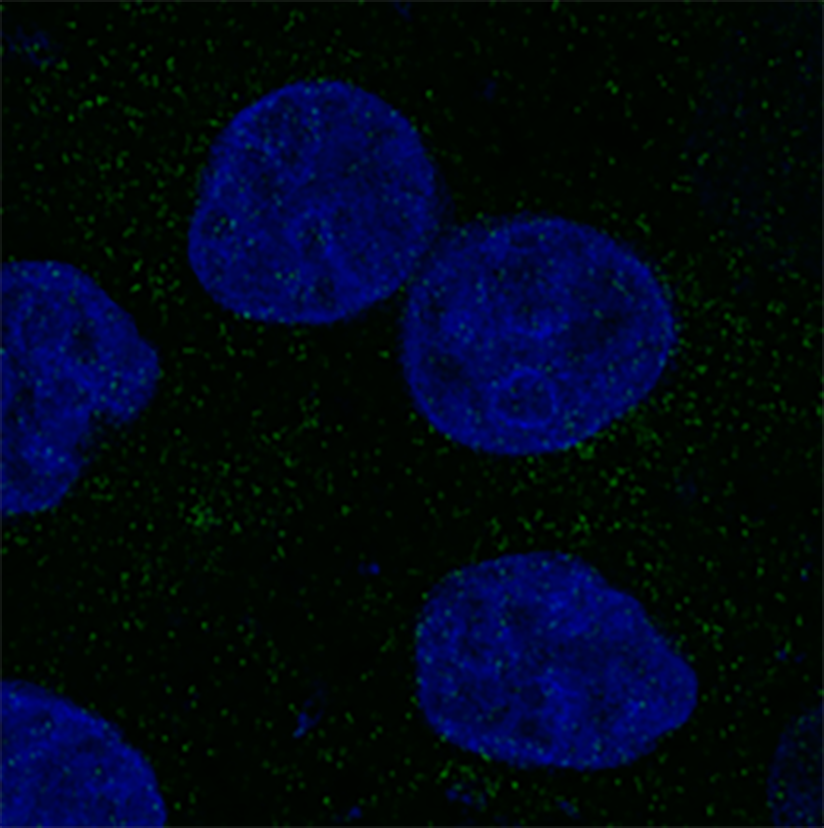

Supplement: Supplementary file 10 — Source data Fig. 5 [file 44318_2025_410_MOESM10_ESM.zip › Source Data For Figure 5/5E, F, J _microscopy/5F/4. GFP1-10 -ER.tif]

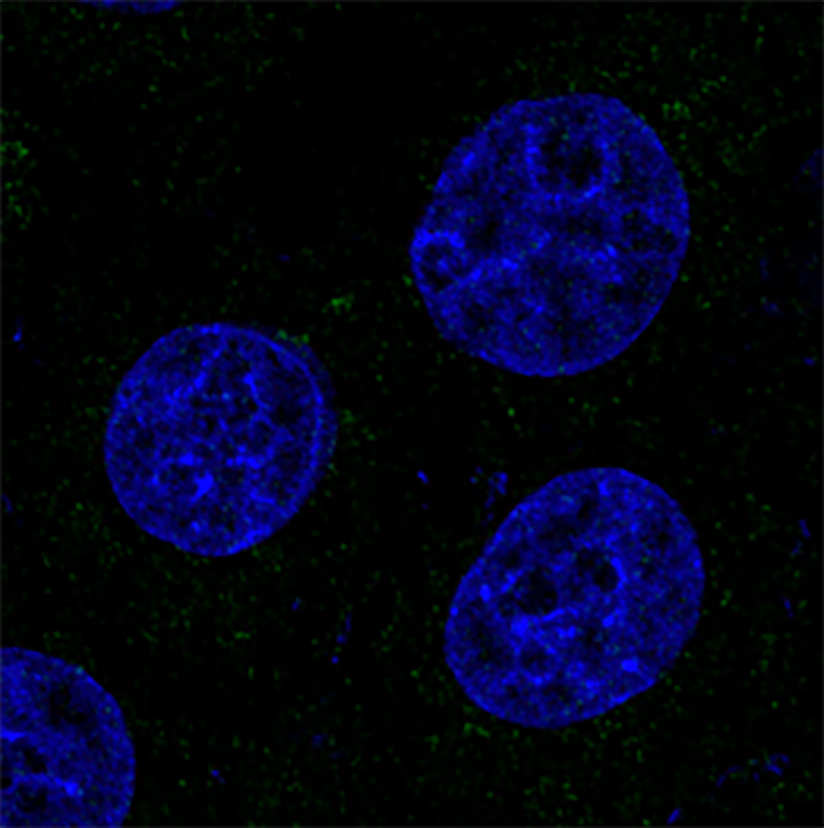

Supplement: Supplementary file 10 — Source data Fig. 5 [file 44318_2025_410_MOESM10_ESM.zip › Source Data For Figure 5/5E, F, J _microscopy/5F/5. GFP11 -CT-3CS.tif]

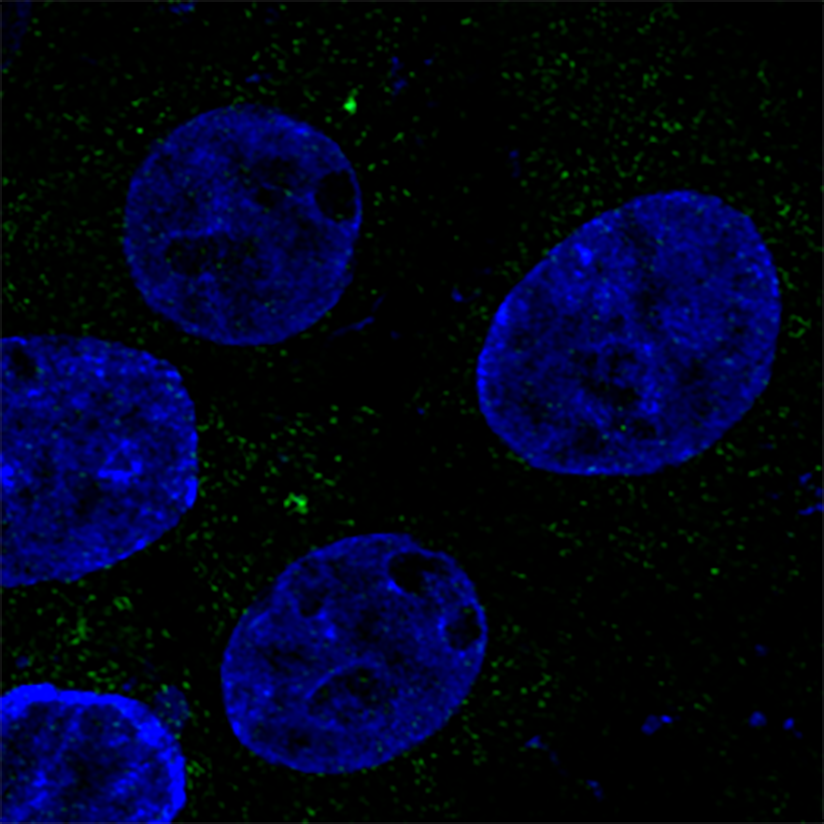

Supplement: Supplementary file 10 — Source data Fig. 5 [file 44318_2025_410_MOESM10_ESM.zip › Source Data For Figure 5/5E, F, J _microscopy/5F/6. GFP11-CT-3CS + GFP1-10 -ER.tif]

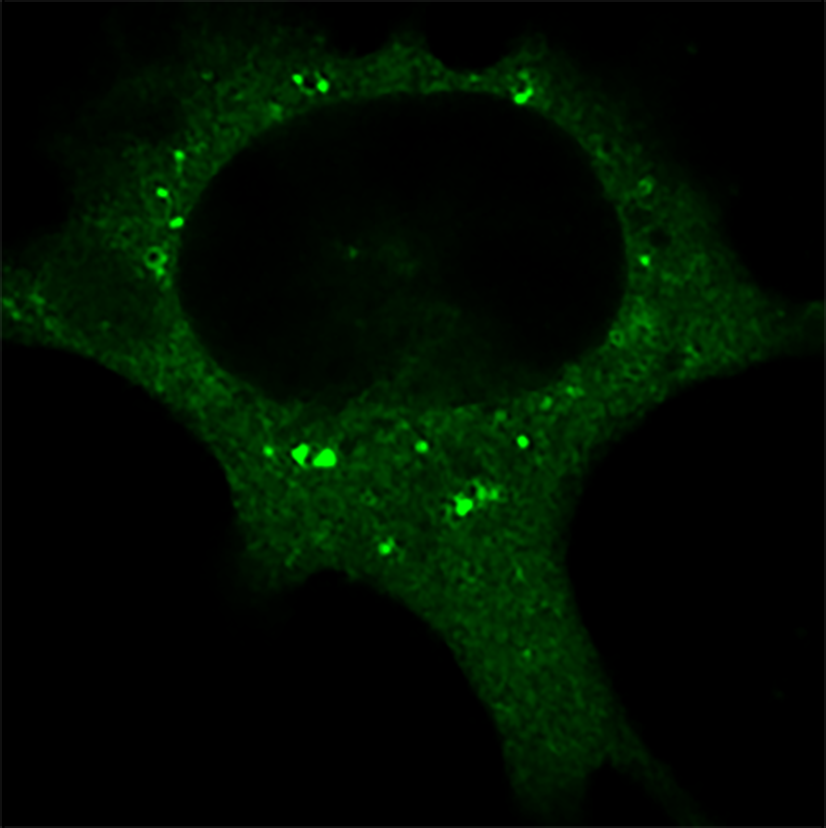

Supplement: Supplementary file 10 — Source data Fig. 5 [file 44318_2025_410_MOESM10_ESM.zip › Source Data For Figure 5/5E, F, J _microscopy/5J/1. GFP-2A.tif]

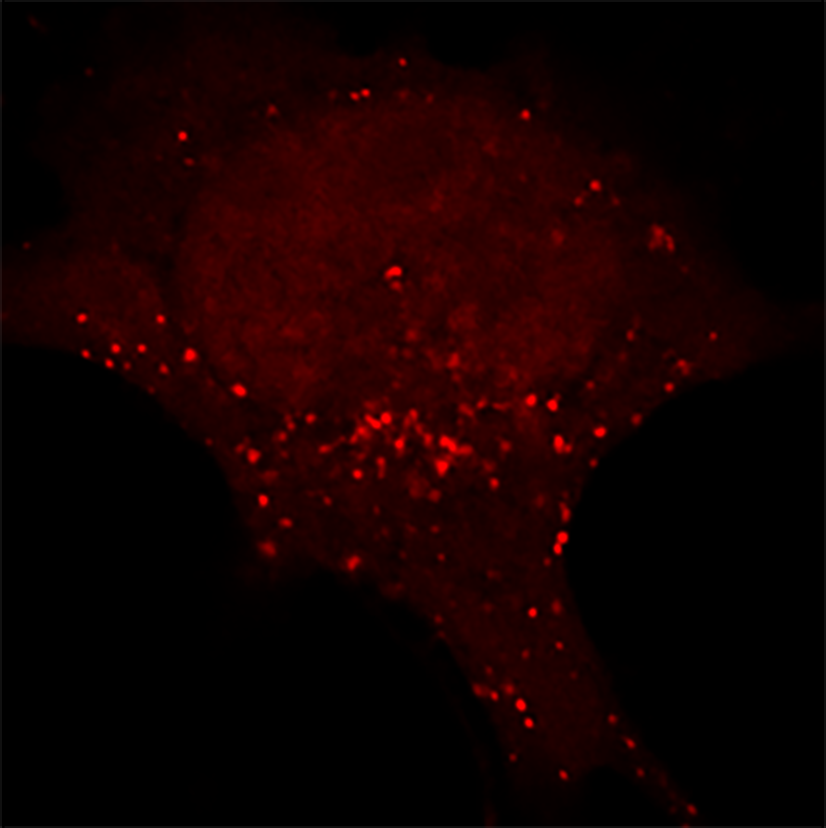

Supplement: Supplementary file 10 — Source data Fig. 5 [file 44318_2025_410_MOESM10_ESM.zip › Source Data For Figure 5/5E, F, J _microscopy/5J/2. Cherry-LC3B.tif]

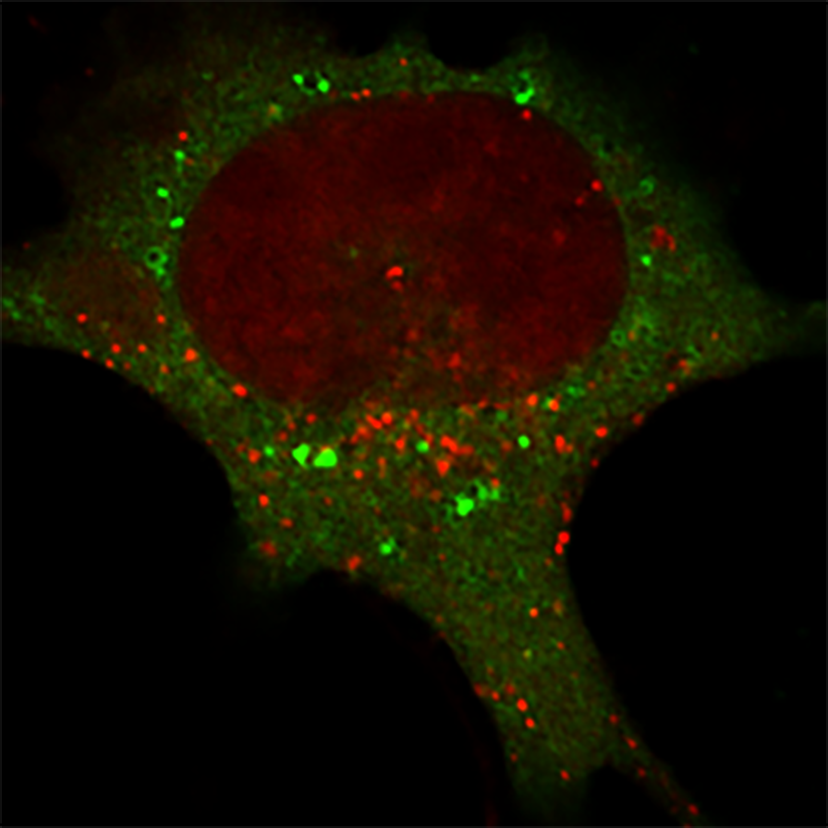

Supplement: Supplementary file 10 — Source data Fig. 5 [file 44318_2025_410_MOESM10_ESM.zip › Source Data For Figure 5/5E, F, J _microscopy/5J/3. Merged.tif]

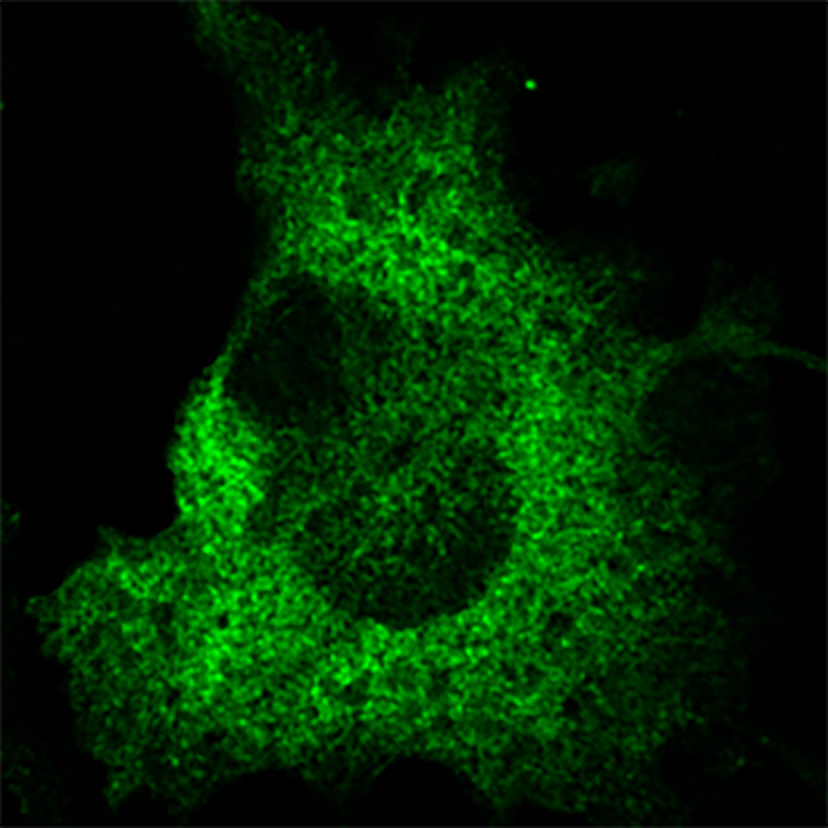

Supplement: Supplementary file 10 — Source data Fig. 5 [file 44318_2025_410_MOESM10_ESM.zip › Source Data For Figure 5/5E, F, J _microscopy/5J/4. GFP-ATG2A-ER.tif]

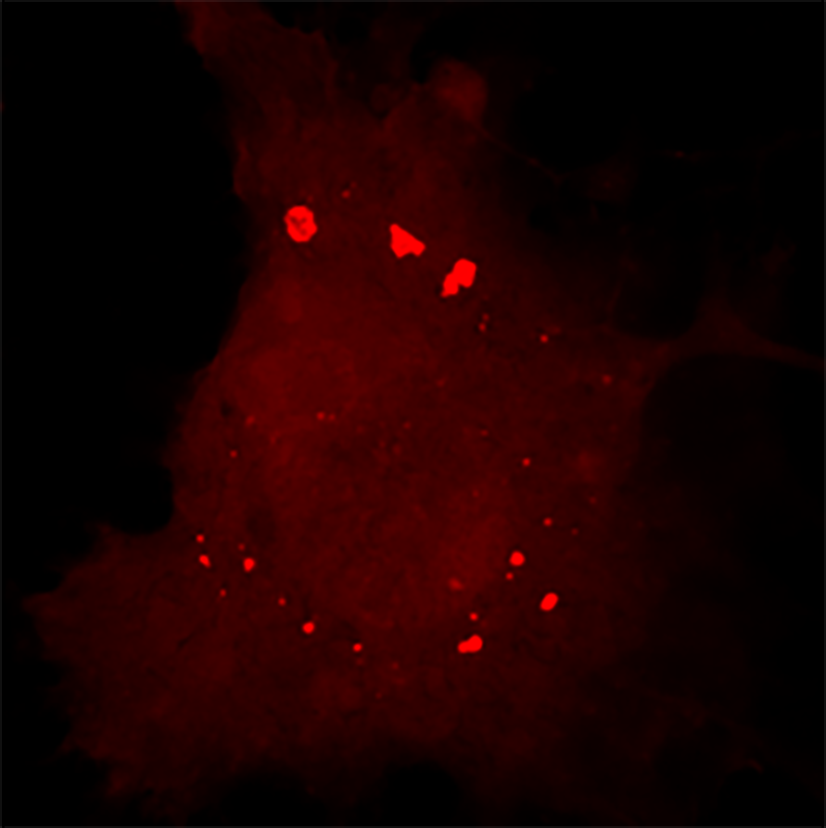

Supplement: Supplementary file 10 — Source data Fig. 5 [file 44318_2025_410_MOESM10_ESM.zip › Source Data For Figure 5/5E, F, J _microscopy/5J/5. Cherry-LC3B.tif]

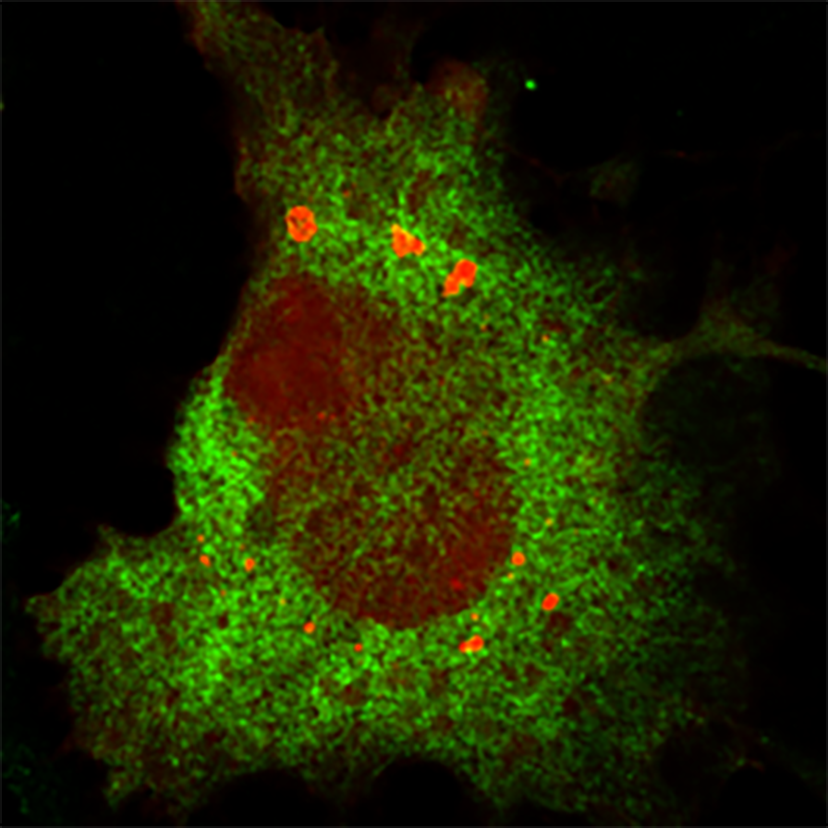

Supplement: Supplementary file 10 — Source data Fig. 5 [file 44318_2025_410_MOESM10_ESM.zip › Source Data For Figure 5/5E, F, J _microscopy/5J/6. Merged.tif]

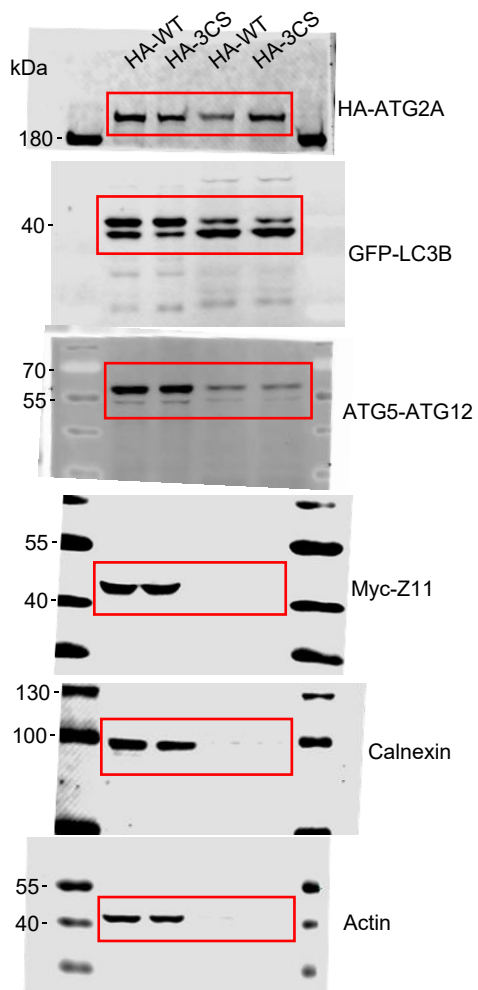

Fig 6A

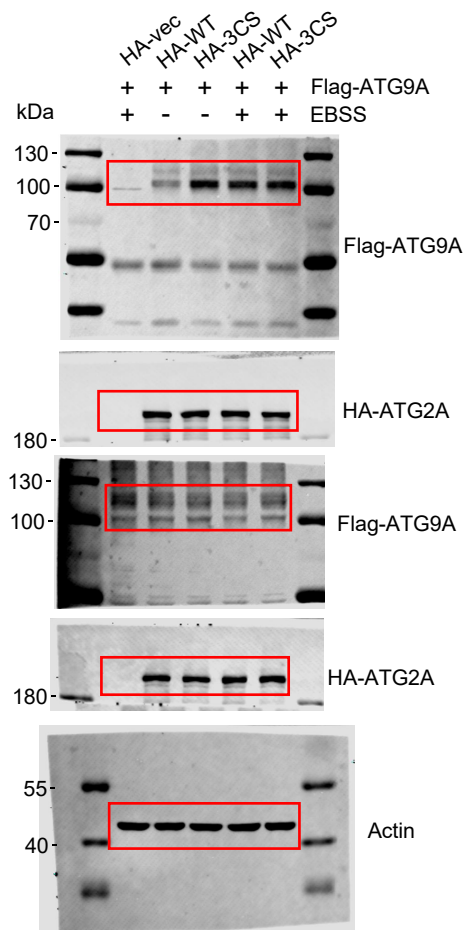

Fig 6G

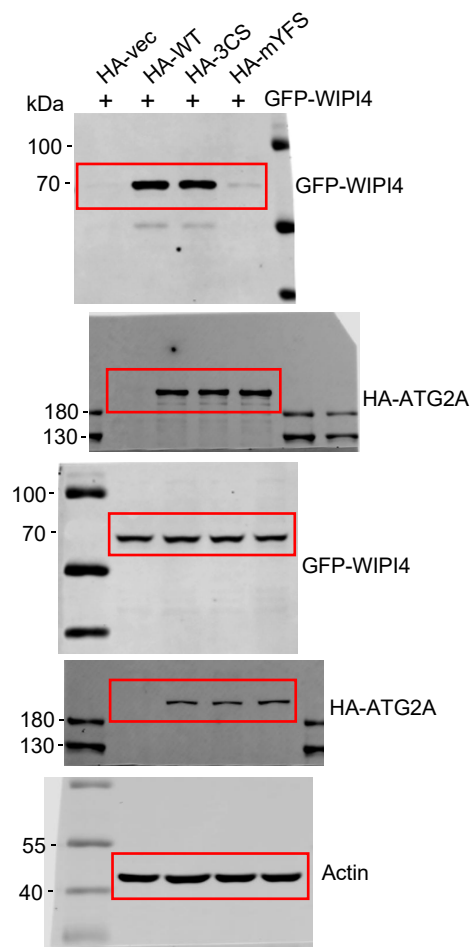

Fig 6H

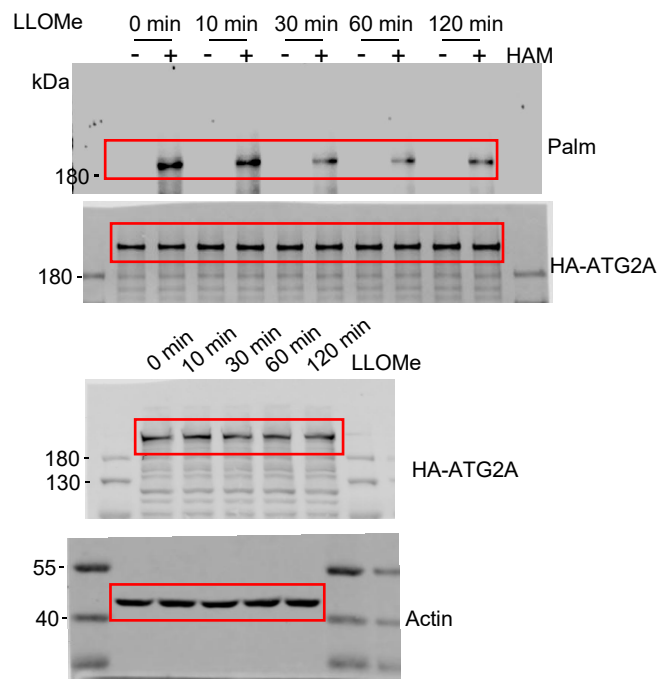

Fig 6I

Supplement: Supplementary file 11 — Source data Fig. 6 [file 44318_2025_410_MOESM11_ESM.zip › Source Data For Figure 6/6A, G, H, I _western blot/Figure 6.pdf]

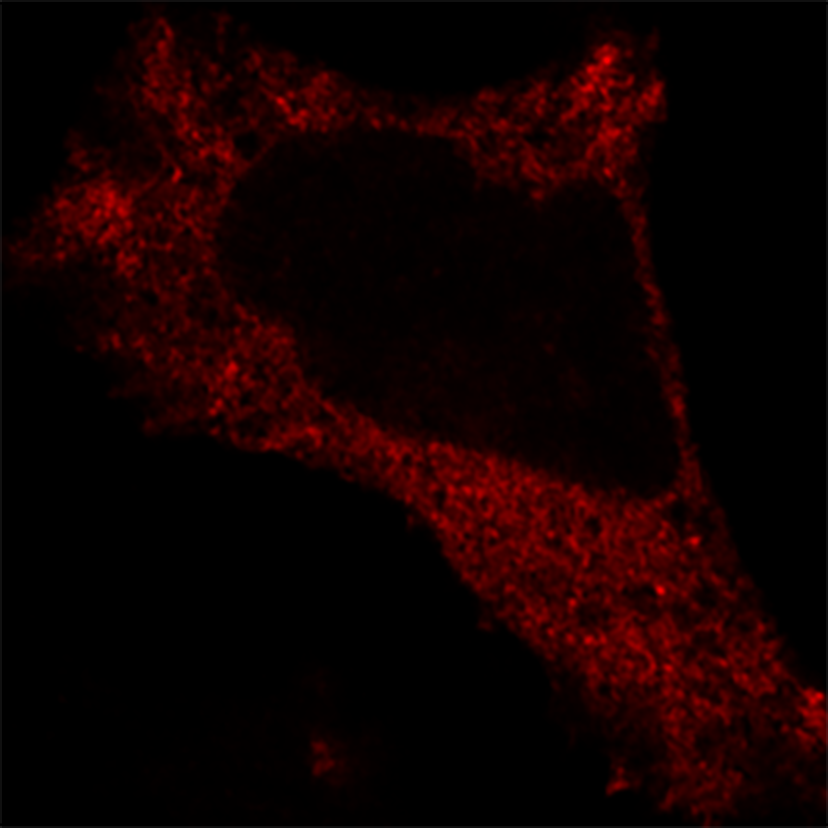

Supplement: Supplementary file 11 — Source data Fig. 6 [file 44318_2025_410_MOESM11_ESM.zip › Source Data For Figure 6/6C, E, K _microscopy/6C/1. HA-WT.tif]

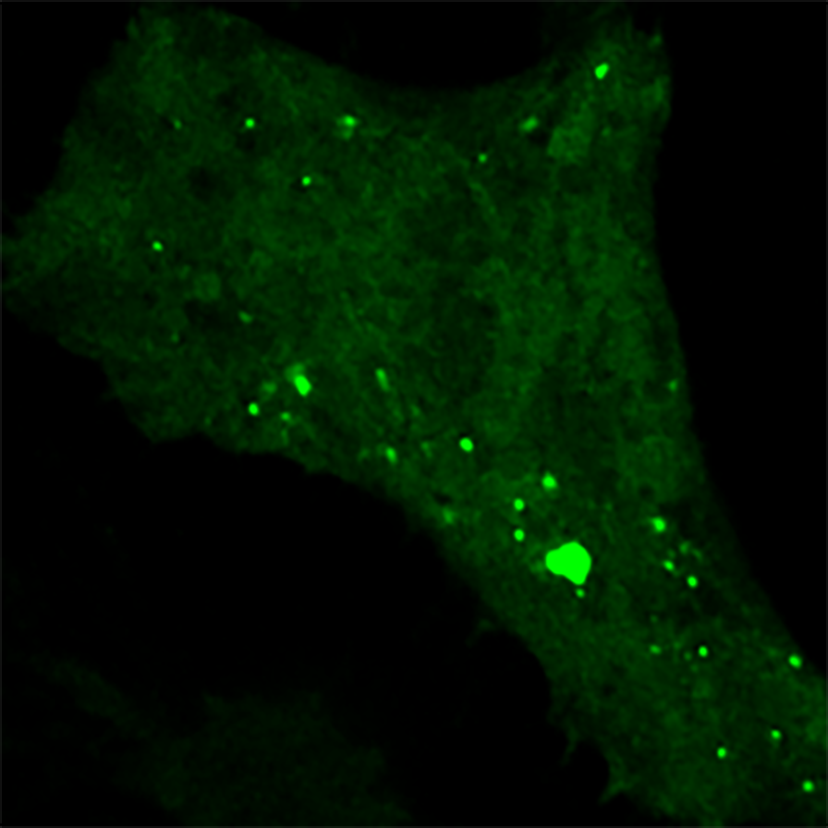

Supplement: Supplementary file 11 — Source data Fig. 6 [file 44318_2025_410_MOESM11_ESM.zip › Source Data For Figure 6/6C, E, K _microscopy/6C/2. GFP-LC3B.tif]

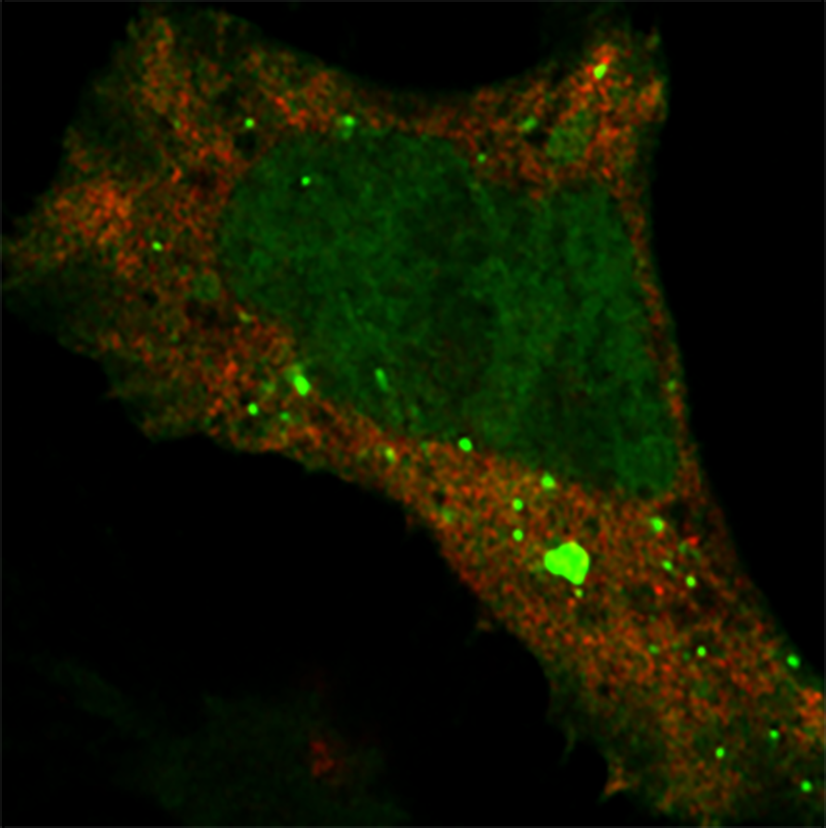

Supplement: Supplementary file 11 — Source data Fig. 6 [file 44318_2025_410_MOESM11_ESM.zip › Source Data For Figure 6/6C, E, K _microscopy/6C/3. Merged.tif]

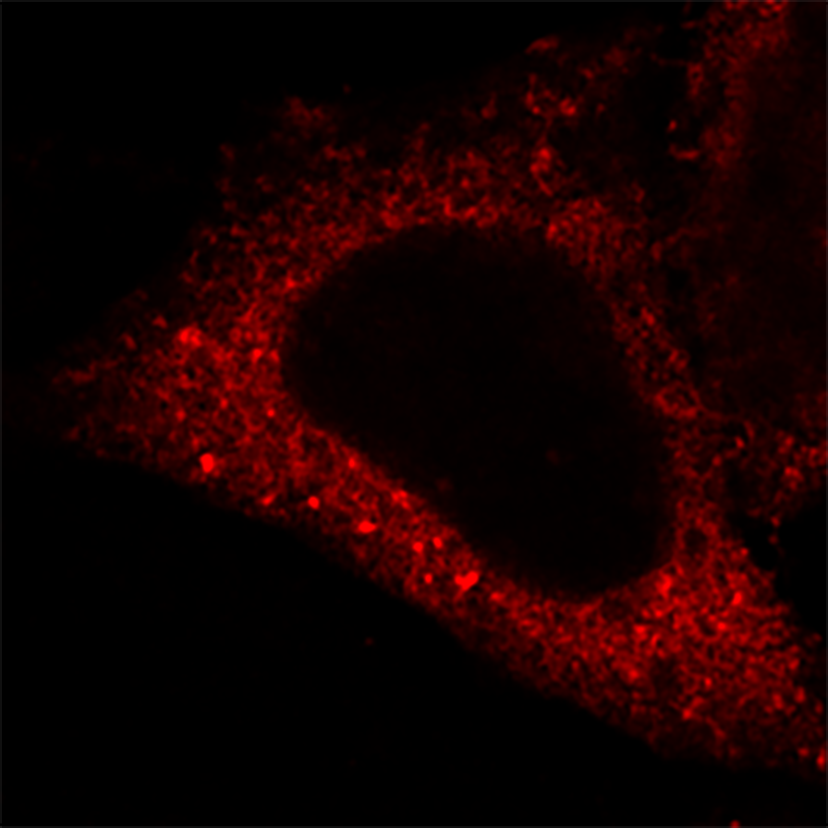

Supplement: Supplementary file 11 — Source data Fig. 6 [file 44318_2025_410_MOESM11_ESM.zip › Source Data For Figure 6/6C, E, K _microscopy/6C/4. HA-3CS.tif]

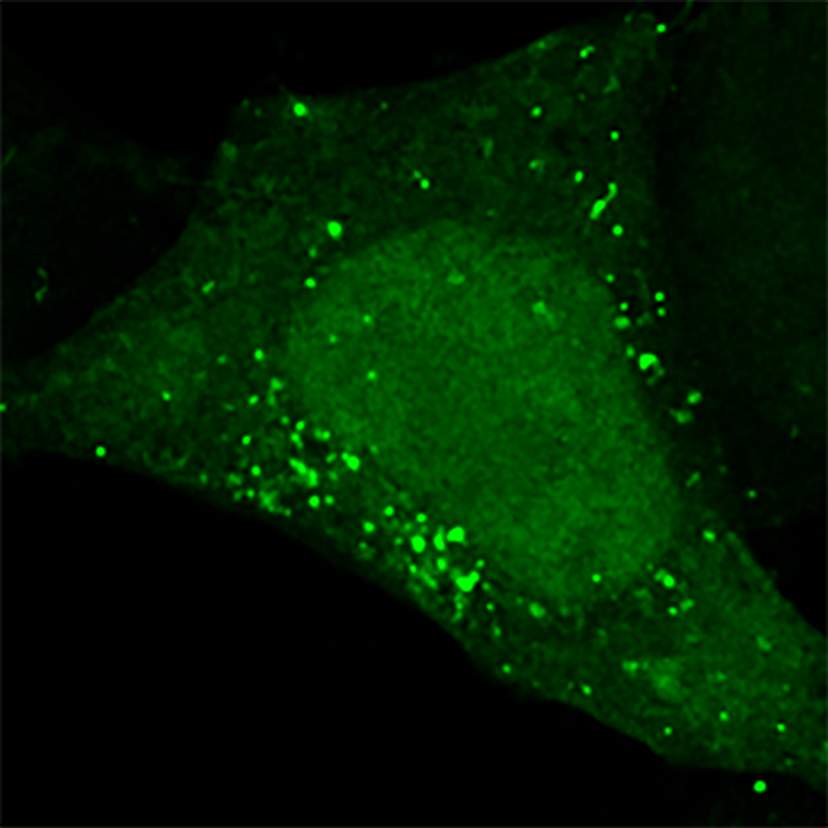

Supplement: Supplementary file 11 — Source data Fig. 6 [file 44318_2025_410_MOESM11_ESM.zip › Source Data For Figure 6/6C, E, K _microscopy/6C/5. GFP-LC3B.tif]

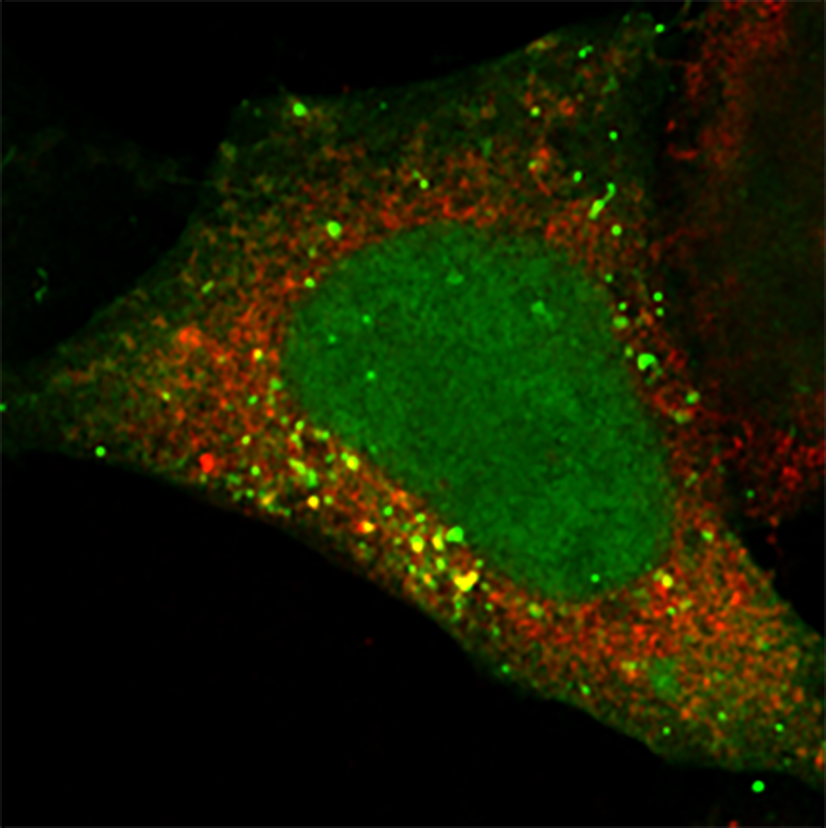

Supplement: Supplementary file 11 — Source data Fig. 6 [file 44318_2025_410_MOESM11_ESM.zip › Source Data For Figure 6/6C, E, K _microscopy/6C/6. Merged.tif]

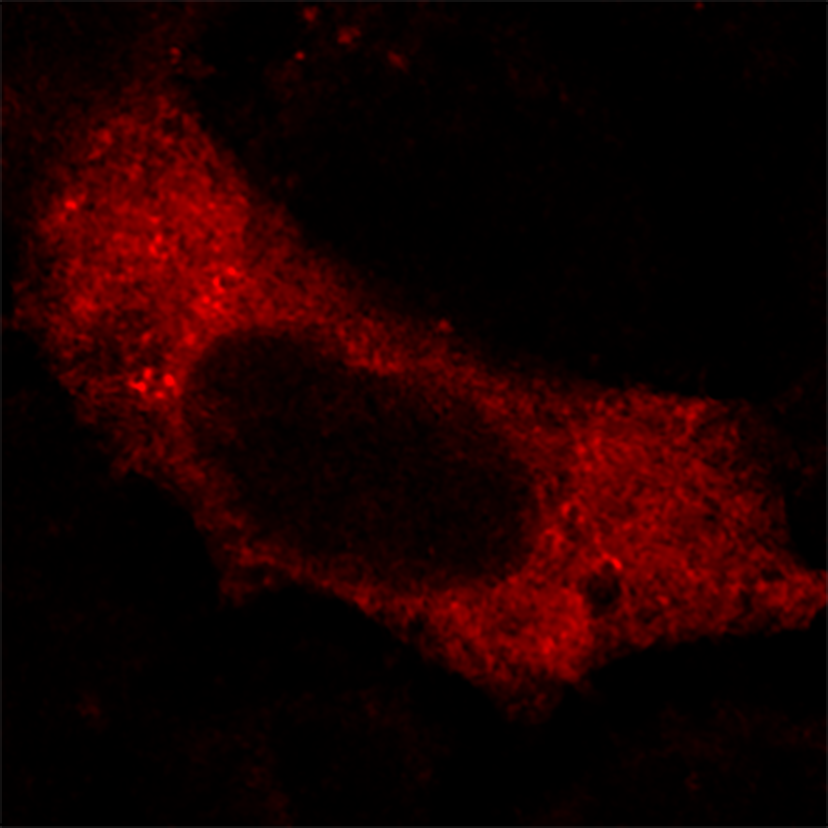

Supplement: Supplementary file 11 — Source data Fig. 6 [file 44318_2025_410_MOESM11_ESM.zip › Source Data For Figure 6/6C, E, K _microscopy/6E/1. Cherry-WT.tif]

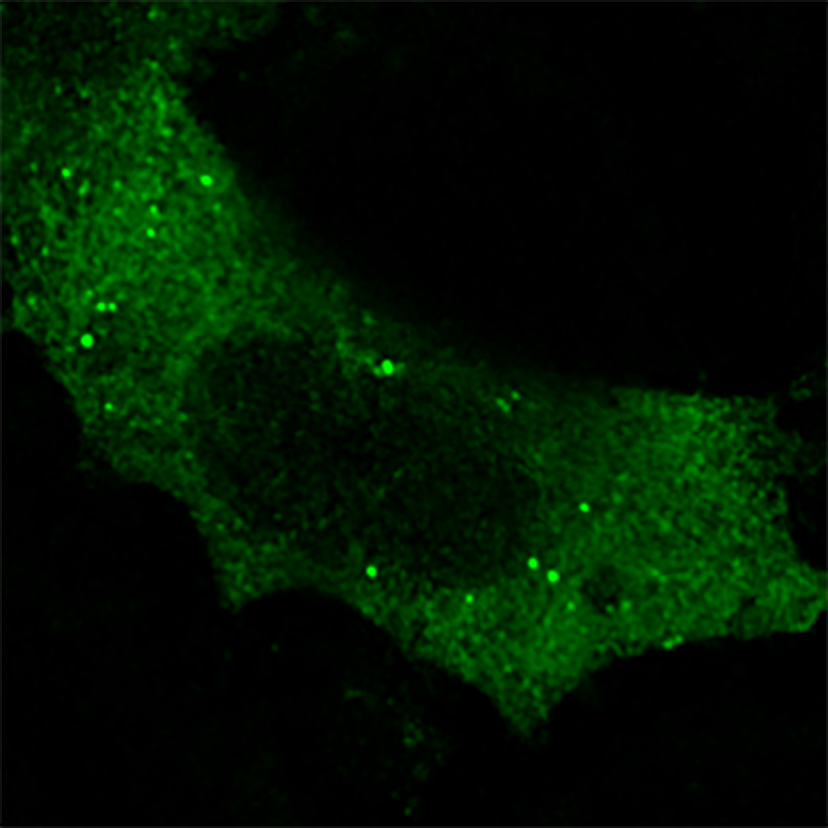

Supplement: Supplementary file 11 — Source data Fig. 6 [file 44318_2025_410_MOESM11_ESM.zip › Source Data For Figure 6/6C, E, K _microscopy/6E/2. GFP-FIP200.tif]

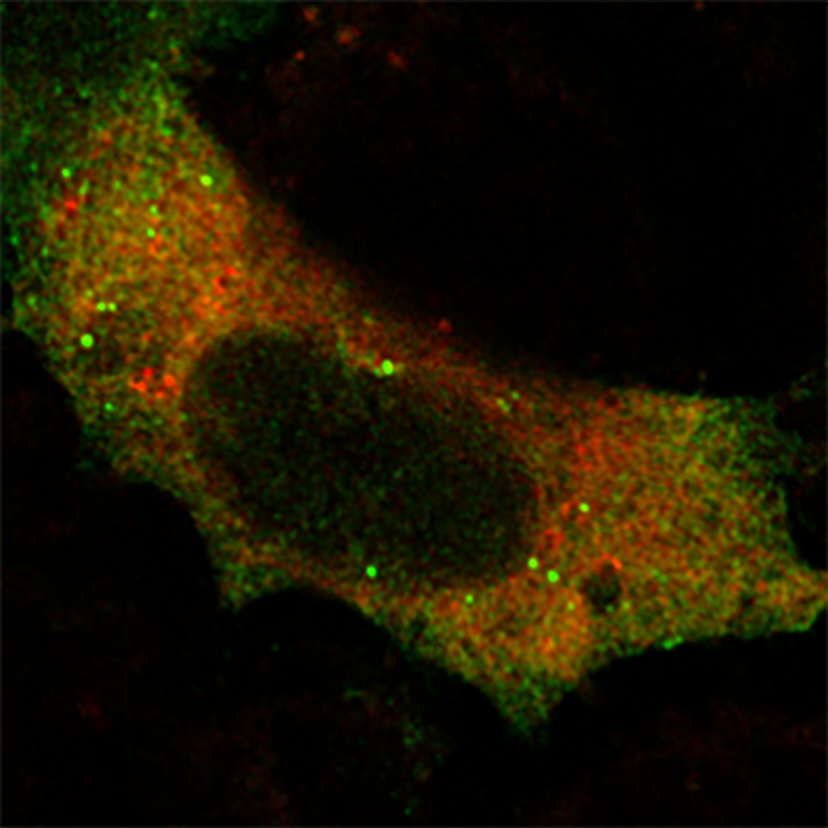

Supplement: Supplementary file 11 — Source data Fig. 6 [file 44318_2025_410_MOESM11_ESM.zip › Source Data For Figure 6/6C, E, K _microscopy/6E/3. Merged.tif]

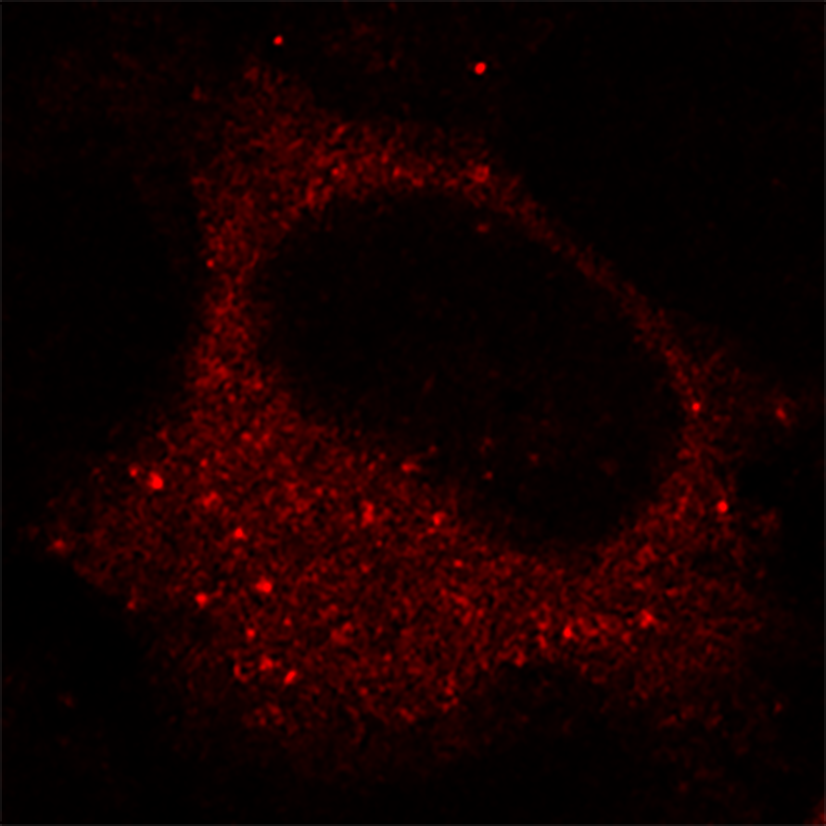

Supplement: Supplementary file 11 — Source data Fig. 6 [file 44318_2025_410_MOESM11_ESM.zip › Source Data For Figure 6/6C, E, K _microscopy/6E/4. Cherry-3CS.tif]

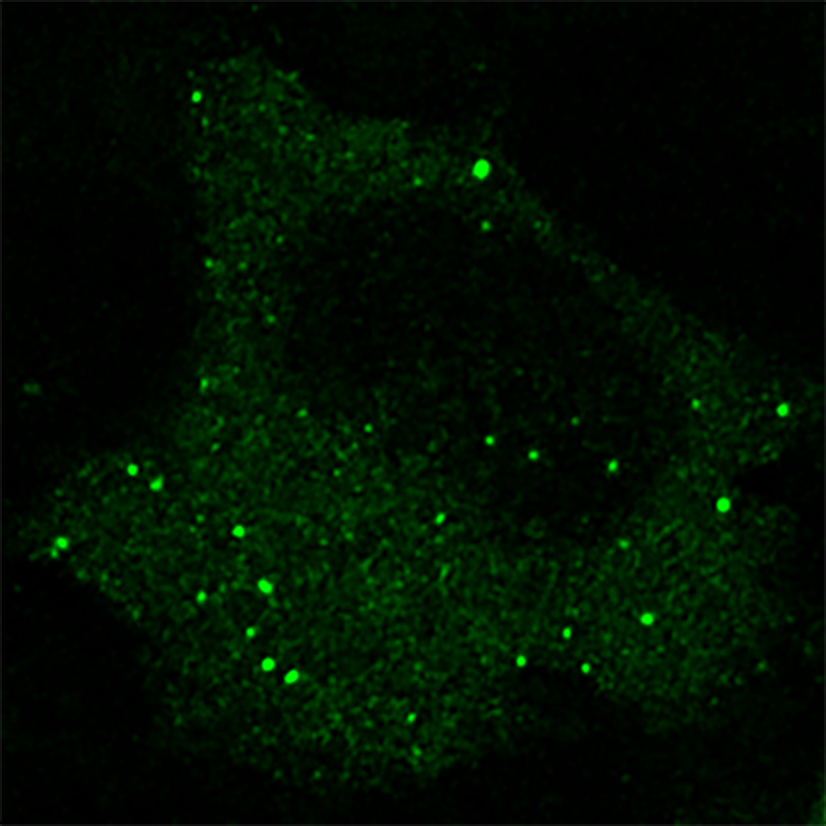

Supplement: Supplementary file 11 — Source data Fig. 6 [file 44318_2025_410_MOESM11_ESM.zip › Source Data For Figure 6/6C, E, K _microscopy/6E/5. GFP-FIP200.tif]

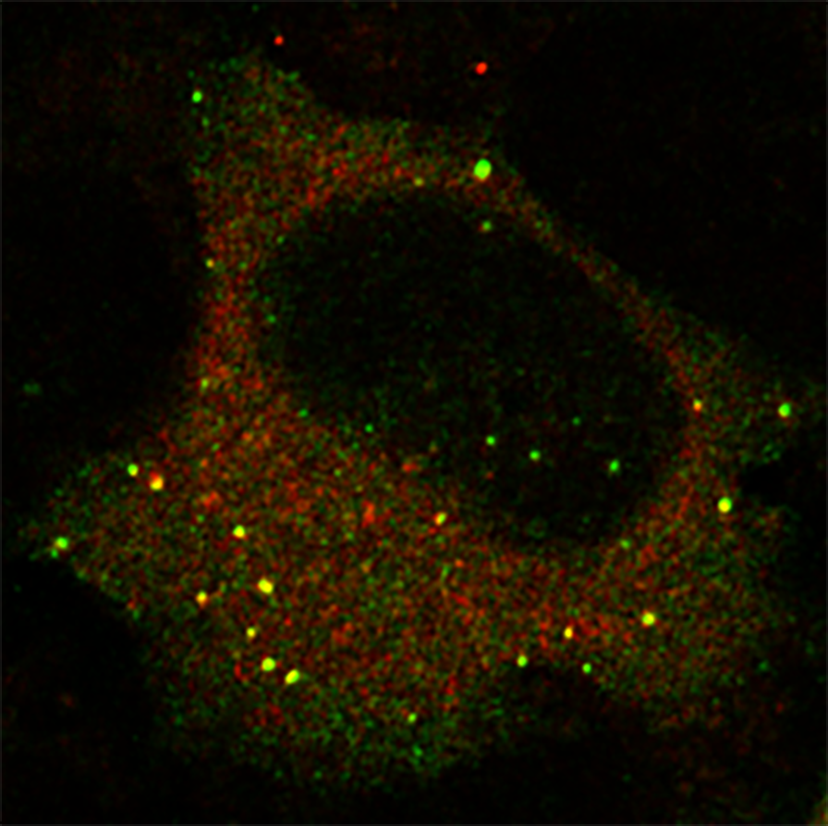

Supplement: Supplementary file 11 — Source data Fig. 6 [file 44318_2025_410_MOESM11_ESM.zip › Source Data For Figure 6/6C, E, K _microscopy/6E/6. Merged.tif]

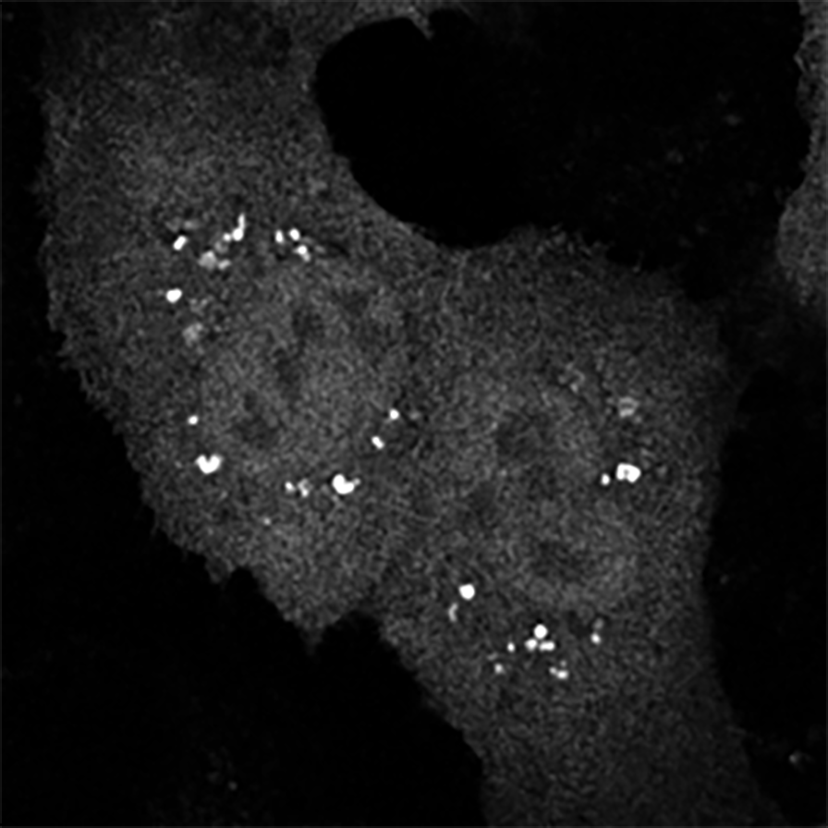

Supplement: Supplementary file 11 — Source data Fig. 6 [file 44318_2025_410_MOESM11_ESM.zip › Source Data For Figure 6/6C, E, K _microscopy/6K/1. HA-ATG2A_GFP-GAL-3.tif]

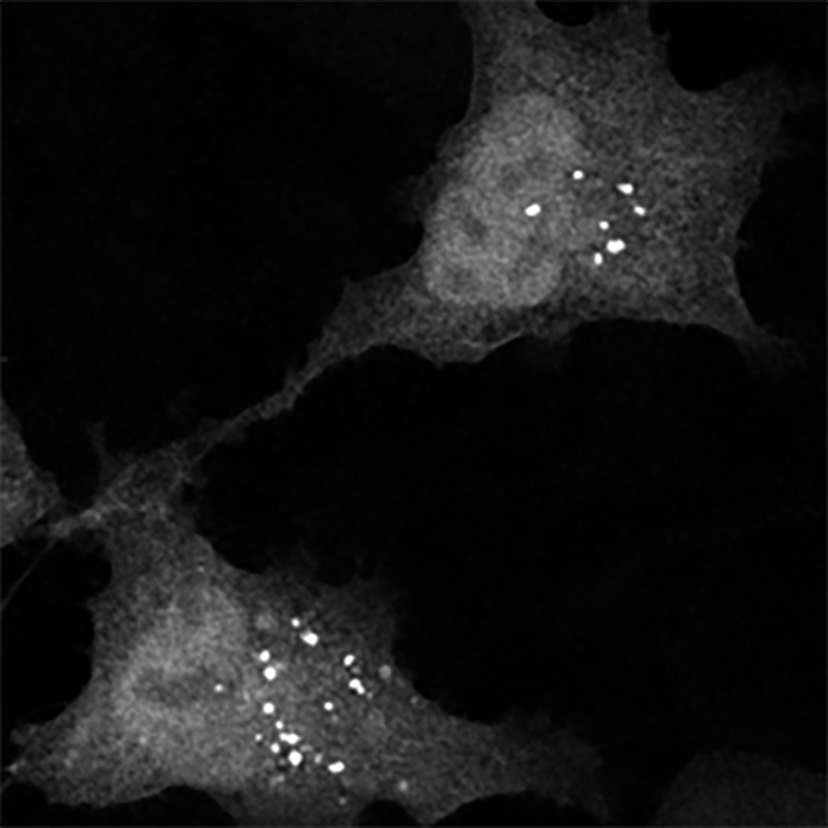

Supplement: Supplementary file 11 — Source data Fig. 6 [file 44318_2025_410_MOESM11_ESM.zip › Source Data For Figure 6/6C, E, K _microscopy/6K/2. HA-ATG2A-3CS_GFP-GAL-3.tif]

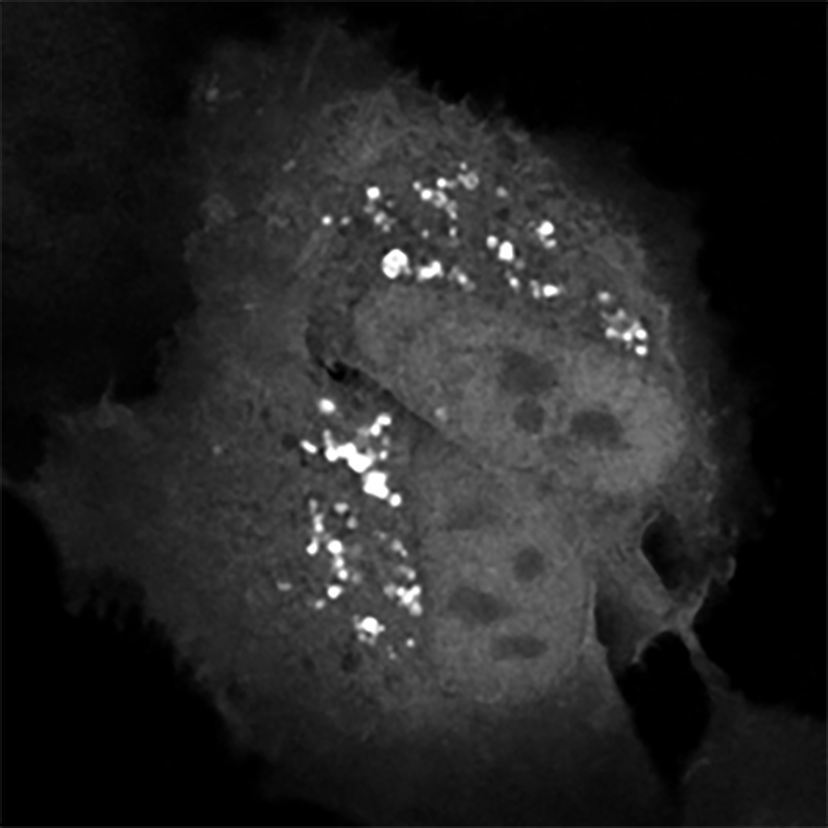

Supplement: Supplementary file 11 — Source data Fig. 6 [file 44318_2025_410_MOESM11_ESM.zip › Source Data For Figure 6/6C, E, K _microscopy/6K/3. HA-ATG2A + Myc-Z11_GFP-GAL-3.tif]

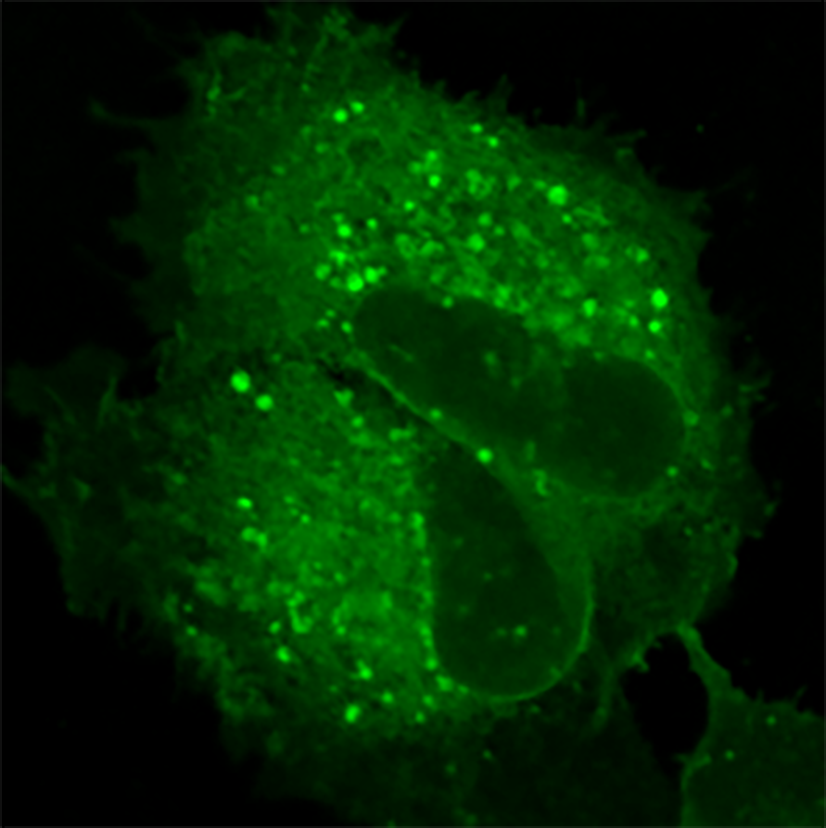

Supplement: Supplementary file 11 — Source data Fig. 6 [file 44318_2025_410_MOESM11_ESM.zip › Source Data For Figure 6/6C, E, K _microscopy/6K/3. HA-ATG2A + Myc-Z11_Myc-Z11.tif]

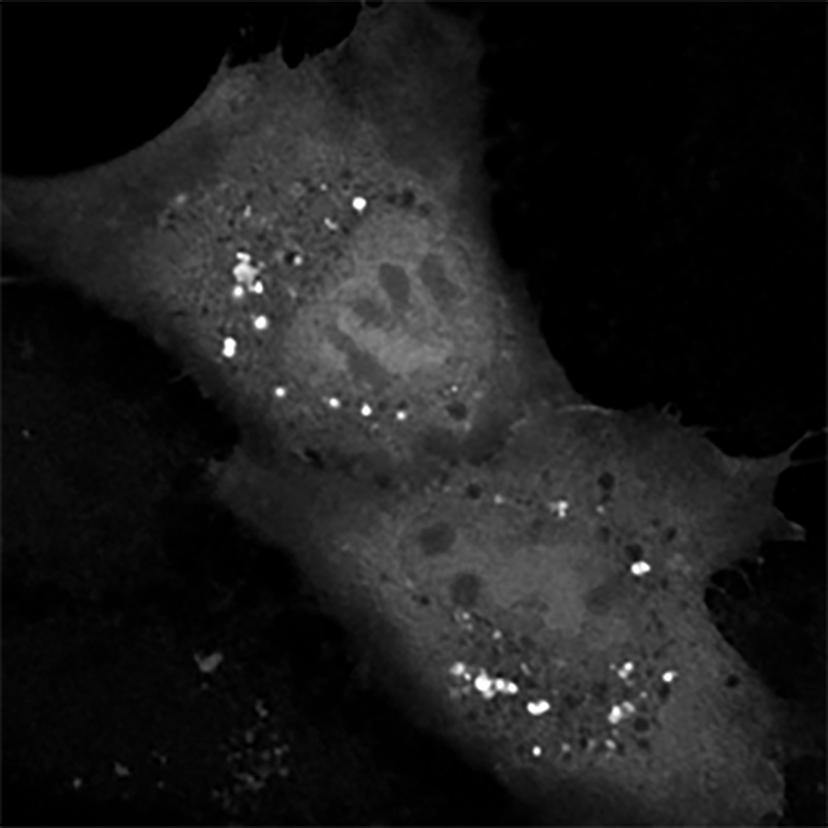

Supplement: Supplementary file 11 — Source data Fig. 6 [file 44318_2025_410_MOESM11_ESM.zip › Source Data For Figure 6/6C, E, K _microscopy/6K/4. HA-ATG2A-3CS + Myc-Z11_GFP-GAL-3.tif]

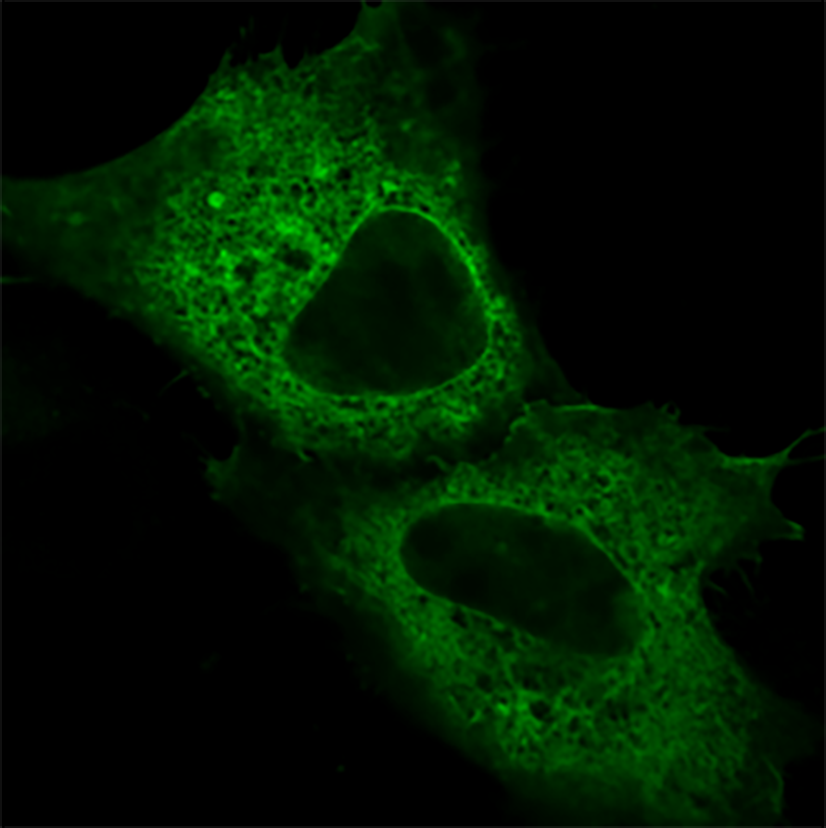

Supplement: Supplementary file 11 — Source data Fig. 6 [file 44318_2025_410_MOESM11_ESM.zip › Source Data For Figure 6/6C, E, K _microscopy/6K/4. HA-ATG2A-3CS + Myc-Z11_Myc-Z11.tif]

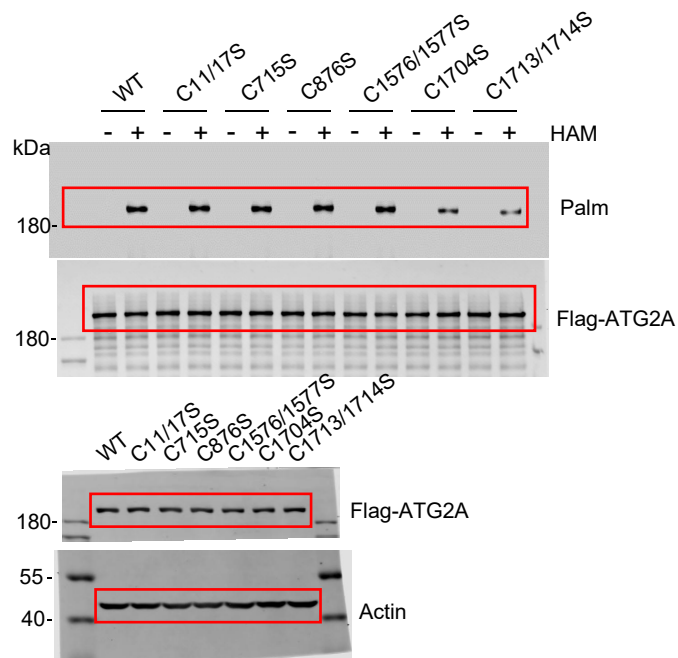

Fig EV1F

Supplement: Supplementary file 12 — Figures EV1-5 Source Data [file 44318_2025_410_MOESM12_ESM.zip › Source Data For EV Figures/Source Data For Figure EV1/EV1F _western blot/Figure EV1F.pdf]

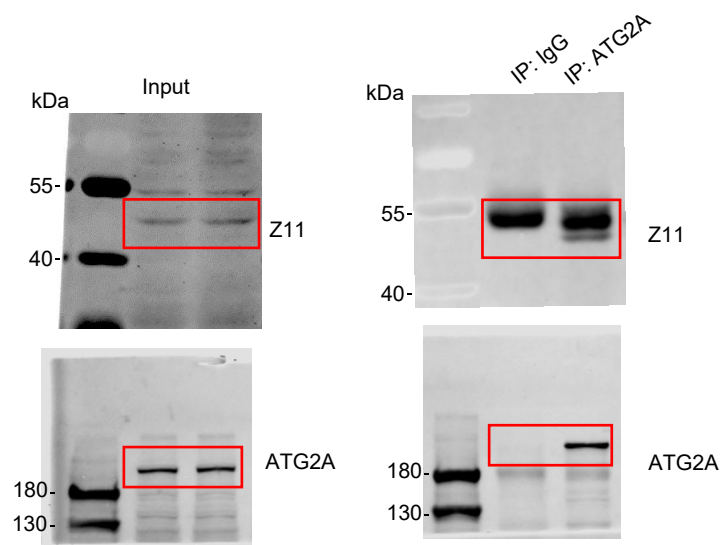

Fig EV2B

Supplement: Supplementary file 12 — Figures EV1-5 Source Data [file 44318_2025_410_MOESM12_ESM.zip › Source Data For EV Figures/Source Data For Figure EV2/EV2B _western blot/Figure EV2B.pdf]

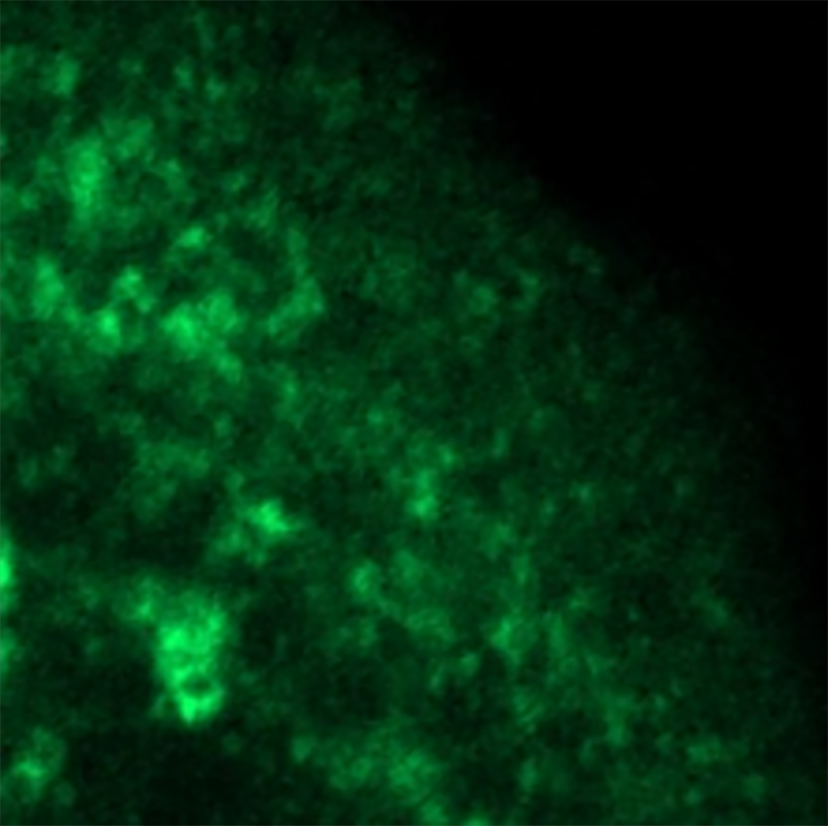

Supplement: Supplementary file 12 — Figures EV1-5 Source Data [file 44318_2025_410_MOESM12_ESM.zip › Source Data For EV Figures/Source Data For Figure EV2/EV2C, D _microscopy/EV2C/GFP-ATG2A.tif]

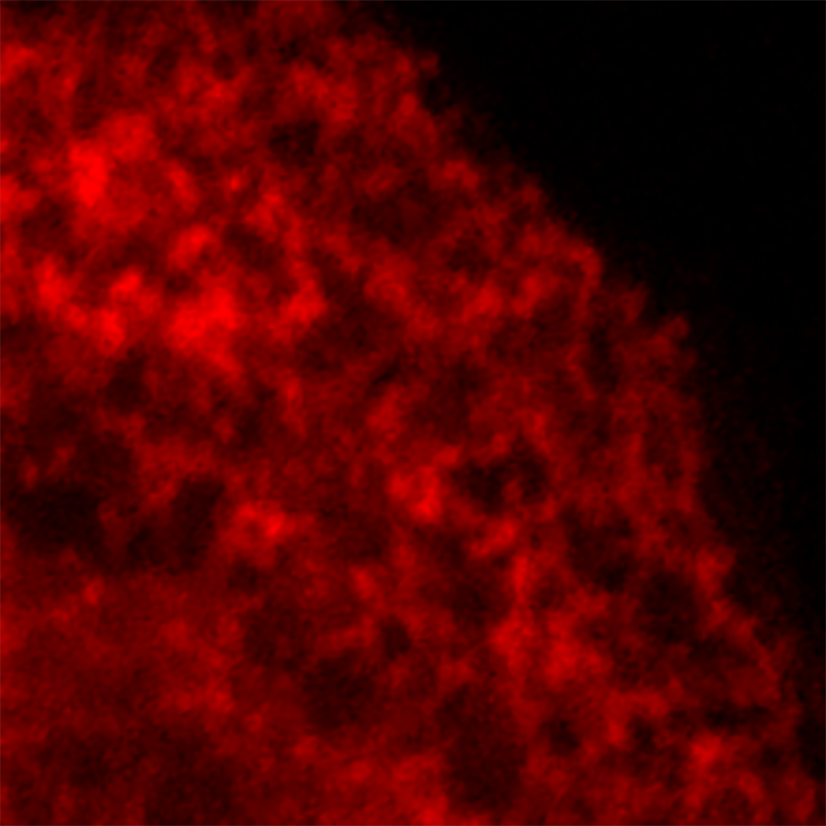

Supplement: Supplementary file 12 — Figures EV1-5 Source Data [file 44318_2025_410_MOESM12_ESM.zip › Source Data For EV Figures/Source Data For Figure EV2/EV2C, D _microscopy/EV2C/HA-Z11.tif]

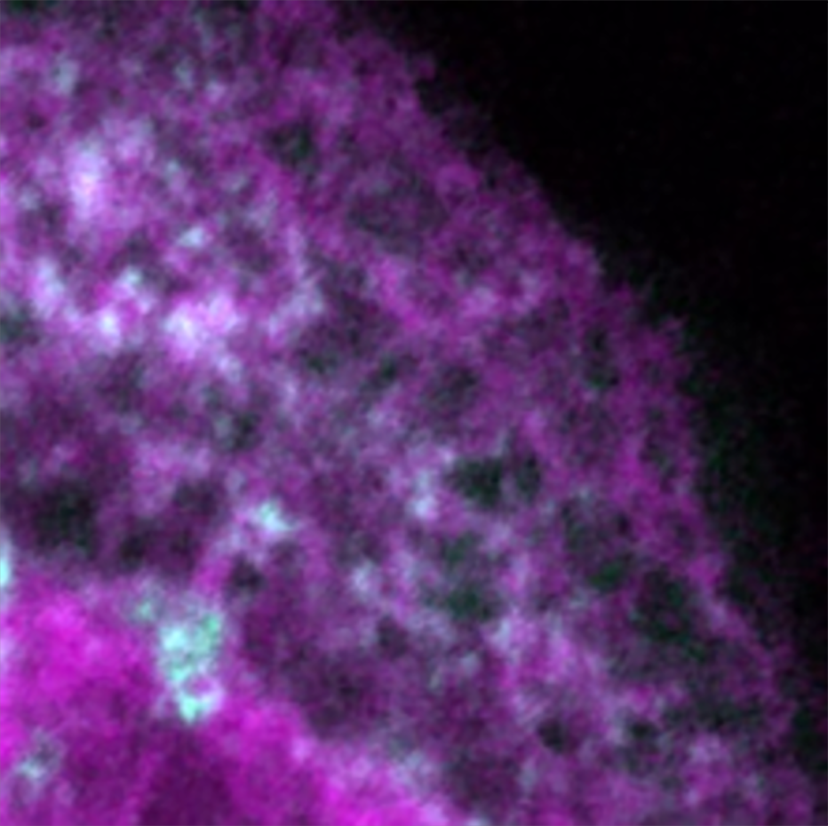

Supplement: Supplementary file 12 — Figures EV1-5 Source Data [file 44318_2025_410_MOESM12_ESM.zip › Source Data For EV Figures/Source Data For Figure EV2/EV2C, D _microscopy/EV2C/Merged.tif]

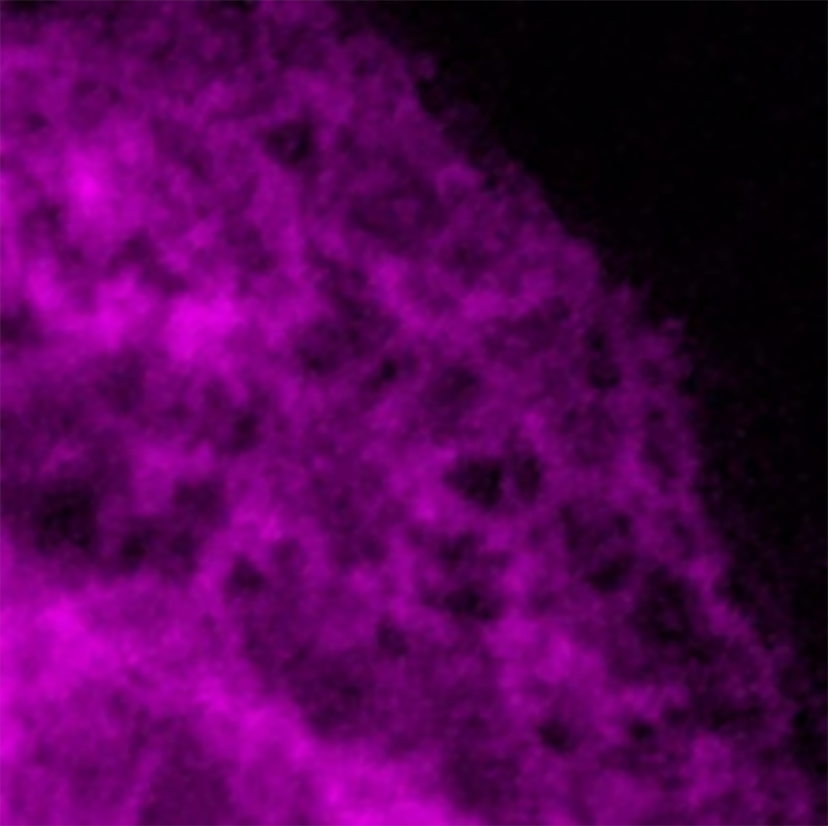

Supplement: Supplementary file 12 — Figures EV1-5 Source Data [file 44318_2025_410_MOESM12_ESM.zip › Source Data For EV Figures/Source Data For Figure EV2/EV2C, D _microscopy/EV2C/mCh-Sec61β.tif]

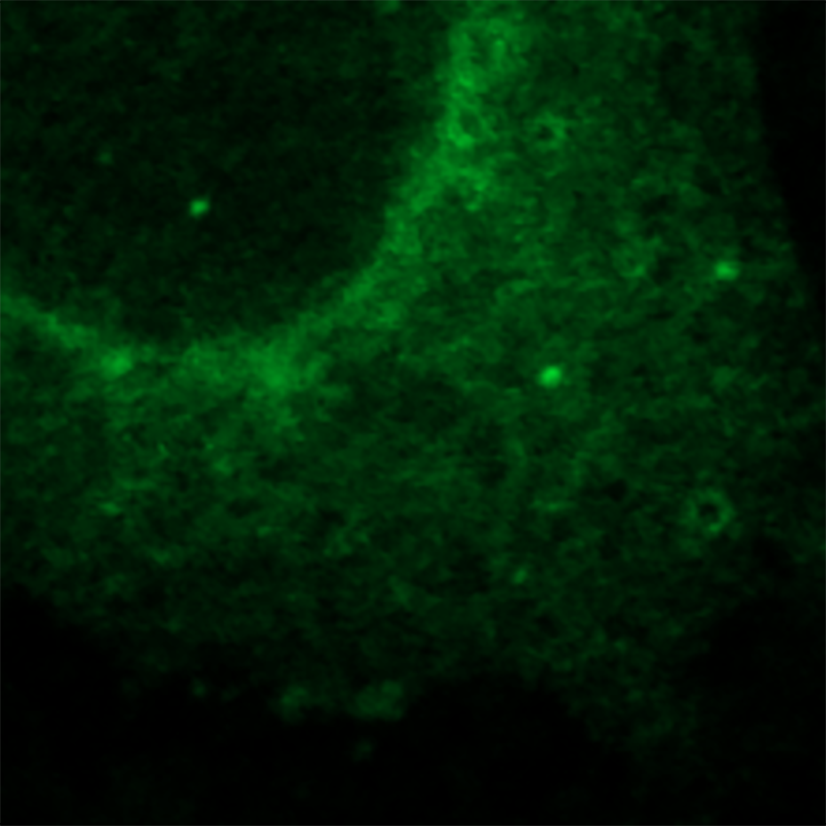

Supplement: Supplementary file 12 — Figures EV1-5 Source Data [file 44318_2025_410_MOESM12_ESM.zip › Source Data For EV Figures/Source Data For Figure EV2/EV2C, D _microscopy/EV2D/GFP-ATG2A.tif]

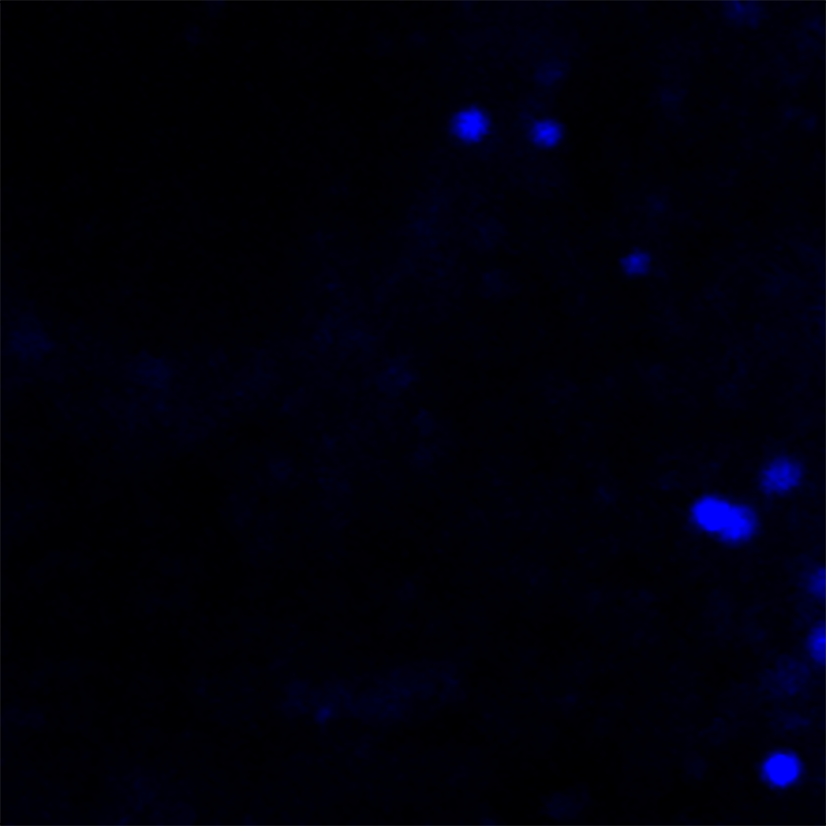

Supplement: Supplementary file 12 — Figures EV1-5 Source Data [file 44318_2025_410_MOESM12_ESM.zip › Source Data For EV Figures/Source Data For Figure EV2/EV2C, D _microscopy/EV2D/Lipi-Blue.tif]

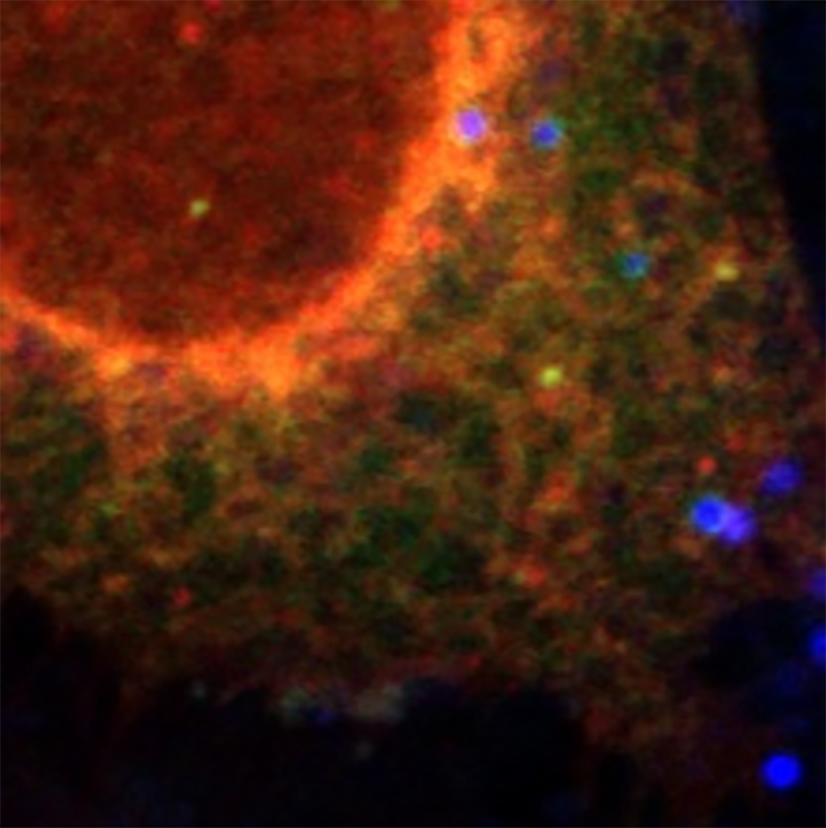

Supplement: Supplementary file 12 — Figures EV1-5 Source Data [file 44318_2025_410_MOESM12_ESM.zip › Source Data For EV Figures/Source Data For Figure EV2/EV2C, D _microscopy/EV2D/Merged.tif]

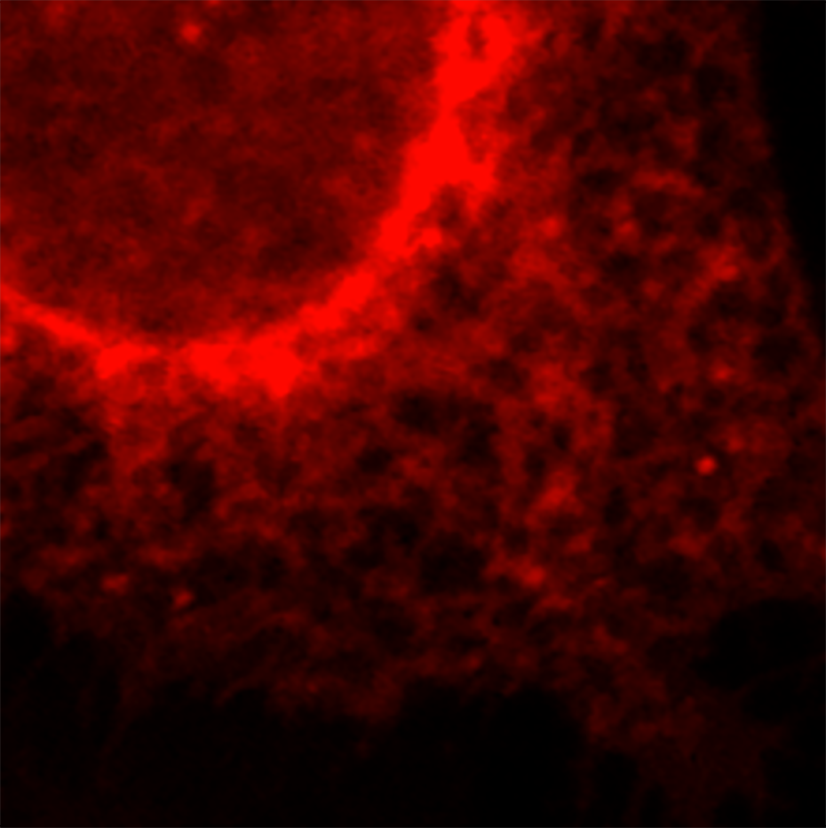

Supplement: Supplementary file 12 — Figures EV1-5 Source Data [file 44318_2025_410_MOESM12_ESM.zip › Source Data For EV Figures/Source Data For Figure EV2/EV2C, D _microscopy/EV2D/mCh-Z11.tif]
