# Supplementary figures and images for: S-palmitoylation modulates ATG2-dependent non-vesicular lipid transport during starvation-induced autophagy (part 2 of 2)
Source: EMBO J. 2025 Mar 24;44(9):2596–619. doi: 10.1038/s44318-025-00410-7 (PMC12048663; doi:10.1038/s44318-025-00410-7)

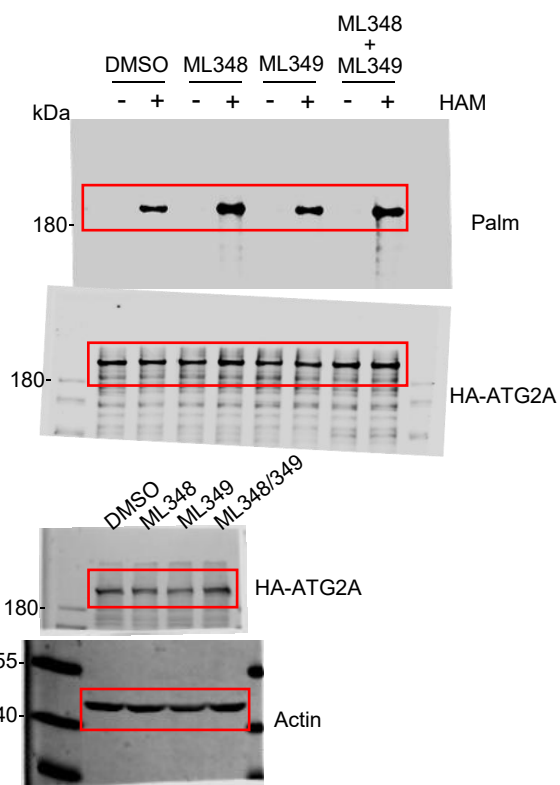

Fig EV3C

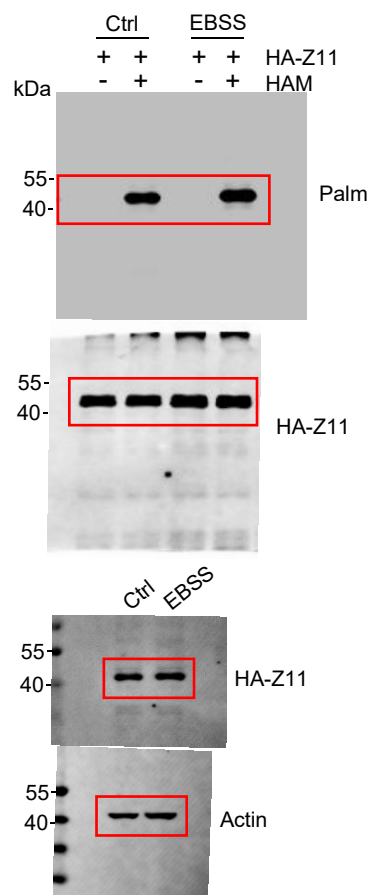

Fig EV3G

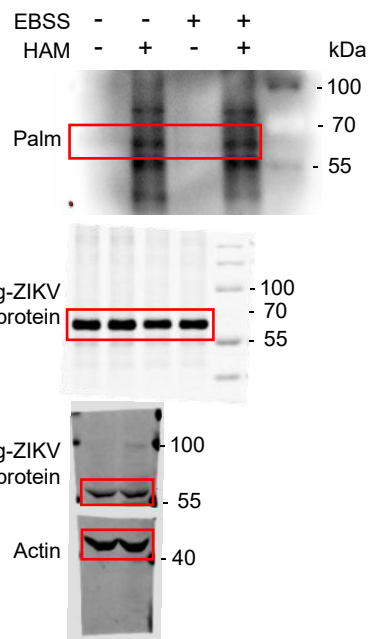

Fig EV31

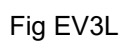

Supplement: Supplementary file 12 — Figures EV1-5 Source Data [file 44318_2025_410_MOESM12_ESM.zip › Source Data For EV Figures/Source Data For Figure EV3/EV3C, G, I, L _western blot/Figure EV3.pdf]

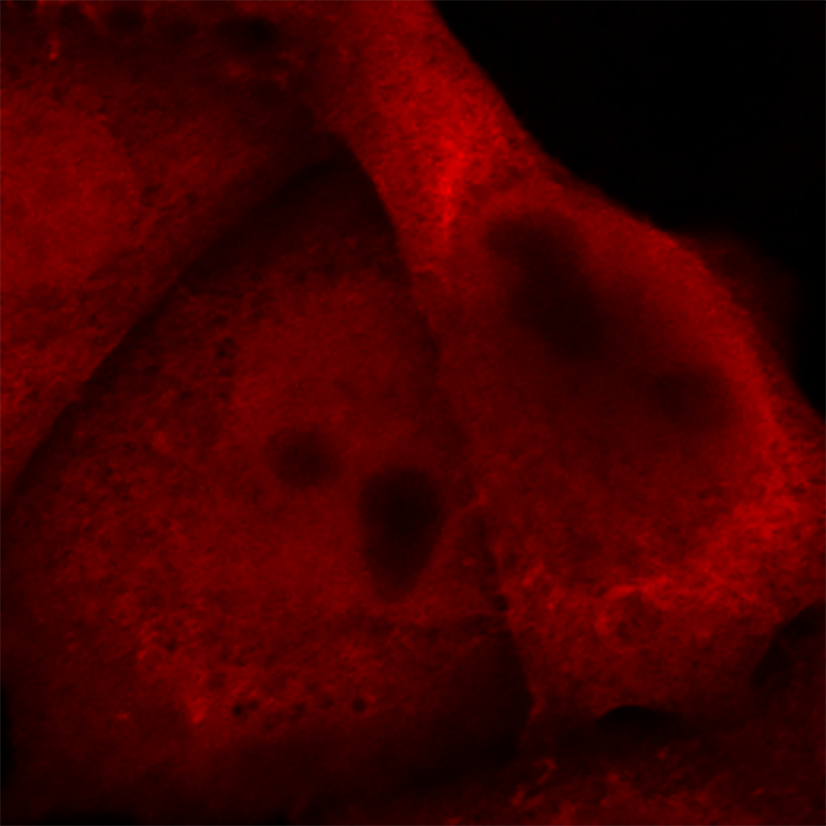

Supplement: Supplementary file 12 — Figures EV1-5 Source Data [file 44318_2025_410_MOESM12_ESM.zip › Source Data For EV Figures/Source Data For Figure EV3/EV3K _microscopy/Ctrl.tif]

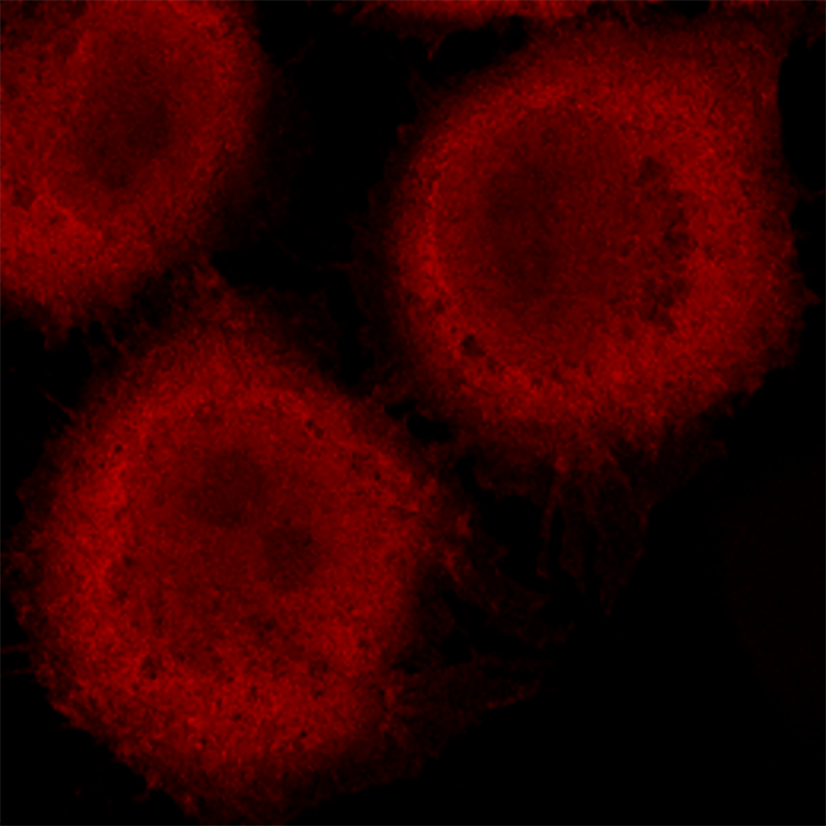

Supplement: Supplementary file 12 — Figures EV1-5 Source Data [file 44318_2025_410_MOESM12_ESM.zip › Source Data For EV Figures/Source Data For Figure EV3/EV3K _microscopy/EBSS.tif]

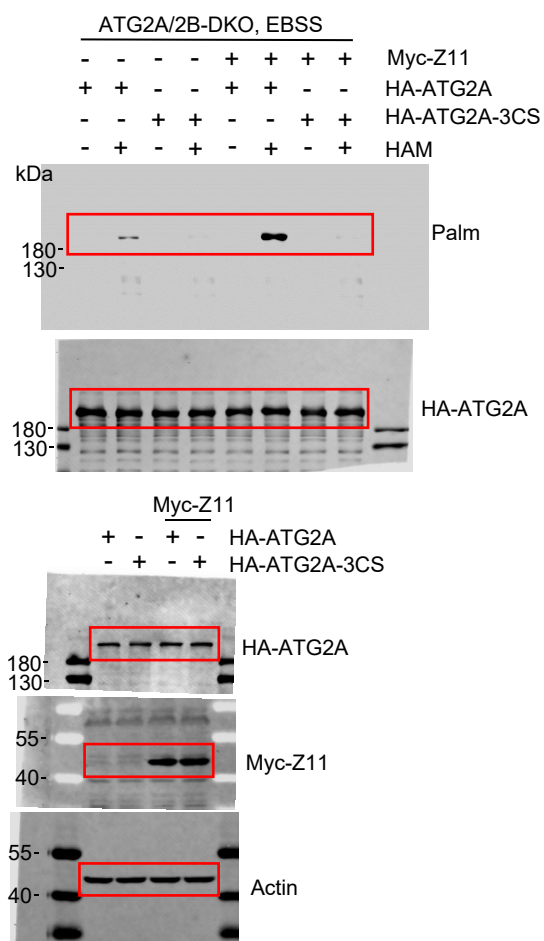

Fig EV4C

Supplement: Supplementary file 12 — Figures EV1-5 Source Data [file 44318_2025_410_MOESM12_ESM.zip › Source Data For EV Figures/Source Data For Figure EV4/EV4C _western blot/Figure EV4C.pdf]

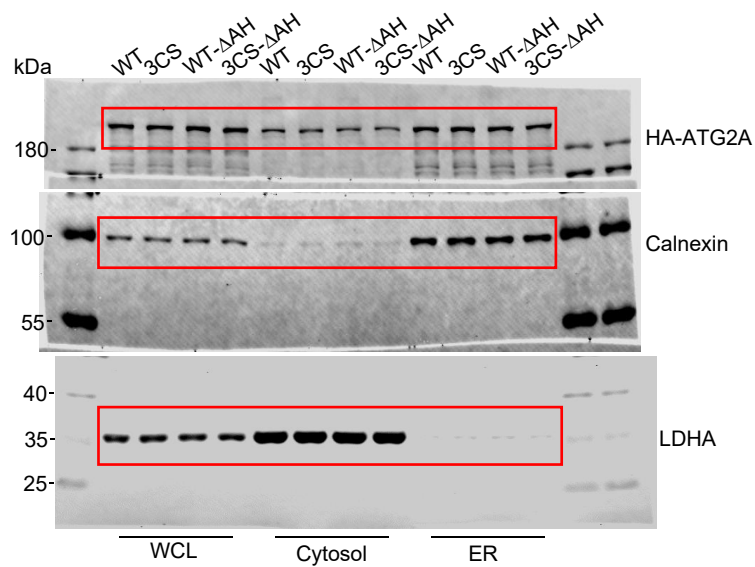

Fig EV5A

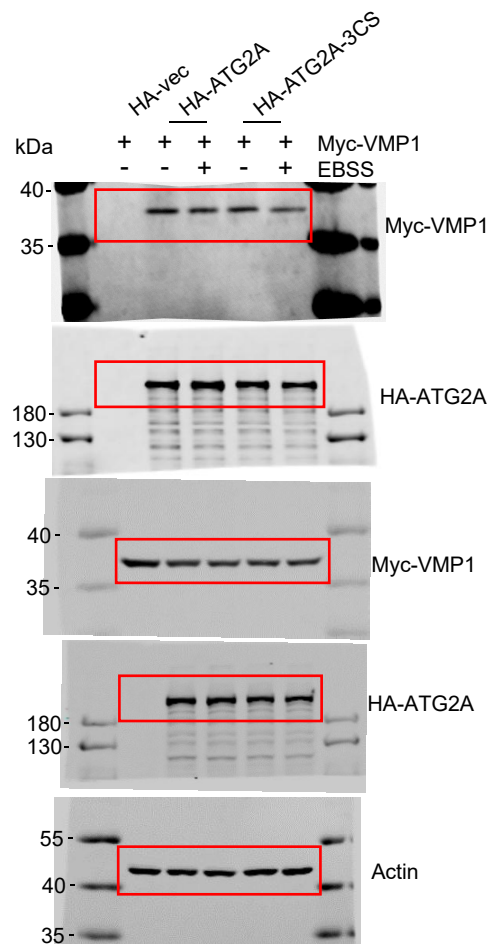

Fig EV5E

Supplement: Supplementary file 12 — Figures EV1-5 Source Data [file 44318_2025_410_MOESM12_ESM.zip › Source Data For EV Figures/Source Data For Figure EV5/EV5A, E _western blot/Figure EV5.pdf]

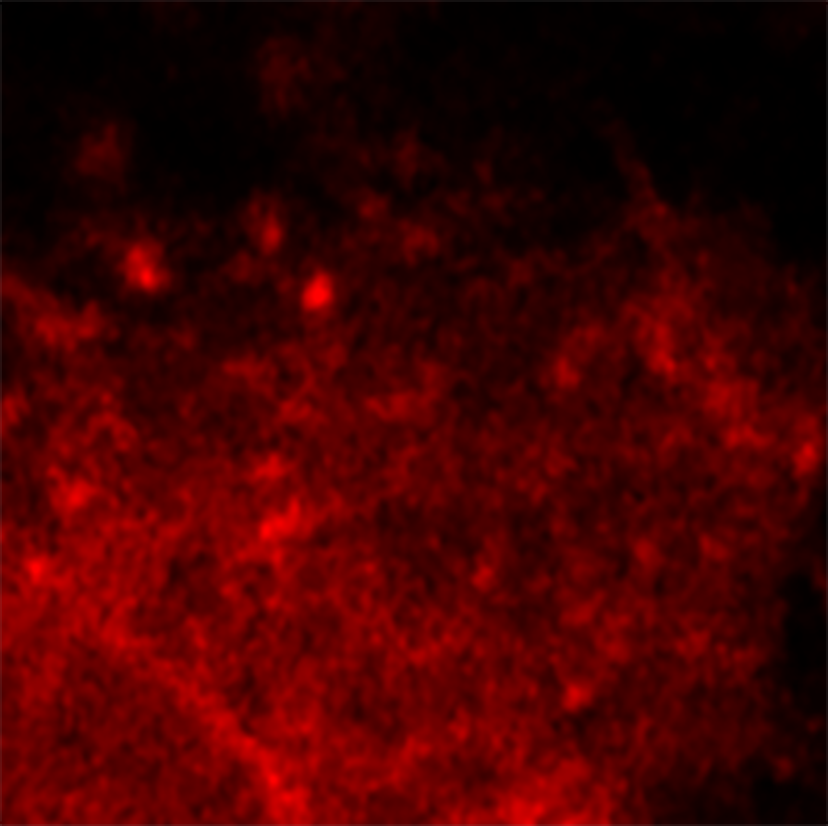

Supplement: Supplementary file 12 — Figures EV1-5 Source Data [file 44318_2025_410_MOESM12_ESM.zip › Source Data For EV Figures/Source Data For Figure EV5/EV5C, F _microscopy/EV5C/1. Cherry-ATG2A.tif]

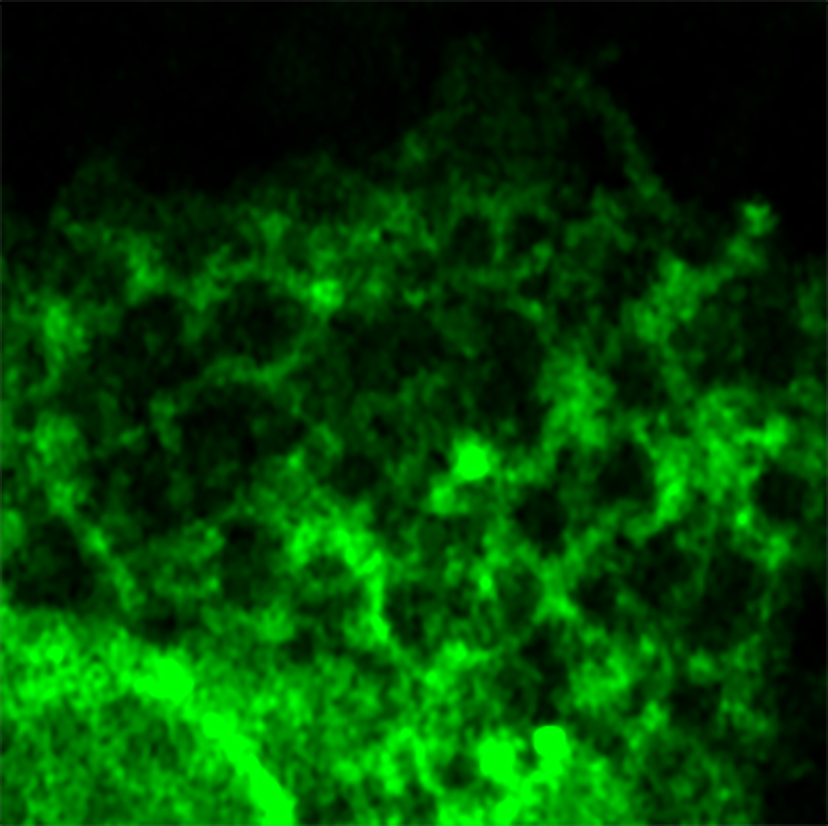

Supplement: Supplementary file 12 — Figures EV1-5 Source Data [file 44318_2025_410_MOESM12_ESM.zip › Source Data For EV Figures/Source Data For Figure EV5/EV5C, F _microscopy/EV5C/2. GFP-Sec61β.tif]

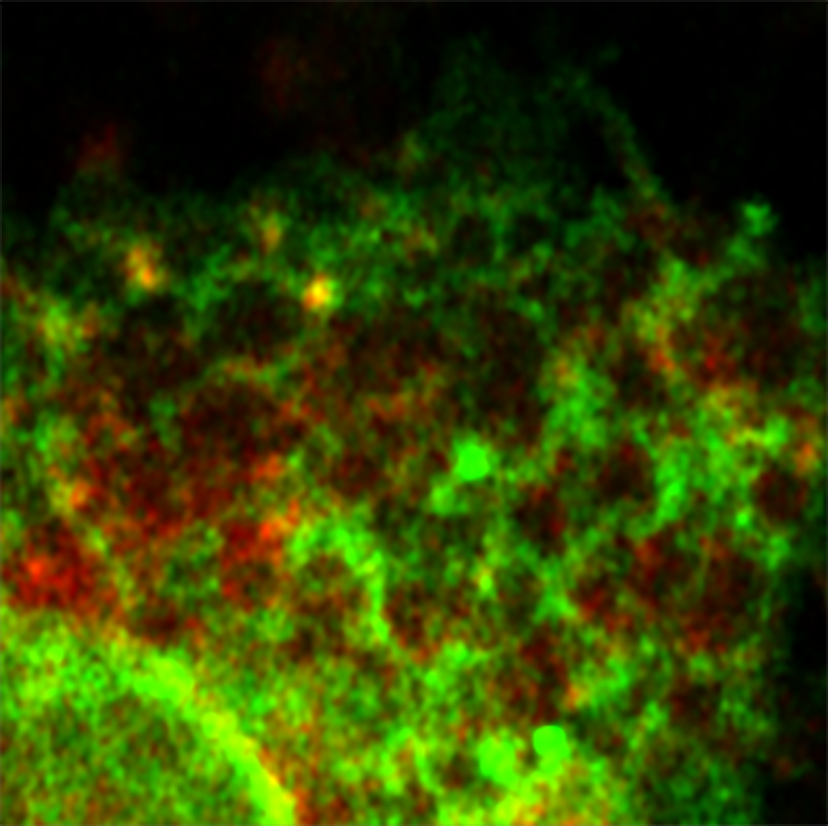

Supplement: Supplementary file 12 — Figures EV1-5 Source Data [file 44318_2025_410_MOESM12_ESM.zip › Source Data For EV Figures/Source Data For Figure EV5/EV5C, F _microscopy/EV5C/3. Merged.tif]

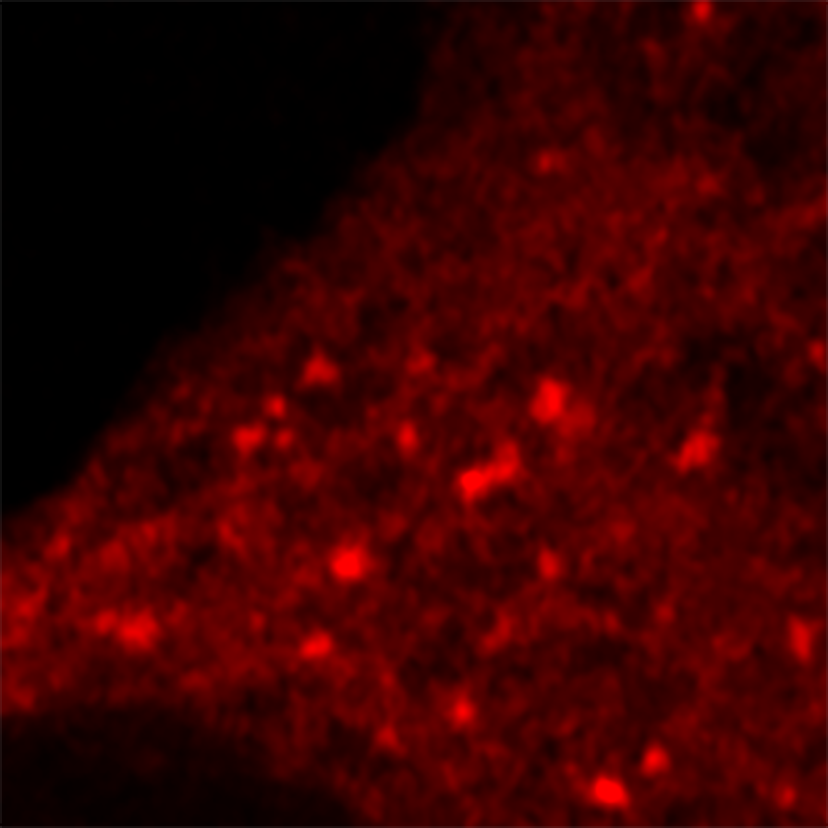

Supplement: Supplementary file 12 — Figures EV1-5 Source Data [file 44318_2025_410_MOESM12_ESM.zip › Source Data For EV Figures/Source Data For Figure EV5/EV5C, F _microscopy/EV5C/4. Cherry-ATG2A-3CS.tif]

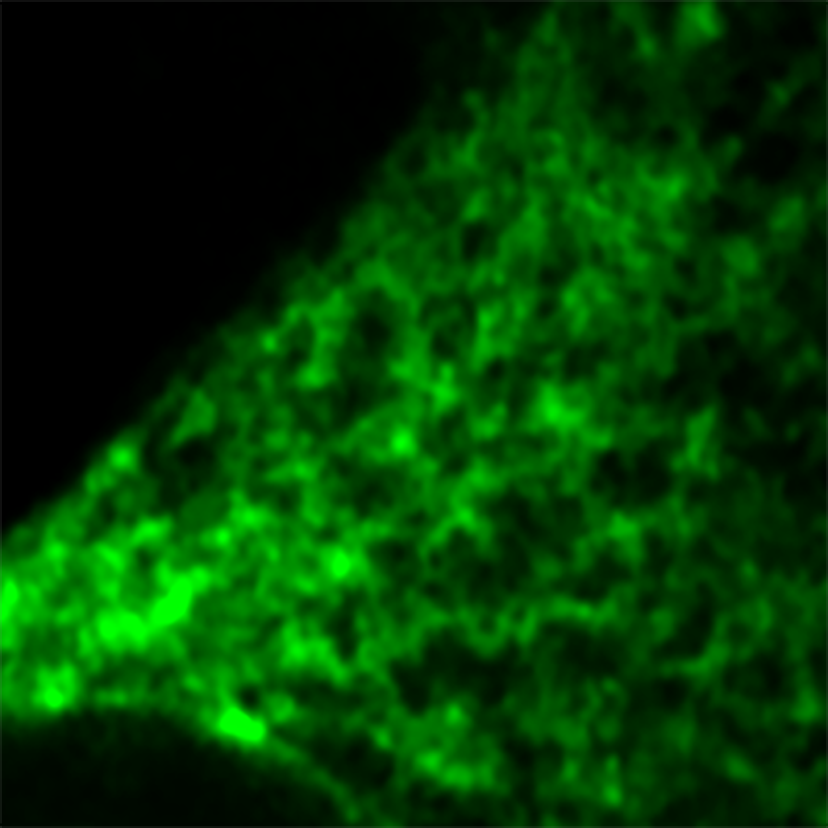

Supplement: Supplementary file 12 — Figures EV1-5 Source Data [file 44318_2025_410_MOESM12_ESM.zip › Source Data For EV Figures/Source Data For Figure EV5/EV5C, F _microscopy/EV5C/5. GFP-Sec61β.tif]

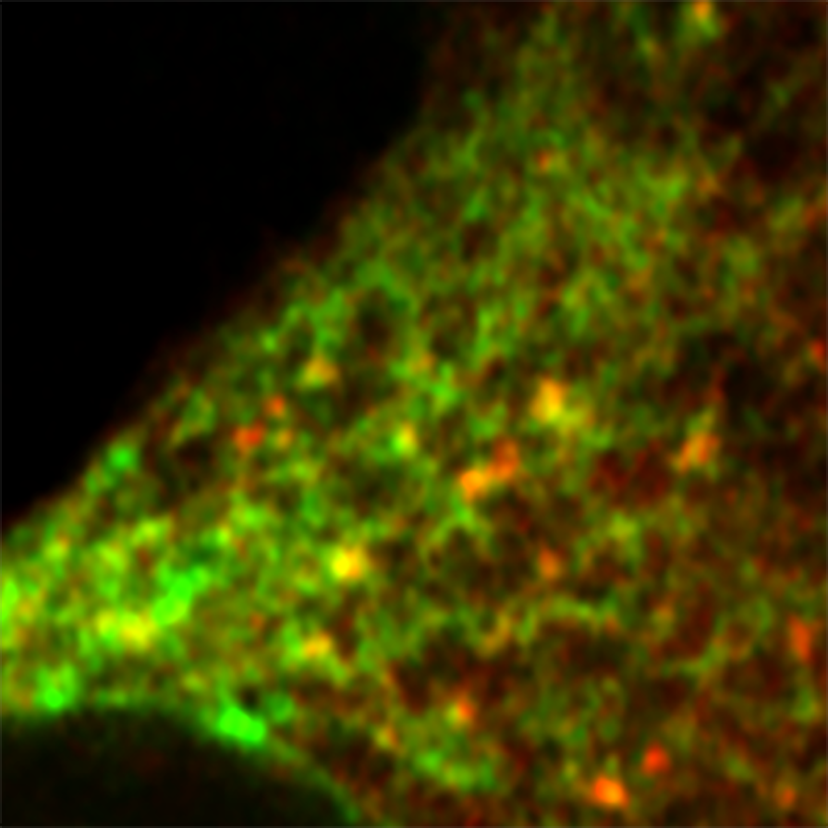

Supplement: Supplementary file 12 — Figures EV1-5 Source Data [file 44318_2025_410_MOESM12_ESM.zip › Source Data For EV Figures/Source Data For Figure EV5/EV5C, F _microscopy/EV5C/6. Merged.tif]

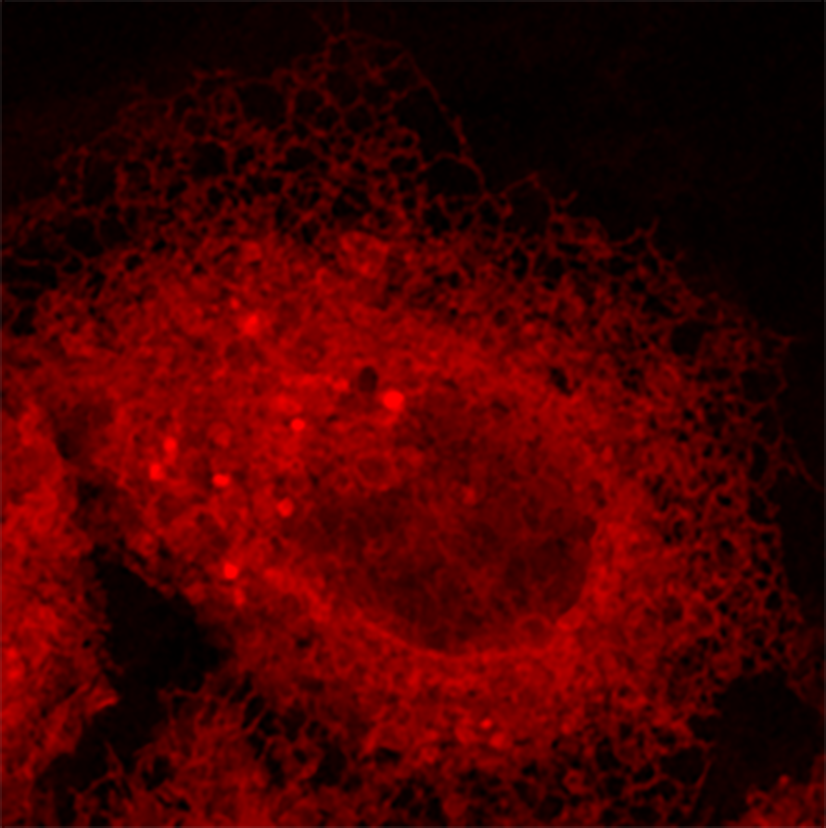

Supplement: Supplementary file 12 — Figures EV1-5 Source Data [file 44318_2025_410_MOESM12_ESM.zip › Source Data For EV Figures/Source Data For Figure EV5/EV5C, F _microscopy/EV5F/Cherry-Sec61β.tif]

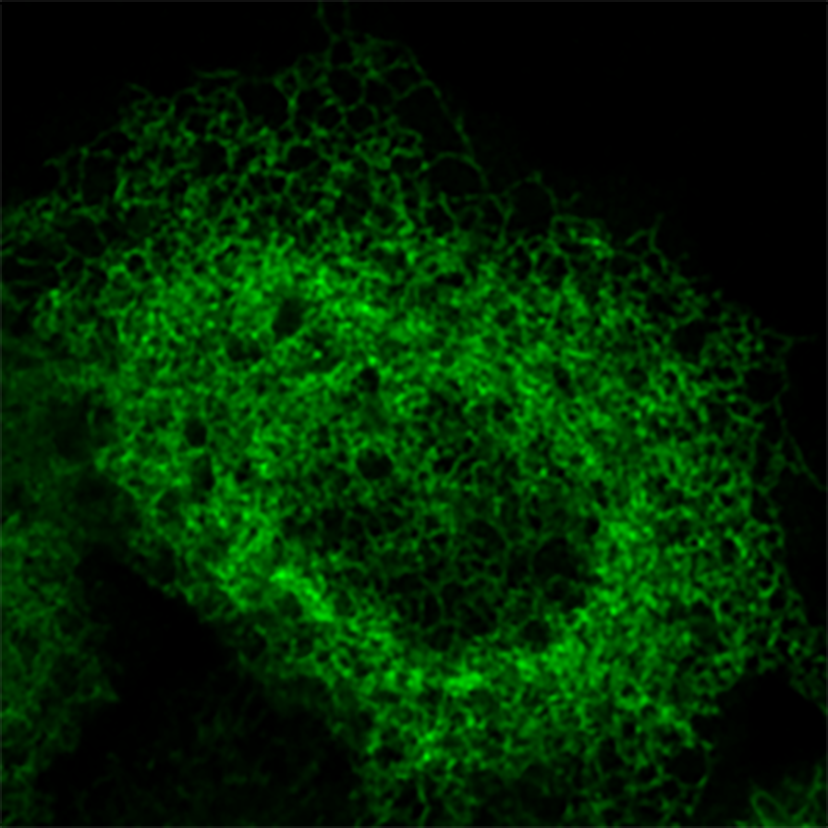

Supplement: Supplementary file 12 — Figures EV1-5 Source Data [file 44318_2025_410_MOESM12_ESM.zip › Source Data For EV Figures/Source Data For Figure EV5/EV5C, F _microscopy/EV5F/GFP-ATG2A-ER.tif]

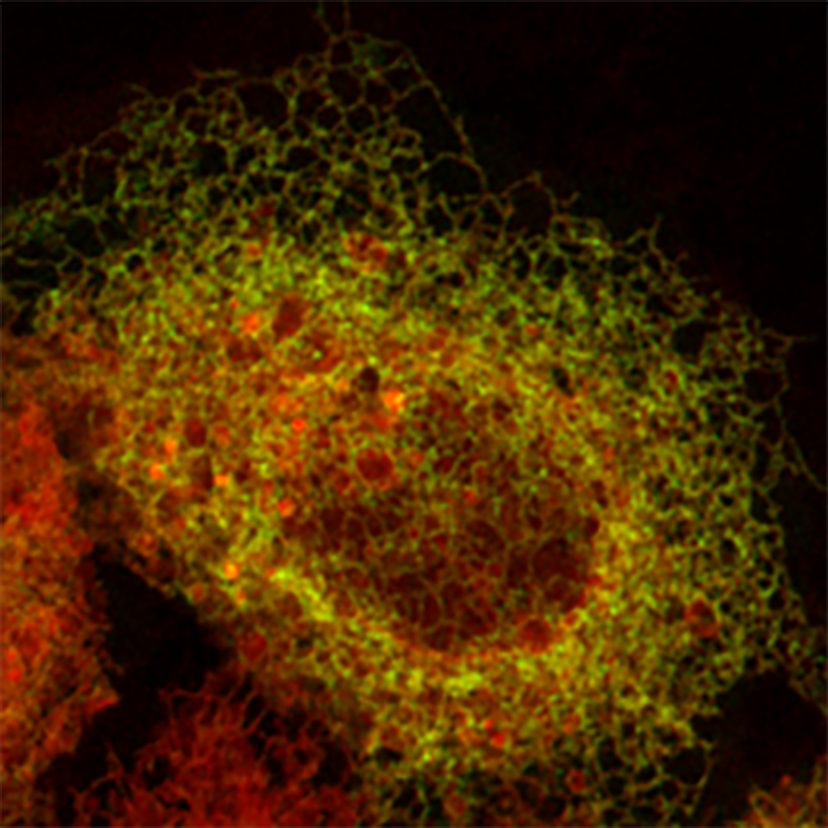

Supplement: Supplementary file 12 — Figures EV1-5 Source Data [file 44318_2025_410_MOESM12_ESM.zip › Source Data For EV Figures/Source Data For Figure EV5/EV5C, F _microscopy/EV5F/Merged.tif]
