# Supplementary material for: S-palmitoylation modulates ATG2-dependent non-vesicular lipid transport during starvation-induced autophagy
Source: EMBO J. 2025 Mar 24;44(9):2596–619. doi: 10.1038/s44318-025-00410-7 (PMC12048663; doi:10.1038/s44318-025-00410-7)
Supplement: Supplementary file 13 — Expanded View Figures [file 44318_2025_410_MOESM13_ESM.pdf]

Expanded View Figures

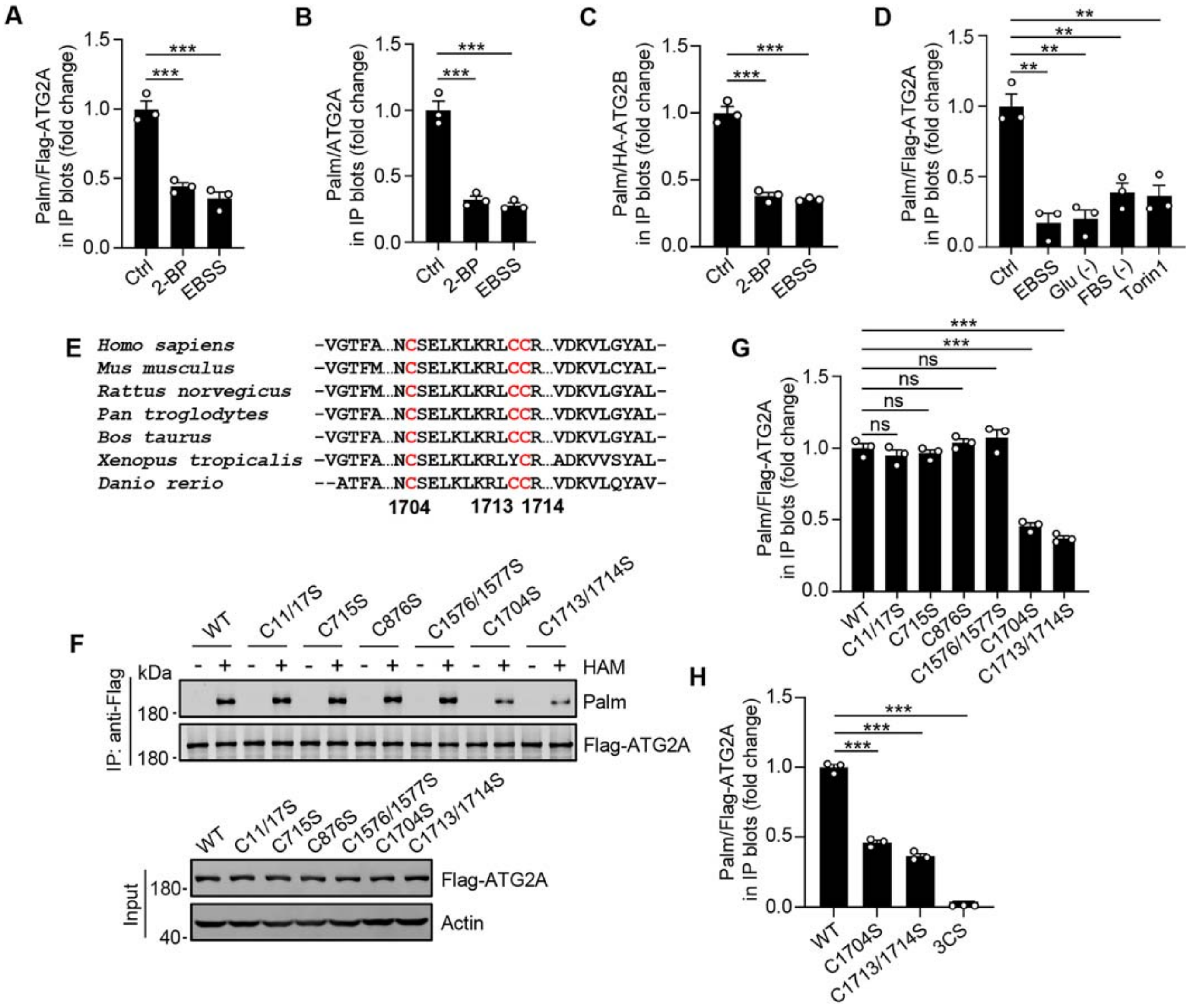

Figure EV1. Depalmitoylation of ATG2A in starved cells.

(**A–C**) Statistical analysis of S-palmitoylation levels for Flag-ATG2A (**A**), endogenous ATG2A (**B**) and HA-ATG2B (**C**) in Fig. 1A–C. Student's *t*-test was used to calculate *P* values. Exact *P* values for (**A**) from left to right: $P = 0.00096$; $P = 0.00097$. Exact *P* values for (**B**) from left to right: $P = 0.00084$; $P = 0.00058$. Exact *P* values for (**C**) from left to right: $P = 0.00034$; $P = 0.00019$. (**D**) Statistical analysis of S-palmitoylation levels for Flag-ATG2A in Fig. 1E. Student's *t*-test was used to calculate *P* values. Exact *P* values from left to right: $P = 0.00149$; $P = 0.00164$; $P = 0.00449$; $P = 0.00499$. (**E**) Conservation analysis of S-palmitoylation sites in ATG2A across species. (**F**) S-palmitoylation levels of Flag-ATG2A mutants measured by ABE assay in HEK293T cells. (**G**) Statistical analysis of (**F**). Student's *t*-test was used to calculate *P* values. Exact *P* values from left to right: $P = 0.38347$; $P = 0.44816$; $P = 0.40234$; $P = 0.30211$; $P = 0.00017$; $P = 7.48E{-}05$. (**H**) Statistical analysis of the S-palmitoylation level for Flag-ATG2A mutants in Fig. 1G. Student's *t*-test was used to calculate *P* values. Exact *P* values from left to right: $P = 3.80E{-}05$; $P = 2.24E{-}05$; $P = 1.41E{-}06$. Data information: All statistical data are presented as mean ± SEM of three independent experiments. ns, not significant; $^{**}P < 0.01$; $^{***}P < 0.001$ (Student's *t*-test). Source data are available online for this figure.

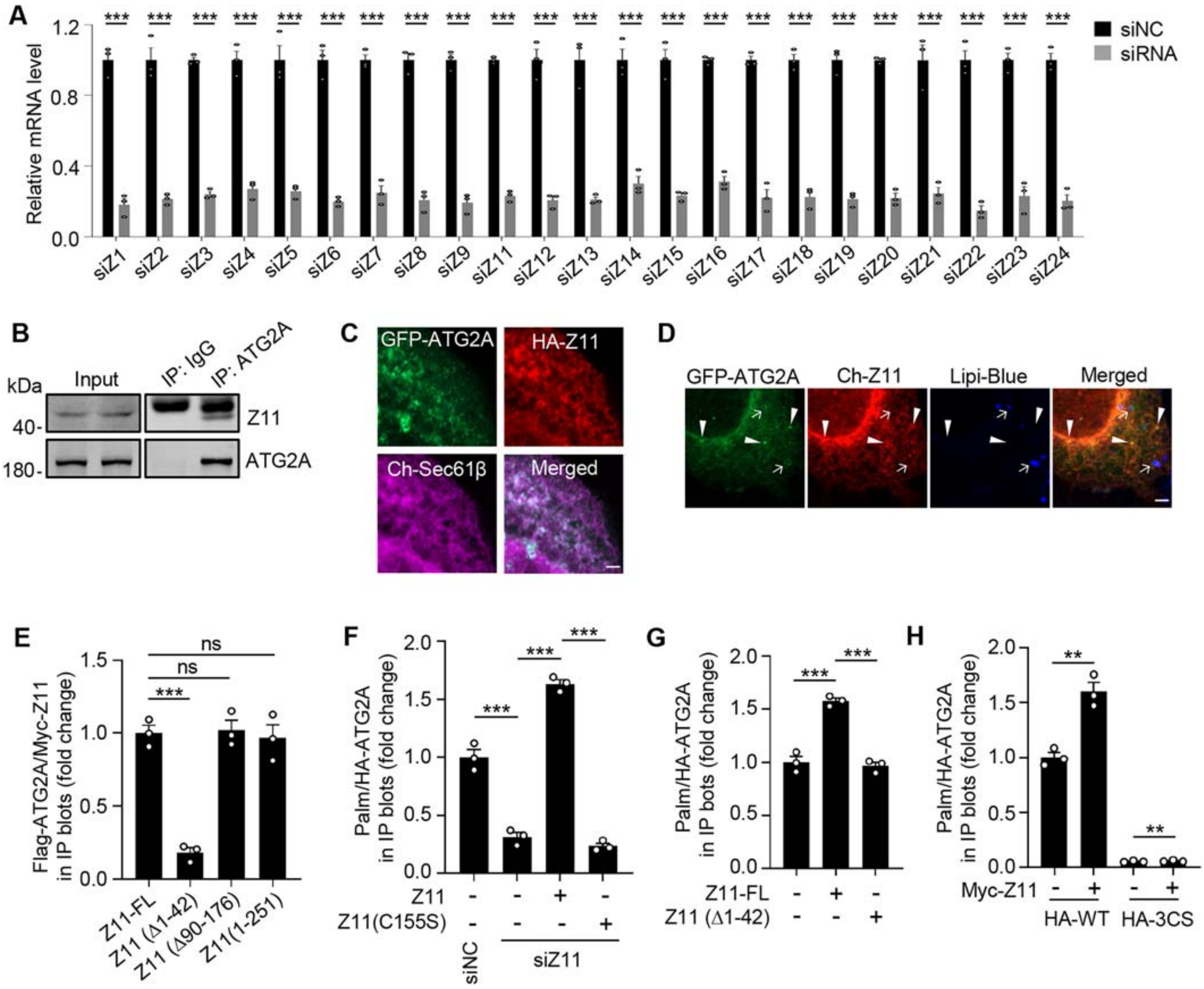

Figure EV2. ATG2A is S-palmitoylated by ZDHHC11.

(**A**) qPCR analysis of ZDHHC gene RNAi efficiency in HEK293T cells. qPCR primers for ZDHHC11 are designed to target ZDHHC11 and ZDHHC11B. Student's *t*-test was used to calculate *P* values. Exact *P* values from left to right: $P = 0.00018$; $P = 0.00040$; $P = 4.62E{-}06$; $P = 0.00026$; $P = 0.00096$; $P = 0.00017$; $P = 0.00012$; $P = 0.00012$; $P = 6.00E{-}05$; $P = 3.25E{-}06$; $P = 0.00027$; $P = 0.00064$; $P = 0.00069$; $P = 0.00025$; $P = 2.83E{-}05$; $P = 0.00011$; $P = 8.83E{-}05$; $P = 8.22E{-}05$; $P = 9.73E{-}06$; $P = 0.00122$; $P = 0.00015$; $P = 0.00031$; $P = 0.00011$. (**B**) Co-immunoprecipitation of endogenous ZDHHC11 and ATG2A in HEK293T cells. (**C**) Representative images showing the localization of GFP-ATG2A, HA-ZDHHC11 and Cherry-Sec61β. GFP-ATG2A, HA-ZDHHC11 and Cherry-Sec61β were transfected in HeLa cells cultured in complete media and the cells were imaged 48 h post-transfection. Scale bars, 2 μm. (**D**) Representative images showing the localization of GFP-ATG2A, HA-ZDHHC11 and lipid droplets. GFP-ATG2A and Cherry-ZDHHC11 were transfected in HeLa cells cultured in complete media and the cells were stained with Lipi-Blue and imaged 48 h post-transfection. Arrows point to the colocalization sites of ATG2A with LDs. Arrowheads indicate the sites of colocalization between ATG2A and ZDHHC11. Scale bar, 2 μm. (**E**) Statistical analysis of ratio of Flag-ATG2A to Myc-ZDHHC11 in IP blots for Fig. 2E. Student's *t*-test was used to calculate *P* values. Exact *P* values from left to right: $P = 0.00021$; $P = 0.82154$; $P = 0.77098$. (**F–H**) Statistical analysis of the S-palmitoylation level for HA-ATG2A in Fig. 2F–H. Student's *t*-test was used to calculate *P* values. Exact *P* values for (**F**) from left to right: $P = 0.00084$; $P = 1.47E{-}05$; $P = 5.63E{-}06$. Exact *P* values for (**G**) from left to right: $P = 0.00069$; $P = 0.00014$. Exact *P* values for (**H**) from left to right: $P = 0.00332$; $P = 0.50351$. Data information: All statistical data are presented as mean ± SEM of three independent experiments. ns, not significant; $^{**}P < 0.01$; $^{***}P < 0.001$ (Student's *t*-test). Source data are available online for this figure.

 © The Author(s)

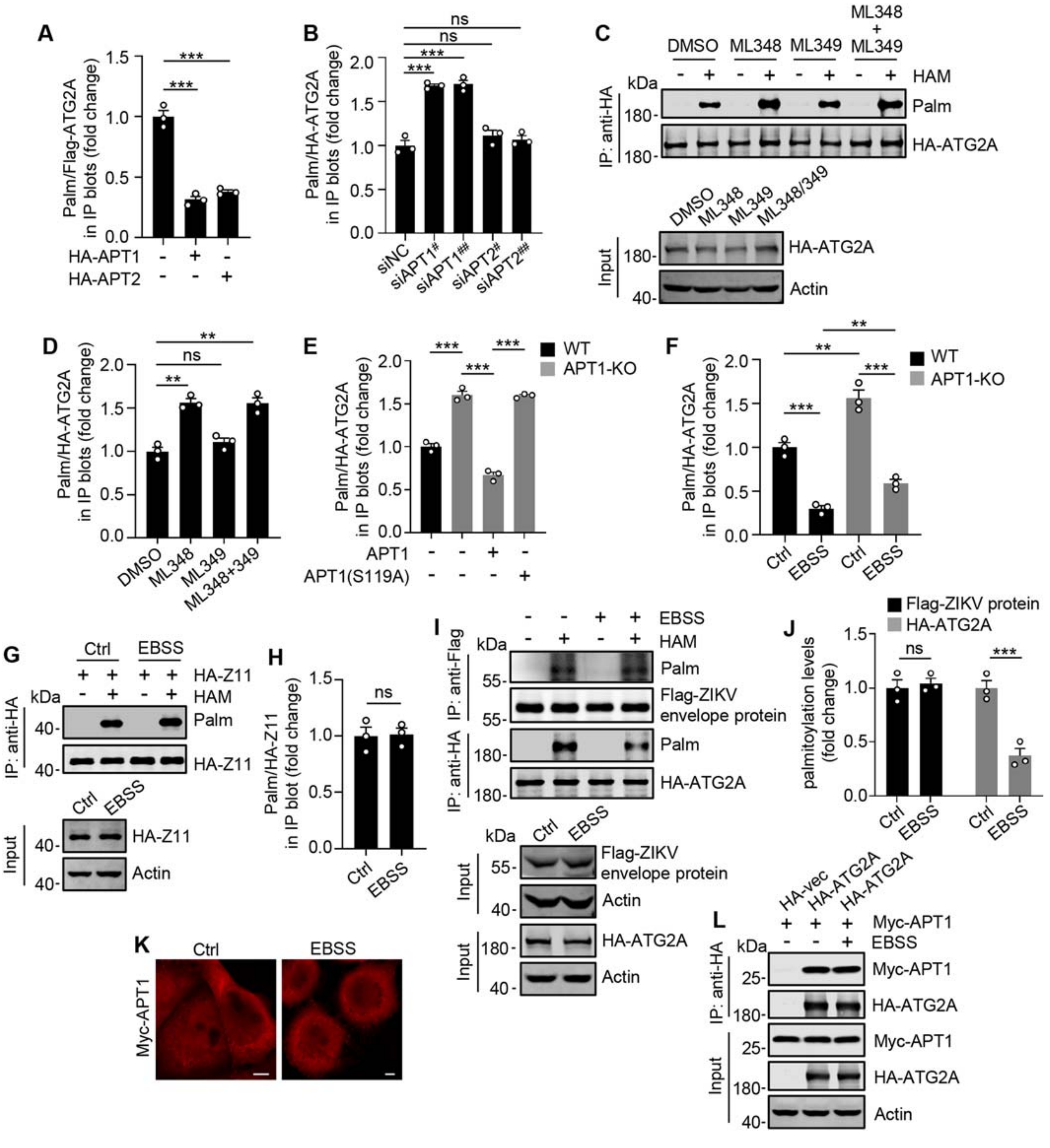
A
Palm/Flag-ATG2A in IP blots (fold change)
HA-APT1
HA-APT2
B
Palm/HA-ATG2A in IP blots (fold change)
siNC
siAPT1#
siAPT1##
siAPT2#
siAPT2##
C
DMSO
ML348
ML349
ML348 + ML349
HAM
IP: anti-HA
Palm
HA-ATG2A
Input
ML348/349
Actin
D
ML348+349
E
WT
APT1-KO
APT1
APT1(S119A)
F
Ctrl
EBSS
G
HA-Z11
H
Palm/HA-Z11 in IP blot (fold change)
I
IP: anti-Flag
Flag-ZIKV envelope protein
J
Flag-ZIKV protein
palmitoylation levels (fold change)
K
Myc-APT1
L
HA-vec
Myc-APT1
kDa

Figure EV3. APT1 is a depalmitoylase of ATG2A.

(**A**, **B**) Statistical analysis of the S-palmitoylation level for Flag-ATG2A or HA-ATG2A in Fig. 3C, D. Student's *t*-test was used to calculate *P* values. Exact *P* values for (**A**) from left to right: $P = 0.00032$; $P = 0.00034$. Exact *P* values for (**B**) from left to right: $P = 0.00037$; $P = 0.00069$; $P = 0.22506$; $P = 0.37955$. (**C**) S-palmitoylation of HA-ATG2A in HEK293T cells expressing HA-ATG2A. The cells were treated with specific APT inhibitor. (**D**) Statistical analysis of (**C**). Student's *t*-test was used to calculate *P* values. Exact *P* values from left to right: $P = 0.00107$; $P = 0.18353$; $P = 0.00233$. (**E**, **F**) Statistical analysis of the S-palmitoylation level for HA-ATG2A in Fig. 3E, F. Student's *t*-test was used to calculate *P* values. Exact *P* values for (**E**) from left to right: $P = 0.00039$; $P = 7.69E{-}05$; $P = 1.80E{-}05$. Exact *P* values for (**F**) from left to right: $P = 0.00046$; $P = 0.00665$; $P = 0.00576$; $P = 0.00067$. (**G**) ABE assay of HA-ZDHHC11 S-palmitoylation in HA-ZDHHC11-transfected HEK 293T cells cultured with or without EBSS medium. (**H**) Statistical analysis of (**G**). (**I**) S-palmitoylation of Flag-tagged ZIKV envelope protein and HA-ATG2A in HEK293T cells cultured with or without EBSS medium. (**J**) Statistical analysis of (**I**). Student's *t*-test was used to calculate *P* values. Exact *P* values from left to right: $P = 0.66553$; $P = 0.00287$. (**K**) Representative images showing the localization of Myc-APT1 in Hela cells with or without EBSS treatment. (**L**) Co-immunoprecipitation of APT1 with ATG2A. HA-ATG2A was immunoprecipitated from HA-ATG2A-expressing HEK293T cells transfected with Myc-APT1 with or without EBSS treatment. The precipitates were analyzed by Western blot using anti-Myc. Data information: All statistical data are presented as mean ± SEM of three independent experiments. ns, not significant; $^{**}P < 0.01$; $^{***}P < 0.001$ (Student's *t*-test). Source data are available online for this figure.

 © The Author(s)

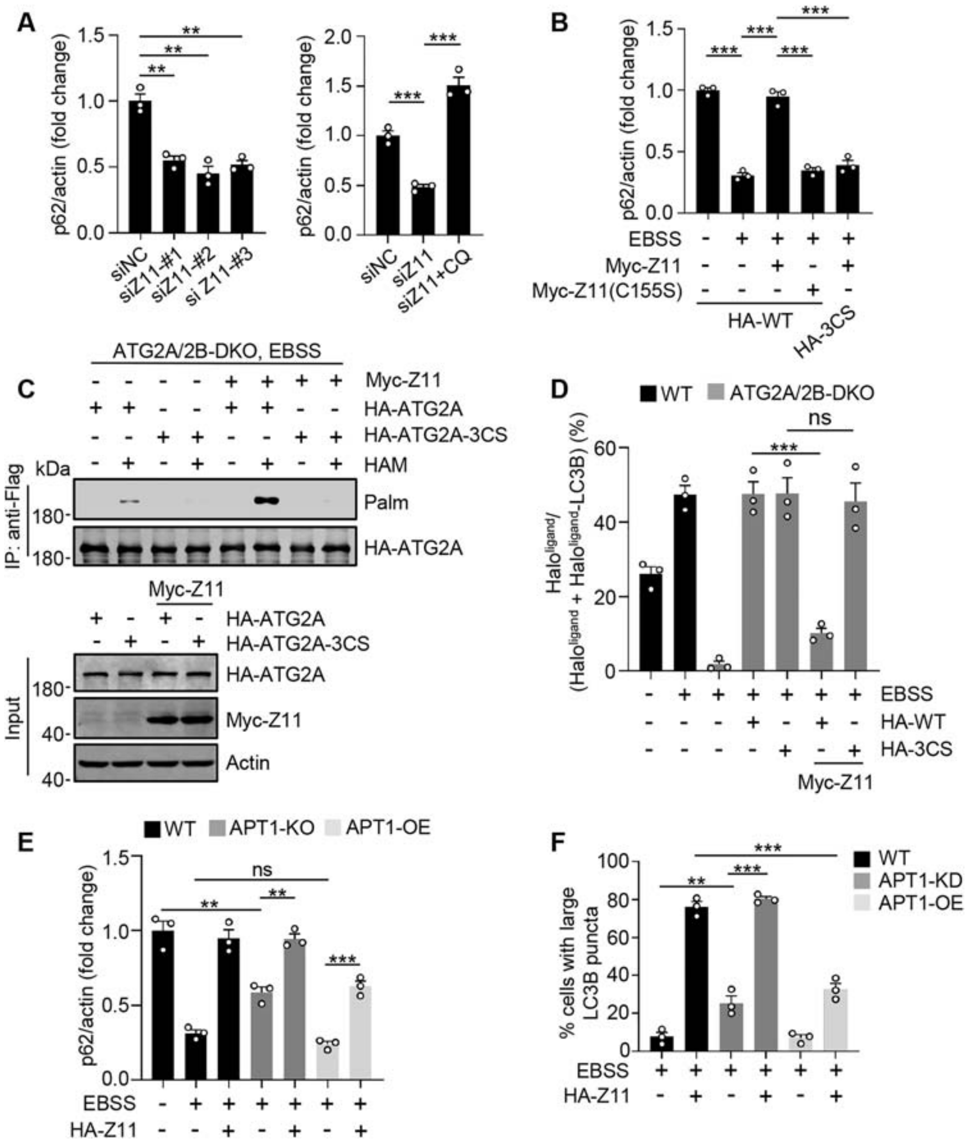

Figure EV4. Depalmitoylation of ATG2A in starvation-induced autophagy.

(**A**, **B**) Statistical analysis of p62 protein levels in Fig. 4A, C. Student's *t*-test was used to calculate *P* values. Exact *P* values for (**A**) from left to right: $P = 0.00203$; $P = 0.00196$; $P = 0.00150$; $P = 0.00065$; $P = 0.00026$. Exact *P* values for (**B**) from left to right $P = 2.23E{-}05$; $P = 0.00014$; $P = 0.00018$; $P = 0.00050$. (**C**) S-palmitoylation of HA-ATG2A or HA-ATG2A-3CS in ATG2A/2B-DKO NRK cells stably expressing HA-ATG2A or HA-ATG2A-3CS. The cells were transfected with or without Myc-ZDHHC11 and treated with EBSS. (**D**) Statistical analysis of Fig. 4H. Student's *t*-test was used to calculate *P* values. Exact *P* values from left to right: $P = 0.00050$; $P = 0.76680$. (**E**) Statistical analysis of p62 protein levels in Fig. 4I. Student's *t*-test was used to calculate *P* values. Exact *P* values from left to right: $P = 0.00514$; $P = 0.06602$; $P = 0.00209$; $P = 0.00064$. (**F**) Statistical analysis of the proportion of cells containing large LC3B puncta (diameter > 1.5 μm) in Fig. 4J. Student's *t*-test was used to calculate *P* values. Exact *P* values from left to right: $P = 0.01556$; $P = 0.00016$; $P = 0.00051$. Data information: All statistical data are presented as mean ± SEM of three independent experiments. ns, not significant; $^{**}P < 0.01$; $^{***}P < 0.001$ (Student's *t*-test). Source data are available online for this figure.

© The Author(s)

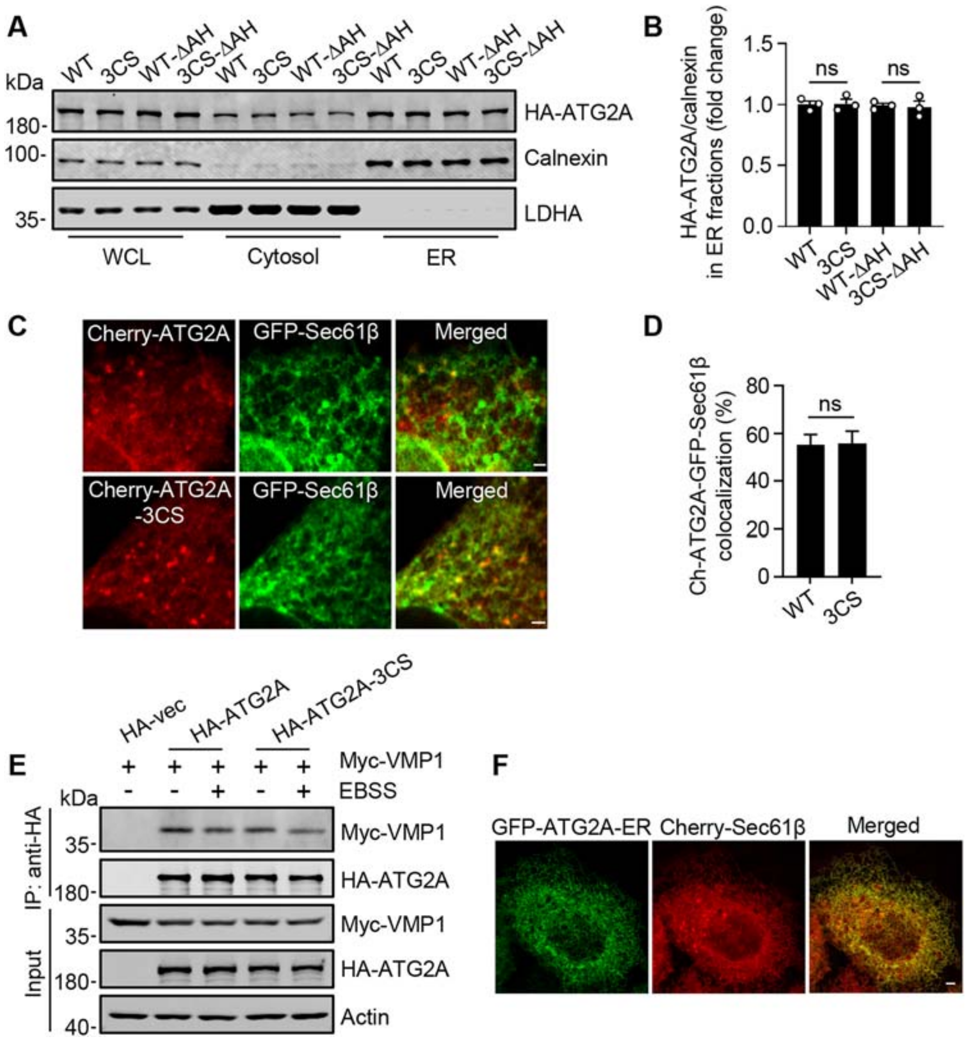

Figure EV5. S-palmitoylation anchors ATG2A C-terminal to the ER.

(**A**) Western blot analysis of the distribution of ATG2A and its mutants in subcellular fractions of ATG2A/2B-DKO NRK cells. Cells were transfected with HA-ATG2A, HA-ATG2A-3CS, HA-ATG2A-ΔAH, or HA-ATG2A-3CS-ΔAH. WCL: whole cell lysis. (**B**) Statistical analysis of (**A**). (**C**) Representative images showing the colocalization of Cherry-ATG2A or Cherry-ATG2A-3CS with GFP-Sec61β in HeLa cells. Scale bars, 1 µm. (**D**) Statistical analysis of (**C**). $n = 30$ cells. (**E**) Co-immunoprecipitation of VMP1 with ATG2A or ATG2A-3CS. HA-ATG2A or HA-ATG2A-3CS was immunoprecipitated from HEK293T cells co-transfected with Myc-VMP1. The cells were treated with or without EBSS, and the precipitates were analyzed by Western blot using anti-Myc. (**F**) Representative images showing the colocalization of GFP-ATG2A-ER and Cherry-Sec61β in HeLa cells. Scale bar, 2 µm. Data information: All statistical data are presented as mean ± SEM of three independent experiments unless otherwise specified. ns, not significant (Student's *t*-test). Source data are available online for this figure.

 © The Author(s)
